# Improved Utilization of ADAS-Cog Assessment Data Through Item Response Theory Based Pharmacometric Modeling

**DOI:** 10.1007/s11095-014-1315-5

**Published:** 2014-03-05

**Authors:** Sebastian Ueckert, Elodie L. Plan, Kaori Ito, Mats O. Karlsson, Brian Corrigan, Andrew C. Hooker, the Alzheimer’s Disease Neuroimaging Initiative

**Affiliations:** 1 Pharmacometrics Research Group Department of Pharmaceutical Biosciences, Uppsala University, P.O. Box 591, SE-751 24 Uppsala, Sweden; 2 Metrum Research Group, Tariffville, Connecticut 06081 USA; 3 Primary Care Business Unit, Pfizer Inc, Groton, Connecticut 06340 USA

**Keywords:** ADAS-cog, Alzheimer’s disease, item response theory, nonlinear mixed effect models, pharmacometrics

## Abstract

**Purpose:**

This work investigates improved utilization of ADAS-cog data (the primary outcome in Alzheimer’s disease (AD) trials of mild and moderate AD) by combining pharmacometric modeling and item response theory (IRT).

**Methods:**

A baseline IRT model characterizing the ADAS-cog was built based on data from 2,744 individuals. Pharmacometric methods were used to extend the baseline IRT model to describe longitudinal ADAS-cog scores from an 18-month clinical study with 322 patients. Sensitivity of the ADAS-cog items in different patient populations as well as the power to detect a drug effect in relation to total score based methods were assessed with the IRT based model.

**Results:**

IRT analysis was able to describe both total and item level baseline ADAS-cog data. Longitudinal data were also well described. Differences in the information content of the item level components could be quantitatively characterized and ranked for mild cognitively impairment and mild AD populations. Based on clinical trial simulations with a theoretical drug effect, the IRT method demonstrated a significantly higher power to detect drug effect compared to the traditional method of analysis.

**Conclusion:**

A combined framework of IRT and pharmacometric modeling permits a more effective and precise analysis than total score based methods and therefore increases the value of ADAS-cog data.

**Electronic supplementary material:**

The online version of this article (doi:10.1007/s11095-014-1315-5) contains supplementary material, which is available to authorized users.

## Introduction

The Alzheimer’s Disease Assessment Scale—cognitive subscale (ADAS-cog) has served as the de-facto standard for the assessment of cognition in clinical trials in mild to moderate Alzheimer’s Disease (AD) patients for the past 20 years (1). However, recent failures of promising drug candidates developed for the treatment of AD, and a movement towards earlier forms of the disease for future studies, have led some to question the sensitivity of the ADAS-cog. The ADAS-cog score has proven to be non-uniformly sensitive to measuring cognitive decline in AD across mild to moderate patients and has been recognized as less appropriate for earlier stages of the disease (2). Recently, new cognitive tests, with a specific focus on early AD, have been proposed (2). While likely providing more specificity, the disadvantages of continued introduction of novel tests and the inflation of the numbers and types of assessments are rarely considered. Fragmentation of cognitive assessments into specific measures for specific stages of AD reduces the comparability of study outcomes for things like comparative effectiveness research, and diminishes the possibility to acquire useful knowledge across the lifespan of an individual patient, or across trials. As earlier AD populations are studied, the trial duration must increase to studies of at least 2 to 4 years duration; coupled with enrolment times and open-label extension studies, future drug development programs can involve investigations as long as 8 years for a patient. As trials increase in duration, or as trial populations are enriched by biomarker or imaging criteria to select more rapid progressors, the variability and sensitivity of a highly specific cognitive assessment may increase if the newly added items become non-sensitive (i.e., floor and/or ceiling effects). There is also the risk of lower assessment completion rate, improper application by raters due to lack of familiarity with the test, and improper scoring for missing data due to the lack of standardized scoring algorithms across organizations for the new instrument.

An alternative to generating multiple cognitive tests that target different individual short-period AD populations, is to utilize subsets of one or more of the existing assessments that are most sensitive in that population. Longitudinal patient ADAS-cog score data contains an inherent temporal and hierarchical structure, with various items within the ADAS-cog having more or less sensitivity at various stages of the disease. Utilizing a single assessment tool like the ADAS-cog, and focusing only on the most sensitive sub scores allows information from various studies to be easily combined, leverages the large mass of existing data sources as a useful prior, and provides a platform for following changes in cognition over a wide range of disease severity. The question is how to determine which subsets to use and when, and how scores from various ADAS-cog variants can provide a continuous description of change in disability over time.

Recent publications have demonstrated that an increase in precision in the cognitive assessment is achievable through application of the statistical framework of item response theory (IRT) (3,4). IRT uses a hidden or latent variable approach to describe the unobservable quantity an assessment aims to measure (5). In this manuscript we focus on the ADAS-cog and the interpretation of each task of the assessment as a surrogate measure for the patient’s *cognitive disability*. The relationship between the outcome of the tasks and *cognitive disability* is characterized through item characteristic curves (ICCs). The shape of these curves expresses how informative each question is in relation to the population being tested. Patients’ most likely *cognitive disability* can be estimated and compared on a single scale irrespective of the test they took, as long as the individual questions have been mapped to the overall *cognitive disability* of the population. This aspect makes the IRT approach preferable for measuring cognition over a long period of time with either one instrument, an instrument with multiple variants (ADAS-cog 11, 13 etc.) or multiple measurement instruments. Thus, IRT does not represent a novel cognitive assessment but an approach capable of a better utilization of existing data.

Another component of this work involves the application of pharmacometric methodologies to describe the change in cognitive disability over time. A combination of the advantages of IRT with the statistical benefits and versatility of pharmacometric models appear to be ideally suited to improve the utilization of ADAS-cog assessment data and address a number of the challenges described above.

This work investigates the four hypotheses in combining pharmacometric modeling and IRT for the analysis of ADAS-cog data. Specifically, a combined pharmacometric and IRT approach is (i) suitable for describing the baseline cognitive ability of Mild and moderate AD patients assessed with different ADAS-cog variants, (ii) increases the efficiency of future trials by optimizing the most informative subset of cognitive tests for a specific population, (iii) improves the description of longitudinal changes in cognition data, like that from long term clinical trials, and (iv) enhances the sensitivity to detect changes in cognition.

## Materials and Methods

### Assessment of Baseline Cognition from Multiple Data Sources

An IRT model describing ADAS-cog assessment data was developed from clinical trial databases.

#### Data

The data were taken from two major AD databases, the Alzheimer’s Disease Neuroimaging Initiative (ADNI) and the Coalition Against Major Diseases (CAMD). The data contained in the ADNI database^1^ (http://adni.loni.ucla.edu/ database version as of December 2012) consisted of a mild cognitively impairment (MCI) group, a mild AD group and an elderly control group that were followed for 36 months (24 for the control group). The item level ADAS-cog assessment data at baseline (first non-screening measurement at the beginning of the study) from all groups were used in this work. The CAMD database (C-Path Online Data Repository) contains, as of November 2012, the de-identified control arm data from 20 clinical trials (http://www.c-path.org/camd.cfm). Eight out of those 20 trials contained both total ADAS-cog score and item level data. The baseline data from seven of these eight studies were used to develop the IRT model, the remaining study was kept for the longitudinal analysis (described herein).

As a collection of various studies, these two databases contain a number of different ADAS-cog assessment variants differing in the number of components included in the assessment. The original ADAS-cog assessment developed by Rosen *et al.* (6) consists of 11 components: 6 task based (“Commands”, “Construction”, “Ideational Praxis”, “Naming Object & Fingers”, “Orientation”, “Word Recall” and “Word Recognition”) and 4 rater assessed (“Comprehension”, “Spoken Language”, “Remembering” and “Word Finding”). A Common modification to the original ADAS-cog 11 is the addition of the components “Delayed Word Recall” and “Number Cancellation” as described by Mohs *et al.* (7). Overall, baseline ADAS-cog assessment data from 2,744 patients were used resulting in more than 120,000 individual data items. Table I lists the individual studies used in the analysis and their contribution in number of subjects as well as in terms of test components. The “Word Recognition” component for three studies had to be excluded from the analysis as results were stored only as recalled/not-recalled and not as correct/incorrect.

**Table I:** Characteristics of Studies Used in the Development of the Baseline IRT Model. The Numbers for CAMD Studies are Unique Study Identifiers in the CAMD Database

Study	Subjects	Additional^a^	Excluded^b^
ADNI	819	DWR, NC	–
CAMD 1131	57	DWR	NOF, WRC
CAMD 1132	412	DWR	WRC
CAMD 1137	216	–	–
CAMD 1138	202	–	–
CAMD 1140	137	DWR	NOF, WRC
CAMD 1141	492	DWR, NC	–
CAMD 1142	409	DWR, NC	–

^a^Components with item level data in addition to ADAS-cog 11 (*DWR* delayed word recall, *NC* number cancellation)

^b^Available in the data but not analyzed (*NOF* naming objects & fingers, *WRC* word recognition)

#### Model

The IRT model described the response for each of the test items of the ADAS-cog as a function of the patients underlying *cognitive disability*. In the following section *a*_*j*_, *b*_*j*_, *c*_*j*_, *d*_*j*_ denote test specific parameters of test *j* and *D*_*i*_ refers to the unobserved *cognitive disability* of subject *i*.

Most ADAS-cog test items consist of a number of tasks that the subject is asked to perform and whether the patient succeeded or not. These tests with two potential outcomes were modeled using a binary model (8), describing the probability to fail (*p*_*ij*_) as function of *cognitive disability* using a three-parameter model, commonly referred to as a 3PL (3 parameter logit) in IRT publications. The utilized 3PL model had the form

1 $$ {p}_{ij}={c}_j+\left(1-{c}_j\right)\frac{e^{a_j\left({D}_i-{b}_j\right)}}{1+{e}^{a_j\left({D}_i-{b}_j\right)}} $$

In this parameterization the three test item specific parameters are: *a*_*j*_—the slope or discrimination parameter, *b*_*j*_—the item location or difficulty parameter and *c*_*j*_—the probability for a subject with no cognitive impairment to fail.

For word based tests of the ADAS-cog assessment, “(Delayed) Word Recall” and “Word Recognition”, it was assumed that the ICCs do not differ between words. The resulting count of incorrectly recalled/recognized words (*k*) out of *n* given words, was described using the binomial model (9)

2 $$ P\left({Y}_{ij}=k\right)=\left(\begin{array}{c}\hfill n\hfill \\ {}\hfill k\hfill \end{array}\right){p}_{ij}^k{\left(1-{p}_{ij}\right)}^{n-k} $$

where $$ \left(\begin{array}{c}\hfill n\hfill \\ {}\hfill k\hfill \end{array}\right) $$ denotes the binomial coefficient. For the word recall tests (3 repetitions of the word recall test and the delayed word recall test), the failure probability *p*_*ij*_ was modeled using Eq. (1). For the “Word Recognition” test, Eq. (1) was extended to

3 $$ {p}_{ij}={c}_j+\left({d}_j-{c}_j\right)\frac{e^{a_j\left({D}_i-{b}_j\right)}}{1+{e}^{a_j\left({D}_i-{b}_j\right)}} $$

where the additional parameter *d*_*j*_ describes the maximal probability for a severely cognitively impaired person to incorrectly categorize the words as previously seen or not. Due to slight differences in the implementation of the test as well as in the method of storing test results, some study specific modifications were incorporated in the word recognition test model. For CAMD studies 1141 and 1142 the binomial distribution was truncated at a count of 12 and for CAMD studies 1137 and 1138 separate difficulty and discrimination parameters were estimated to account for the 3-fold repetition of the test.

For the “Number Cancellation” component a generalized Poisson model (10) was used to describe the data:

4 $$ P\left({Y}_{ij}=k\right)=\frac{p\left({D}_i\right){\left(p\left({D}_i\right)+\delta k\right)}^{k-1}{e}^{-p\left({D}_i\right)-\delta k}}{k!P\left({Y}_{ij}>40\right)} $$

5 $$ p\left({D}_i\right)={d}_j\left(1-\frac{e^{a_j\left({D}_i-{b}_j\right)}}{1+{e}^{a_j\left({D}_i-{b}_j\right)}}\right) $$

where *a*_*j*_, *b*_*j*_, *d*_*j*_ have a similar interpretation as above and *δ* is a dispersion parameter allowing for over- or underdispersion in the data. The factor *P*(*Y*_*ij*_ > 40) in Eq. (5) ensures that all scores predicted by the equation are in the range 0–40.

The remaining components are examiner rated, categorize a subject in one of five categories (no impairment to severe impairment) and were modeled using a proportional odds, ordered categorical model (11). The probability that a patient received a rating of at least *k* was described using the function

6 $$ P\left({Y}_{ij}\ge k\right)=\frac{e^{a_j\left({D}_i-{b}_{j,k}\right)}}{1+{e}^{a_j\left({D}_i-{b}_{j,k}\right)}} $$

Similar to the 3PL model, *a*_*j*_ is the slope and *b*_*j*,*k*_ is the difficulty parameter. The latter was constrained to be non-decreasing for higher scores of the same test (i.e., *b*_*j*,*k* + 1_ ≥ *b*_*j*,*k*_). The probability of obtaining exactly the score *k* was then calculated by subtracting the probability to obtain at least *k* + 1 from the probability of obtaining at least *k* (*P*(*Y*_*ij*_ = *k*) = *P*(*Y*_*ij*_ >  = *k*) − *P*(*Y*_*ij*_ >  = *k* + 1).

The variable *D*_*i*_ was modeled as a subject specific random effect following a normal distribution with a mean of zero and a variance of 1. Note that the assumed scale of *cognitive disability* goes from −∞ to + ∞. This scale is arbitrary and the theory does not preclude the use of other scales or assumed distributions.

#### Estimation and Validation

Implementation and parameter estimation of the model specified above was performed in NON-linear Mixed Effect Model software (NONMEM) version 7.3 beta (12) (NONMEM code is available upon request). All 169 test specific parameters were jointly estimated using the second-order conditional estimation method (Laplacian).

For each test item, the model fit was evaluated by graphically comparing the estimated ICC to the fit of a generalized additive model (GAM) using a cross-validated cubic spline as a smoothing function (13). This comparison was carried out for the pooled data as well as on a per study basis in order to identify study specific deviations. Furthermore, the final model was evaluated through simulation based diagnostics by using the ICC and the individual *cognitive disability* estimates to simulate responses (100 replicates) and subsequently compare them with the observations.

### Increasing Trial Efficiency by Selection of a Sensitive Subtest

The Fisher information of each assessment item was calculated and, using optimal design theory the most informative test components were determined in two patient populations.

#### Fisher Information for Cognitive Disability

The primary quantity of interest in this work was a patient’s cognition, captured as *cognitive disability* in Eqs. 1–6 above. From those equations the Fisher information for *cognitive disability* was calculated as minus the expectation of the second derivative of the log-likelihood. For the four different models used in this work (binary, binomial, Poisson and ordered categorical), this could be done analytically due to the special structure of the equations (i.e., the only random effect in the equation is *cognitive disability*). The resulting information functions were visualized to illustrate the sensitivity of each assessment item over the full *cognitive disability* range.

#### Population Specific Information

The Fisher information functions also served as a basis to calculate the average information of each assessment component in a MCI and a mild AD patient population. Firstly, mean and standard deviation for *cognitive disability* in the MCI and mild AD cohort of the ADNI study were estimated using the model described herein. Secondly, using the two disability distributions and assuming normality the average information for each ADAS-cog assessment item was calculated. Thirdly, average item information for all items in one component was added to yield average component information. Finally, assessment components were ranked based on their average information content.

### Application of IRT to Describe Longitudinal Changes in Cognition

An example dataset with data from a phase III trial was used to investigate the extension of the IRT based framework to longitudinal data.

#### Data

The data used to describe longitudinal changes in *cognitive disability* were collected from a double-blind, placebo controlled phase III trial to evaluate atorvastatin (Lipitor®) effect in mild to moderate AD patients who were on stable donepezil (Aricept®) background therapy (LEADe study) (14,15). The study duration was 80 weeks including a withdrawal phase of 8 weeks.

This analysis included all ADAS-cog 11 assessment data from the placebo arm (placebo + donepezil) during a double-blind treatment period and the withdrawal phase. The study included a total of eight scheduled ADAS-cog assessments, at 0, 3, 6, 9, 12, 15, 18 and 20 month. Overall, the dataset consisted of 322 patients contributing 98,439 item level observations.

The LEADe study is available in the CAMD database but was excluded when building the baseline model, to emulate the analysis of future studies.

#### Model

Test specific parameters in the baseline IRT model were fixed to the previously estimated baseline values and deterioration as a consequence of disease progression was implemented on the hidden variable. The specific implementation of the disease progression model on the hidden variable (*D*_*i*_) scale followed the model evaluated by Ito *et al.* (16). Total change during the study was assumed to be of small magnitude, justifying the following linear expression

7 $$ {D}_i(t)={D}_i^0+{a}_i\cdot t $$

Both the baseline *D*_*i*_^0^ and slope parameter *a*_*i*_ were assumed to be subject-specific and modeled through random effects (*D*_*i*_^0^ = *θ*_1_ + *η*_*i*1_ and *a*_*i*_ = *θ*_2_ + *η*_*i*2_ ), which were allowed to be correlated.

A dropout model for interval censored data (17) was implemented, describing the probability of a subject to remain in the study beyond a certain time. During the analysis, four different hazard functions (*h*(*x*,*t*)) were tested: constant hazard (log *h*(*x*,*t*) = *θ*_3_), *cognitive disability* dependent hazard (log *h*(*x*,*t*) = *θ*_3_ + *θ*_4_ ⋅ *D*_*i*(*t*)_), progression rate dependent hazard (log *h*(*x*,*t*) = *θ*_3_ + *θ*_4_ ⋅ *a*_*i*_) and baseline disability dependent hazard (log *h*(*x*,*t*) = *θ*_3_ + *θ*_4_ ⋅ *D*_*i*_^0^). The hazard function describing the data best, was chosen using the log-likelihood ratio test with a 5% significance level.

#### Estimation and Validation

The longitudinal IRT model was implemented in NONMEM 7.3 beta (12) and all parameters were estimated from the data using the Laplacian estimation method (NONMEM code is available upon request).

The adequacy of this model to describe clinical trial data was tested by comparing model predicted baseline ADAS-cog scores and annual rates of change to those values reported independently for the study (14). Furthermore, observed and predicted data were compared through visual predictive checks (VPCs) both on the aggregate ADAS-cog score level and on the individual item level. For the item level, 200 Monte-Carlo simulations from the final model and original study design were obtained and the proportion of patients in the observed data with a certain test outcome were compared to the 95% prediction interval of the simulated data. Similarly for the aggregate level, 200 datasets from the final IRT model were simulated, the ADAS-cog score calculated and the 95% confidence interval for the median, the 2.5th and 97.5th percentile from the simulated scores were compared to the corresponding observed percentiles. Additionally, observed and predicted dropout patterns were compared.

### Increasing Drug Effect Detection Power

Clinical trial simulations (CTSs) were used to compare the power to detect a drug effect for three different data analysis methods: (1) least-squares (LS) mean analysis, (2) analysis using a longitudinal pharmacometric model for the total ADAS-cog score and (3) analysis using the longitudinal IRT model.

#### Clinical Trial Simulations

The CTSs were performed under the scenario of a hypothetical phase III trial in mild to moderate AD patients for a disease modifying agent. The trial duration was set to 20 months with a balanced parallel-arm study design (placebo and treatment) and seven ADAS-cog assessments per subject (0, 3, 6, 9, 12, 15 and 18 month). Study sizes of 100, 200, 400 and 800 subjects in total were investigated in the simulations (for the LS-means analysis also 1,200 subjects). The longitudinal IRT model described herein with the parameter values estimated from the longitudinal data was used as the simulation model. A hypothetical drug effect was introduced as a 20% lower subject-specific disease progression rate in the treatment group (i.e. *a*_*i*_ = (1–0.2 ⋅ *x*_*Grp*_) ⋅ (*θ*_2_ + *η*_*i*2_), *x*_*Grp*_ = 0 in the placebo and *x*_*Grp*_ = 1 in the treatment group). For simplicity, it was assumed that patients did not drop out from the study. For each sample size, 500 clinical trials under the outlined trial design were simulated and subsequently analyzed with the three data analysis models described below. Finally, all power curves (power to detect a drug effect *versus* number of individuals) were compared graphically.

In addition to the power assessment, the type I error under each sample size and for all three analysis methods was investigated. For this assessment simulations were carried out as described in the preceding paragraph with the drug effect set to zero.

#### Least-Square Mean Analysis

The LS means analysis tested for a statistical significant change in total ADAS-cog score from placebo at 20 months. The model used for the least-square mean analysis was based on change from baseline score as the dependent variable, treatment as the major factor, visit as the repeated factor, and baseline score as a covariate. Furthermore, a treatment-by-visit interaction term was included in the model and subjects nested within treatment served as the grouping factor. A drug effect was considered significant if the 95% confidence interval for the estimated mean group difference did not include the null value (Wald hypothesis test at 5% significance). The LS means analysis was performed in R (version 2.15.2) (18) using the “nlme” package (version 3.1-105) (19).

#### Pharmacometric Total ADAS-Cog Score Analysis

A pharmacometric model was built from a dataset with 400 individuals simulated from the data-generating model. The linear disease progression model described by Ito *et al.* (16)was used as a starting model and modified in a stepwise manner. In this process, the value of each modification was evaluated through goodness of fit plots, residual plots and VPCs. The final model obtained in this manner was refit to additional simulations for 100, 200 and 800 subjects and assessed for its ability to describe general simulations from the longitudinal IRT model. During the CTSs, the model was fitted to each dataset and a drug effect was considered significant if the 95% confidence interval of the drug effect parameter did not include 0 (Wald hypothesis test at 5% significance).

#### Pharmacometric IRT Analysis

The longitudinal IRT model with drug effect, which also served as the simulation model, was fit to all datasets. Similar to the total ADAS-cog score analysis, the exclusion of 0 from the 95% drug effect confidence interval was used a significance criterion (Wald hypothesis test at 5% significance).

Both pharmacometric analyses (total ADAS-cog and IRT) were performed using NONMEM 7.3 beta (12).

## Results

### Assessment of Baseline Cognition from Multiple Data Sources

**Fig. 1:**
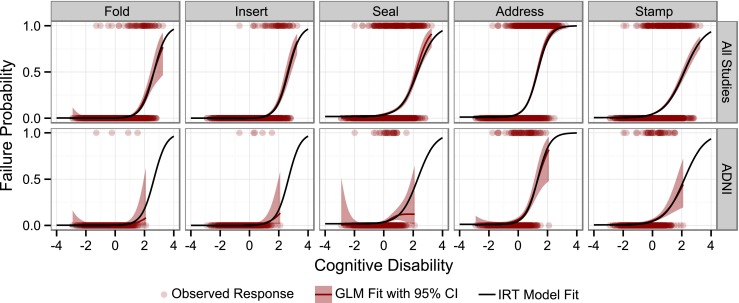
Example of a diagnostic plot for the “ideational praxis” component comparing the IRT model fit (*black line*) to the fit of generalized additive model (GAM) with cross-validated cubic spline as a smoothing function (*dark red line* with 95% confidence interval in *light red*).

**Fig. 2:**
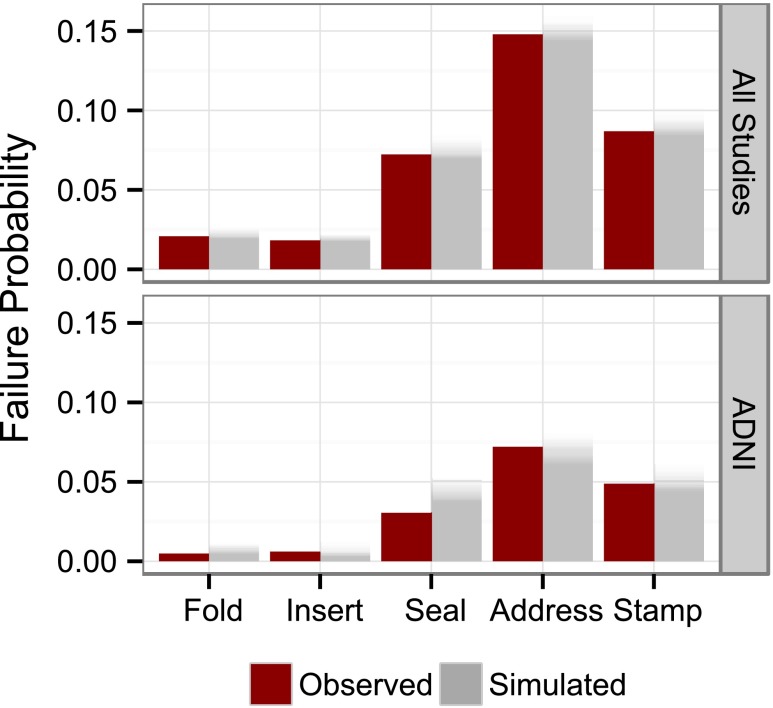
Comparison of observed (*dark red*) and simulated (*grey*) fraction of subjects failing a certain tasks of the “ideational praxis” component as an example of a simulation based diagnostic. The *grey shading* visualizes the variability from the 100 repetitions of the simulations.

All test-specific parameters in the model were successfully estimated using data from all eight studies (final estimates can be found in online Supplementary Material A). Uncertainty information could not be obtained as the covariance step did not complete and runtimes prohibited the use of the bootstrap procedure. Figures 1 and 2 exemplify the goodness of fit and simulation diagnostics performed for all test items and all studies (the remaining diagnostics are available in the online Supplementary Material B). In addition to qualifying the chosen probabilistic model, these diagnostic plots also underline the validity of the different models across studies and allow identification of studies with deviating disability-response relationship. The validity of the normality assumption for *cognitive disability* was evaluated graphically.

**Fig. 3:**
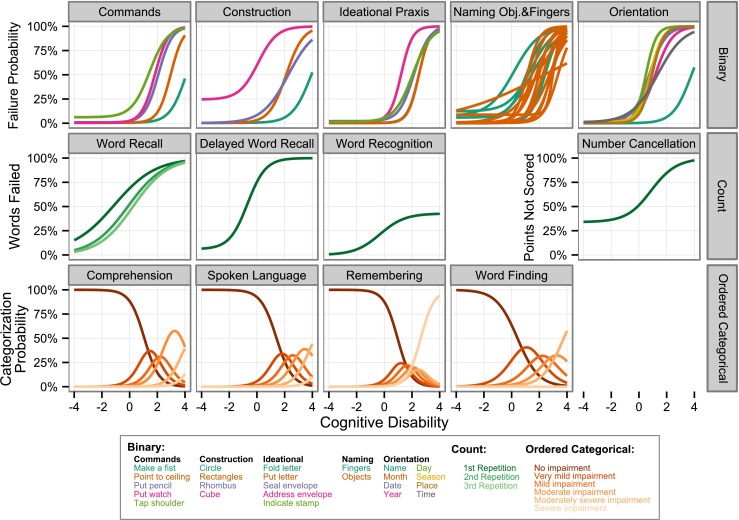
Item characteristic curves (ICCs) for the different items of the ADAS-cog assessment, describing the probability to fail a specific task (*first row*), the percentage of forgotten words (second row, first three panels), the percentage of points not scores in the number cancellation test (second row, last panel) and the probability of being assigned to a certain category (*third row*) for a subject with a given disability. *Cognitive disability* represents a Z-score relative to the estimation data, i.e., a value of 1 indicates a score which is −1 standard deviation lower than the mean of the original data.

The ICCs for the 13 cognitive test components of the ADAS-cog assessment are shown in Fig. 3. For the binary response components (top row), all curves portray the characteristic S-shape in the range of −4 to 4, with a low failure probability for low scores and high failure probability for higher scores. The construction component (top row, second column) is illustrative for understanding the individual ICCs. While the task “Draw a Circle” (green) can even be performed by patients with large disability, the “Draw a Cube” task (pink) represents a challenge for even 20% of healthy elderly subjects. It is important to note that a non-zero intercept can be caused by a number of different reasons unrelated to cognition, i.e., the probability to fail might not depend exclusively on *cognitive disability* but also on other non-considered factors. For example, the non-zero intercept for the task “tap shoulder” could be due to a certain percentage of elderly people with a restricted range of motion. Noteworthy are the ICCs for the three repetitions of the “Word Recall” test. While healthy individuals improve by the second repetition, patients with high *cognitive disability* value show little change between repetitions.

The panels for the ordered categorical type of response show the probability of a certain classification as a function of *cognitive disability*. It is apparent here that Category 0 is the most frequently assigned category in all components. The figure also illustrates that there is considerable overlap between categories, indicating that patients with a specific *cognitive disability* value can be assigned to different categories with similar probability.

### Increasing Trial Efficiency by Selection of a Sensitive Subtest

**Fig. 4:**
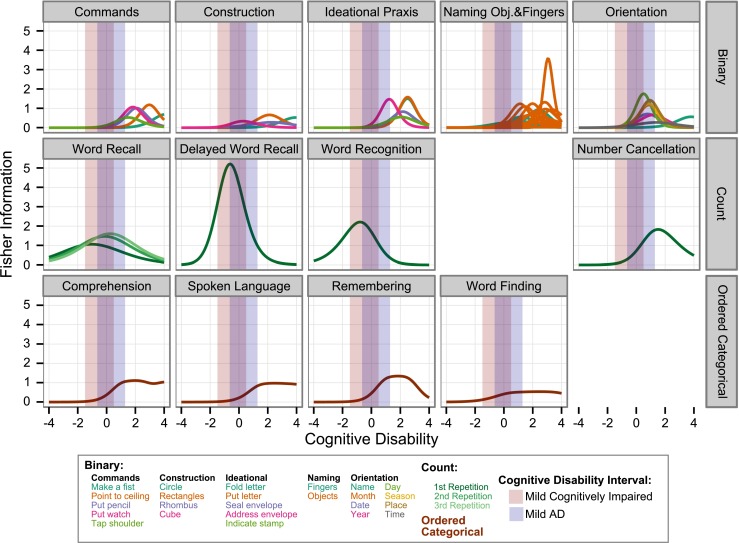
Information content for the different items of the ADAS-cog assessment *versus* cognitive disability. Assessment components are represented as separate panels, each line corresponds to an item. A task with a higher information value will determine a subject’s cognitive disability more precisely. The shaded areas indicate the cognitive disability interval containing 95% of the subject in a mild cognitively impaired (MCI) (*pink*) and a mild AD (*purple*) patient population.

Figure 4 depicts the Fisher information of the different ADAS-cog items as a function of *cognitive disability*. A task with a higher information value will determine a subject’s *cognitive disability* more precisely. For reference, the 95% prediction intervals of cognitive disability in the MCI and in the mild AD population as estimated from the ADNI database are also shown in Fig. 4 (group information is not used in the calculation). The information curves clearly differ both in amplitude as well as in location of the maxima. Most items have their information peak to the right of the 95% *cognitive disability* intervals, indicating a higher sensitivity for patients more severely impaired than those studied in the ADNI study. An exception is the “Delayed Word Recall” test, which has particularly high amplitude and is most informative for relatively low disabilities. Remarkable is also the particularly low information content for the rater assessed items in the bottom row of Fig. 4.

**Fig. 5:**
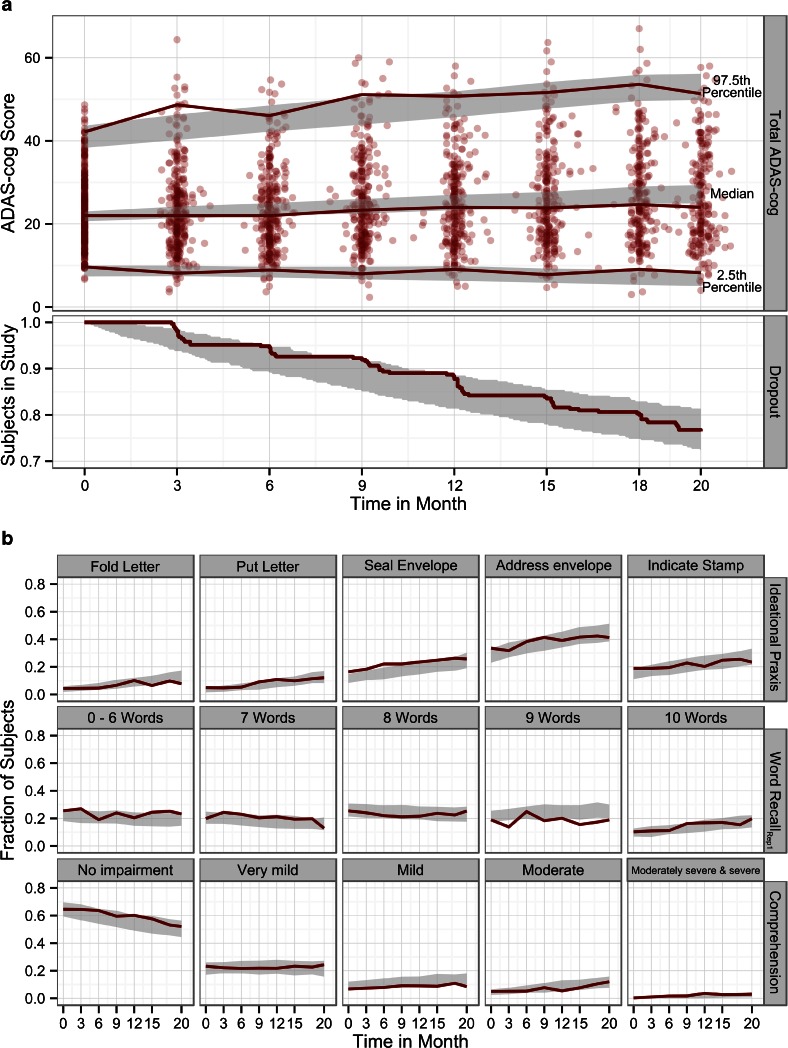
(**a**) Comparisons between observed data and data simulated from the longitudinal IRT model for the total ADAS-cog score and the study dropout. Data derived quantities are dark red and the model simulated 95% confidence interval grey. (**b**) Comparison of observed (*red lines*) and model simulated 95% confidence interval for the fraction of subjects over time that failed a specific task (*first row*), forgot a certain number of words (*second row*) or were assigned to a specific category (*third row*).

A quantitative evaluation of the information content for each item can be obtained by calculating the expected information for the MCI and the mild AD patient populations (Table II). The total information, i.e., the sum over all components, is 25% higher in the mild AD patient population than in the MCI population. Also ranking the individual components by their contribution yields considerably different results. For the MCI population, about 90% of the information is contained in six components: “Delayed Word Recall”, “Word Recall”, “Orientation”, “Word Recognition”, “Naming Objects & Fingers” and “Number Cancellation”. For the mild AD population, information is more evenly distributed between components. While the “Delayed Word Recall” component carries most information in the MCI population, it is only ranked 3rd for mild AD patients and its information content dropped from 4.8 to 3.3. The “Orientation” component, in contrast, has much higher information content in a mild AD population (5.01) than in a MCI population (2.02). These differences are remarkable considering that the two populations have significant overlap (see shaded area in Fig. 5).

**Table II:** Ranking of Test Components by Information Content in a Mild Cognitively Impaired (MCI) and in a Mild AD Patient Population

	MCI population			Mild AD population		
	Component	Information	% Total	Component	Information	% Total
1	Delayed word recall	4.82	30.1%	Orientation	5.01	23.3%
2	Word recall	4.10	25.6%	Word recall	4.16	19.4%
3	Orientation	2.02	12.6%	Delayed word recall	3.34	15.6%
4	Word recognition	1.91	12.0%	Naming objects & fingers	2.86	13.3%
5	Naming objects & fingers	1.10	6.9%	Word recognition	1.38	6.4%
6	Number cancellation	0.40	2.5%	Ideational praxis	0.93	4.3%
7	Construction	0.34	2.1%	Number cancellation	0.87	4.0%
8	Word finding	0.29	1.8%	Construction	0.58	2.7%
9	Remembering	0.25	1.5%	Commands	0.56	2.6%
10	Comprehension	0.21	1.3%	Remembering	0.48	2.2%
11	Ideational praxis	0.20	1.3%	Comprehension	0.44	2.1%
12	Spoken language	0.13	0.8%	Word finding	0.41	1.9%
13	Commands	0.12	0.8%	Spoken language	0.33	1.5%

### Application of IRT to Describe Longitudinal Changes in Cognition

For the typical individual in the LEADe study the final longitudinal IRT model describes a linear increase in *cognitive disability* from a baseline value of 0.95 and at a rate of 0.35 per year. Individual *cognitive disability* trajectories vary according to an additive inter-subject variability model with variability estimates of 0.68 standard deviations (SD) for the baseline and 0.39 SD for the progression rate. Subjects with a higher baseline disability are more likely to progress faster due to a correlation of 0.54 between the baseline and progression random effects. The temporal variations in *cognitive disability* drive the changes in item responses and consequentially in ADAS-cog score through the time-constant ICCs that were estimated from the baseline data. The dropout pattern in this study was found to be described best by a progression rate dependent hazard function and with final estimates of 1.68 for the baseline hazard and 0.65 for the effect of the progression rate on the dropout hazard.

The longitudinal IRT model describes the change in *cognitive disability* over time. *Cognitive disability,* in this case, is unitless and on the Z-score scale. To ease its interpretation, simulations were used to translate the estimates to the more familiar total ADAS-cog scale (the “measurable variable”) and are shown in Fig. 5a. Based on those simulations, the mean baseline ADAS-cog score was predicted to be 22.7 points (95% confidence interval: [21.7; 23.4]) and the mean yearly increase to be 4.3 points for this population (95% confidence interval: [3.7; 4.9]). These values were in good agreement with the results reported in the study report of this trial (22.5 points and 4.1 points per year) (14).

An appreciation of the data description quality for the joint longitudinal IRT model can be gained from Fig. 5a and b displaying various facets of the observed data in comparison with model simulations. In the top panel of Fig. 5a, simulated item responses are summed back to the total score and compared to the observed total ADAS-cog. The observed ADAS-cog scores from all individuals are summarized by the median, the 2.5th and the 97.5th percentile and compared to the corresponding 95% confidence intervals from the model. For both the outer percentiles, observations and model simulations are in very good agreement for most of the time points. Only at the 2nd and 4th visits the observed 97.5th percentile is slightly above the confidence interval from the simulations. The observed median is contained in the 95% confidence interval from the simulations for all but the final visit.

The performance of the dropout model is illustrated in the lower panel of Fig. 5a. Observations and simulations were in good agreement.

The three rows of panels in Fig. 5b contrast model predicted and observed item responses and demonstrate the inherent level of detail available through the longitudinal IRT model. For a selection of three components, each panel shows the fraction of subjects in the study that failed the specified task (row 1), did not recall the specified number of words (row 2) or were put in a certain category (row 3). In most item level comparisons (remaining components available in the electronic Supplementary Material C), the observed fraction and the 95% confidence interval from the simulations corresponded well with each other. Only some items of the “Naming” and “Spoken Language” components were less well predicted.

### Increasing Drug Effect Detection Power

Based on a simulated dataset from the longitudinal IRT model, a pharmacometric total ADAS-cog score model was built (described in the online Supplementary Material D). The final pharmacometric total ADAS-cog score model was compared with a LS-means analysis and the longitudinal IRT model for their ability to detect a drug effect.

**Fig. 6:**
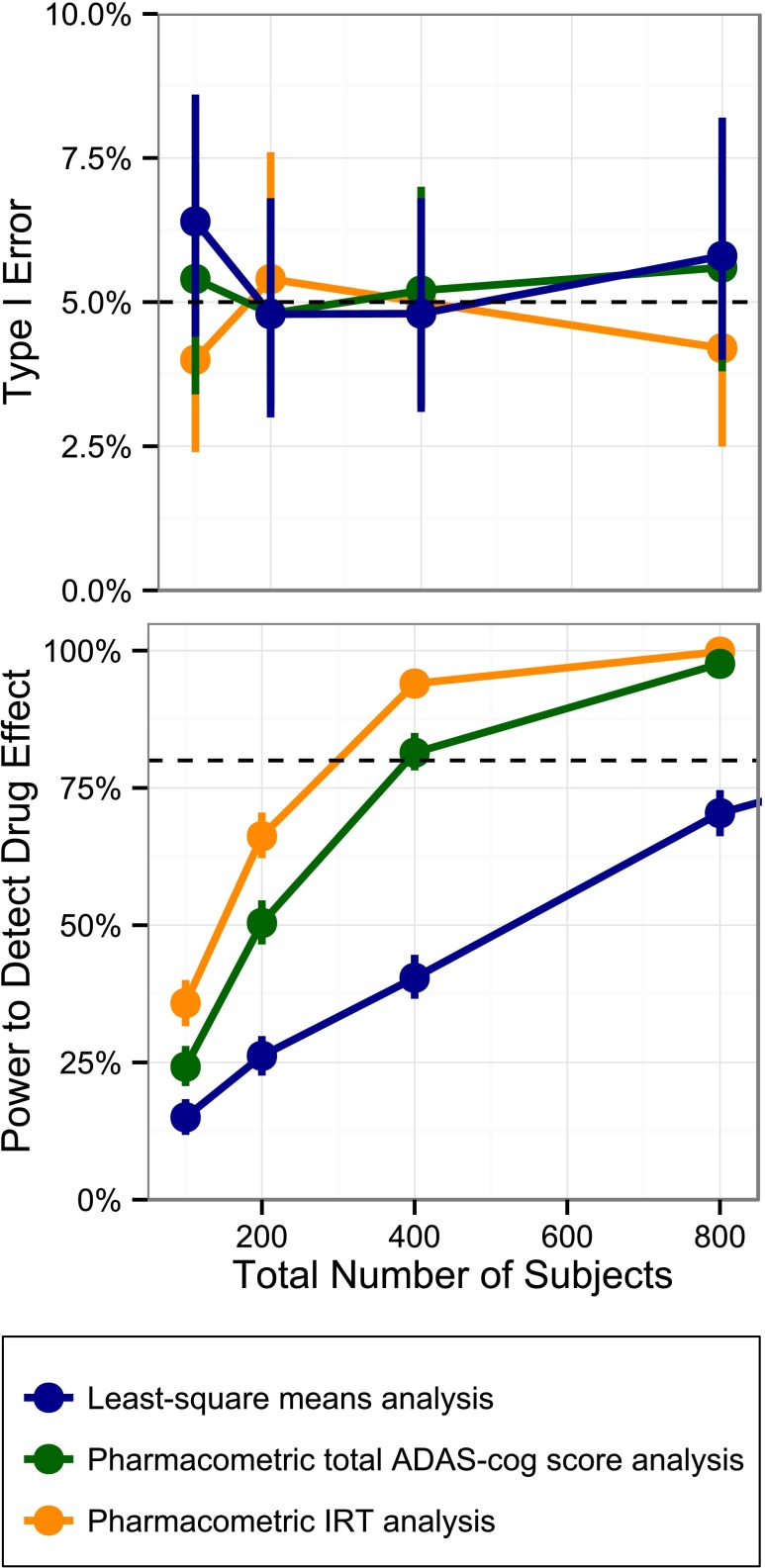
Type I error and power to detect a drug effect *versus* total number of subjects for a least-square means analysis, a pharmacometric total ADAS-cog score analysis and a pharmacometric IRT analysis (*vertical lines* indicate the 95% confidence interval for type I error/power).

The lower panel of Fig. 6 shows the power to detect a drug effect with the three different data analysis methods. Compared to the LS-means analysis both pharmacometric methods provide considerably higher power, with the IRT based method highest amongst the three. More specifically, in order to achieve 80% power the IRT based model requires 71% fewer subjects than the LS-means analysis and 23% fewer subjects than the pharmacometric summary score model.

The upper panel of Fig. 6 displays the type I error under the different methods and compares it to the nominal level of 5%. No significant type I error inflation was observed.

## Discussion

This work explored the potential to improve utilization of cognitive assessment data collected in the ADAS-cog assessment through a combination of IRT and pharmacometric modeling. The potential benefits were studied through four hypotheses focusing on different challenges encountered during the collection and analysis of cognitive data.

One of those challenges is the existence of different ADAS-cog assessment variants due to slight modifications in the set of test components performed in a particular study. Clinical trial databases like CAMD, which collect a diverse set of studies with different patient populations and study protocols, provide a realistic overview of the diversity in ADAS-cog assessments encountered in practice. In this work, four slightly different versions of the ADAS-cog assessment were encountered in the eight studies included in the analysis. Traditionally, a joint analysis of these studies is complicated and requires normalization or a recalculation of the total ADAS-cog score. Alternatively, the IRT methodology interprets each assessment item as a surrogate measure for *cognitive disability* and allows pooling of the complete set of patient responses in a unified analysis. As such, IRT provides an ideal framework for a joint analysis of clinical studies by offering mechanisms to bridge between different ADAS-cog variants, allowing translation of the results from one assessment variant to another. In the same manner, other completely separate cognitive measures could eventually be mapped onto the *cognitive disability* continuum.

Though sufficiently large in size, the baseline dataset (eight studies) mainly consists of mild to moderate AD patients. It would be beneficial to extend this work by including data from more and less severely impaired patients.

As clinical trials move into earlier stages of AD, the identification of the most informative ADAS-cog item subset for a particular patient population has become a common challenge. Usually, these modifications are performed in a heuristic manner. In contrast, a quantitative approach, as presented in this work, provides a more objective method for item selection that agrees well with findings reported by others. The component ranking reported here for the MCI population (Table II) differ only slightly from those identified by Hannesdottir *et al.* with the Pro-ADAS assessment (Unpublished data, presented by Kristin Hannesdottir, Sr. Clinical Research Scientist and Pär Karlsson, Statistical Science Director, on behalf of the Cognition group at AstraZeneca, to the Coalition Against Major Diseases Meeting, April 13th, 2012) , a proposed subset of the ADAS-cog for prodromal AD, selected as being the most sensitive to change over time based on the ADNI dataset. The authors suggest using the following components to evaluate prodromal AD patients: “Word Recall”, “Delayed Word Recall”, “Orientation”, “Word Finding” and “Number Cancellation”. With the calculations done in this work, these components are ranked 1 to 3, 6 and 8. One reason for this divergence might be the extended data source used in this analysis. Another reason is a conceptual difference in the way the different components are modeled and how the information content is calculated. A possible limitation of the approach presented here is the influence of model misspecifications.

The pharmacometric IRT model also proved to be a powerful tool for the description of longitudinal clinical trial data. With a relatively simplistic longitudinal component, the combined model was capable of replicating the observed dynamics both for the total score and on the item level. The approach does not require any transformation to handle the bounded nature of the total score and constitutes a parsimonious description of the raw data. Furthermore, using the previously estimated ICCs provides a simple yet effective way to integrate knowledge available in clinical trial databases into the analysis of a trial. An alternative to fixing the ICC parameter to their previously estimated values is estimating them from the clinical trial data but including an informative prior to represent knowledge from previous trials.

Application of the longitudinal IRT model in a simulation study resulted in a large increase in drug effect detection power from a LS means analysis without inflating the type I error. In the situation of no dropout, this analysis is equivalent to the mixed-effect model repeated measures approach (MMRM). The longitudinal IRT model also significantly outperformed the pharmacometric total ADAS-cog score model, highlighting that the observed increase in power from the classical analysis is not merely a consequence of the use of longitudinal information, but is also a result of the implicit weighing of information occurring in the IRT based analysis.

The results of the power comparison should be interpreted with caution as they favor the IRT model, which is both data generating and one of the analysis models. However, the increased sensitivity of an IRT over a total ADAS-cog score model is in line with the findings of Balsis *et al.* (3) and an increase in power of similar magnitude when using a pharmacometric model instead of classical statistical analysis has been shown by Karlsson *et al.* (20). Furthermore, it should be noted that patient dropout in combination with common data imputation schemes, such as last observation carried forward, will further decrease the probability to detect a drug effect with the LS means analysis, but affect the pharmacometric analyses to a much lesser degree. From this perspective, the comparisons between pharmacometric and traditional analysis shown here could even be considered conservative. The potential savings in the number of individuals becomes even more appealing assuming an average per patient cost of $100 K to $150 K per patient enrolled in an 18 month disease modification trials (including imaging and bio-analytical costs).

Unsatisfactory or unclear results in recent large drug development programs in mild to moderate AD patients, and an increasing focus on early treatment of AD have created a need for better ways to detect changes in cognition for patients early in the disease process. Often, this discussion is focused on defining a fixed sensitive test for the population to be studied. The need to define this test *a priori* is largely driven by the need to have an acceptable test for regulators, where the test characteristics have been well established, or for the need for comparison amongst trials or programs. Such an approach may fail to acknowledge the dynamic and continuous nature of cognitive ability. Even within a group of MCI patients there is tremendous variability in cognitive ability, and the most sensitive assessment might change markedly during the course of a 2–4 year early AD clinical trial (and the subsequent open label extension).

The framework presented in this work is based on a continuous and dynamic understanding of cognition, allows for the use of different existing instruments to assess cognition, and provides a method to choose the most sensitive among the available instruments to utilize the full information available in the assessment data. The IRT framework allows the large body of data from diverse sources and variants of the ADAS-cog already available to be fully utilized and mapped to *cognitive disability*, allowing operating characteristics of any item subsets to be known, in order to meet the regulatory rigors required for qualifying new assessment tools. It also allows present, emerging and future tests to be mapped and compared, to extend the concept of “a common cognitive assessment, using different instruments”. The methodology could easily be expanded to tests that are commonly used in practice that contain cognitive components, such as the mini mental state examination (MMSE), thus linking clinical practice and research measures into one continuous assessment of the patient. The basic concepts described in this work are also directly applicable to measures for other progressive diseases with a clinical endpoint utilizing aggregated scores such as Parkinson’s or rheumatoid arthritis.

## Conclusion

ADAS-cog data can be more effectively and precisely analyzed by combining IRT and pharmacometric modeling. The joint framework permits the combination of assessment variants in a common analysis, the selection of informative test components in a quantitative manner, a closer description of clinical trial data, and, finally, a higher power for identification of drug effects compared to traditional methods. This improved utilization of ADAS-cog score data with IRT and pharmacometric models increases the value of this common assessment and facilitates the integration with novel cognitive outcome measures.

## Electronic supplementary material

Below is the link to the electronic supplementary material.

Supplement A (DOCX 32 kb)

Supplement B (DOCX 7915 kb)

Supplement C (DOCX 1237 kb)

Supplement D (DOCX 1073 kb)

## Abbreviations

3PL: 3 parameter logit
AD: Alzheimer’s disease
ADAS-cog: Alzheimer’s disease assessment scale—cognitive subscale
ADNI: Alzheimer’s disease neuroimaging initiative
CAMD: Coalition Against Major Diseases
C-Path: Critical path institute
CTS: Clinical trial simulations
GAM: Generalized additive model
ICC: Item characteristic curve
IRT: Item response theory
LS: Least-squares
MCI: Mild cognitively impairment
SD: Standard deviation
VPC: Visual predictive check

## Footnotes

1 Data used in preparation of this article were obtained from the Alzheimer’s Disease Neuroimaging Initiative (ADNI) database (adni.loni.ucla.edu). As such, the investigators within the ADNI contributed to the design and implementation of ADNI and/or provided data but did not participate in analysis or writing of this report. A complete listing of ADNI investigators can be found at: http://adni.loni.ucla.edu/wp-content/uploads/how_to_apply/ADNI_Acknowledgement_List.pdf
