# Supplementary material for: Improved Utilization of ADAS-Cog Assessment Data Through Item Response Theory Based Pharmacometric Modeling
Source: Pharm Res. 2014 Mar 5;31(8):2152–65. doi: 10.1007/s11095-014-1315-5 (PMC4153970; doi:10.1007/s11095-014-1315-5)
Supplement: Supplementary file 1 — (DOCX 32 kb) [file 11095_2014_1315_MOESM1_ESM.docx]

Supplement A: IRT Parameter Estimates

The supplement presents all parameter estimates for the baseline IRT model.

**Commands**

| Item j | $\boldsymbol{a}_{\boldsymbol{j}}$ | $\boldsymbol{b}_{\boldsymbol{j}}$ | $\boldsymbol{c}_{\boldsymbol{j}}$ |
| --- | --- | --- | --- |
| 1 | 1.34 | 4.68 | 0.0 |
| 2 | 2.24 | 3.06 | 0.0 |
| 3 | 1.92 | 2.22 | 0.01 |
| 4 | 2.07 | 1.96 | 0.01 |
| 5 | 1.55 | 1.59 | 0.08 |

**Construction**

| Item j | $\boldsymbol{a}_{\boldsymbol{j}}$ | $\boldsymbol{b}_{\boldsymbol{j}}$ | $\boldsymbol{c}_{\boldsymbol{j}}$ |
| --- | --- | --- | --- |
| 1 | 1.2 | 4.4 | 0.0 |
| 2 | 1.65 | 2.22 | 0.0 |
| 3 | 1.1 | 2.35 | 0.0 |
| 4 | 1.41 | 0.17 | 0.21 |

**Ideational Praxis**

| Item j | $\boldsymbol{a}_{\boldsymbol{j}}$ | $\boldsymbol{b}_{\boldsymbol{j}}$ | $\boldsymbol{c}_{\boldsymbol{j}}$ |
| --- | --- | --- | --- |
| 1 | 2.39 | 2.64 | 0.0 |
| 2 | 2.44 | 2.65 | 0.0 |
| 3 | 1.7 | 2.32 | 0.02 |
| 4 | 2.44 | 1.34 | 0.01 |
| 5 | 1.46 | 2.18 | 0.01 |

**Naming Objects & Fingers**

| Item j | $\boldsymbol{a}_{\boldsymbol{j}}$ | $\boldsymbol{b}_{\boldsymbol{j}}$ | $\boldsymbol{c}_{\boldsymbol{j}}$ |
| --- | --- | --- | --- |
| 1 | 1.81 | 2.56 | 0.0 |
| 2 | 1.59 | 1.26 | 0.05 |
| 3 | 1.04 | 0.23 | 0.03 |
| 4 | 1.05 | 1.58 | 0.0 |
| 5 | 1.2 | 1.52 | 0.12 |
| 6 | 1.94 | 3.49 | 0.0 |
| 7 | 2.31 | 2.94 | 0.0 |
| 8 | 3.35 | 3.13 | 0.07 |
| 9 | 1.9 | 2.37 | 0.0 |
| 10 | 1.62 | 1.92 | 0.02 |
| 11 | 1.43 | 2.83 | 0.01 |
| 12 | 2.31 | 2.1 | 0.0 |
| 13 | 1.99 | 2.98 | 0.0 |
| 14 | 2.21 | 1.58 | 0.0 |
| 15 | 2.25 | 1.25 | 0.0 |
| 16 | 0.86 | 2.06 | 0.01 |
| 17 | 0.48 | 2.06 | 0.0 |

**Orientation**

| Item j | $\boldsymbol{a}_{\boldsymbol{j}}$ | $\boldsymbol{b}_{\boldsymbol{j}}$ | $\boldsymbol{c}_{\boldsymbol{j}}$ |
| --- | --- | --- | --- |
| 1 | 1.55 | 3.89 | 0.0 |
| 2 | 2.12 | 0.99 | 0.0 |
| 3 | 1.84 | 0.9 | 0.01 |
| 4 | 1.62 | 1.32 | 0.0 |
| 5 | 2.56 | 0.61 | 0.01 |
| 6 | 2.3 | 0.97 | 0.01 |
| 7 | 2.55 | 1.07 | 0.0 |
| 8 | 1.18 | 1.45 | 0.01 |

**Word Recall**

| Repetition j | $\boldsymbol{a}_{\boldsymbol{j}}$ | $\boldsymbol{b}_{\boldsymbol{j}}$ | $\boldsymbol{c}_{\boldsymbol{j}}$ |
| --- | --- | --- | --- |
| 1 | 0.73 | -0.63 | 0.13 |
| 2 | 0.79 | 0.03 | 0.0 |
| 3 | 0.85 | 0.4 | 0.0 |

**Delayed Word Recall**

| $\boldsymbol{a}_{\boldsymbol{j}}$ | $\boldsymbol{b}_{\boldsymbol{j}}$ | $\boldsymbol{c}_{\boldsymbol{j}}$ |
| --- | --- | --- |
| 1.56 | -0.54 | 0.05 |

**Word Recognition**

| Studies | $\boldsymbol{a}_{\boldsymbol{j}}$ | $\boldsymbol{b}_{\boldsymbol{j}}$ | $\boldsymbol{c}_{\boldsymbol{j}}$ | $\boldsymbol{d}_{\boldsymbol{j}}$ |
| --- | --- | --- | --- | --- |
| CAMD 1137, CAMD 1138 | 2.84 | 0.79 | 0.0 | 0.43 |
| ADNI, CAMD 1137, CAMD 1138, CAMD 1141, CAMD 1142 | 1.08 | -0.06 |  |  |

**Number Cancellation**

| $\boldsymbol{a}_{\boldsymbol{j}}$ | $\boldsymbol{b}_{\boldsymbol{j}}$ | $\boldsymbol{d}_{\boldsymbol{j}}$ | $\boldsymbol{\delta}$ |
| --- | --- | --- | --- |
| 0.98 | 0.87 | 16.4 | 0.44 |

**Comprehension**

| $\boldsymbol{a}_{\boldsymbol{j}}$ | Category k | $\boldsymbol{b}_{\boldsymbol{j,k}}$ |
| --- | --- | --- |
| 1.54 | >1 | 1.17 |
|  | >2 | 1.04 |
|  | >3 | 0.8 |
|  | >4 | 1.58 |
|  | =5 | 1.38 |

**Spoken Language**

| $\boldsymbol{a}_{\boldsymbol{j}}$ | Category k | $\boldsymbol{b}_{\boldsymbol{j,k}}$ |
| --- | --- | --- |
| 1.51 | >1 | 1.03 |
|  | >2 | 0.7 |
|  | >3 | 0.6 |
|  | >4 | 0.51 |
|  | =5 | 0.44 |

**Remembering**

| $\boldsymbol{a}_{\boldsymbol{j}}$ | Category k | $\boldsymbol{b}_{\boldsymbol{j,k}}$ |
| --- | --- | --- |
| 1.6 | >1 | 1.57 |
|  | >2 | 0.9 |
|  | >3 | 0.85 |
|  | >4 | 1.01 |
|  | =5 | 1.46 |

**Word Finding**

| $\boldsymbol{a}_{\boldsymbol{j}}$ | Category k | $\boldsymbol{b}_{\boldsymbol{j,k}}$ |
| --- | --- | --- |
| 1.25 | >1 | 0.54 |
|  | >2 | 1.4 |
|  | >3 | 1.05 |
|  | >4 | 1.05 |
|  | =5 | 179.34 |
