# Supplementary material for: Improved Utilization of ADAS-Cog Assessment Data Through Item Response Theory Based Pharmacometric Modeling
Source: Pharm Res. 2014 Mar 5;31(8):2152–65. doi: 10.1007/s11095-014-1315-5 (PMC4153970; doi:10.1007/s11095-014-1315-5)
Supplement: Supplementary file 2 — (DOCX 7915 kb) [file 11095_2014_1315_MOESM2_ESM.docx]

Supplement B: IRT Diagnostics

The supplement shows diagnostic plots for the performance of the IRT model in all 8 studies and for all items of the ADAS-cog assessment.

**Commands**


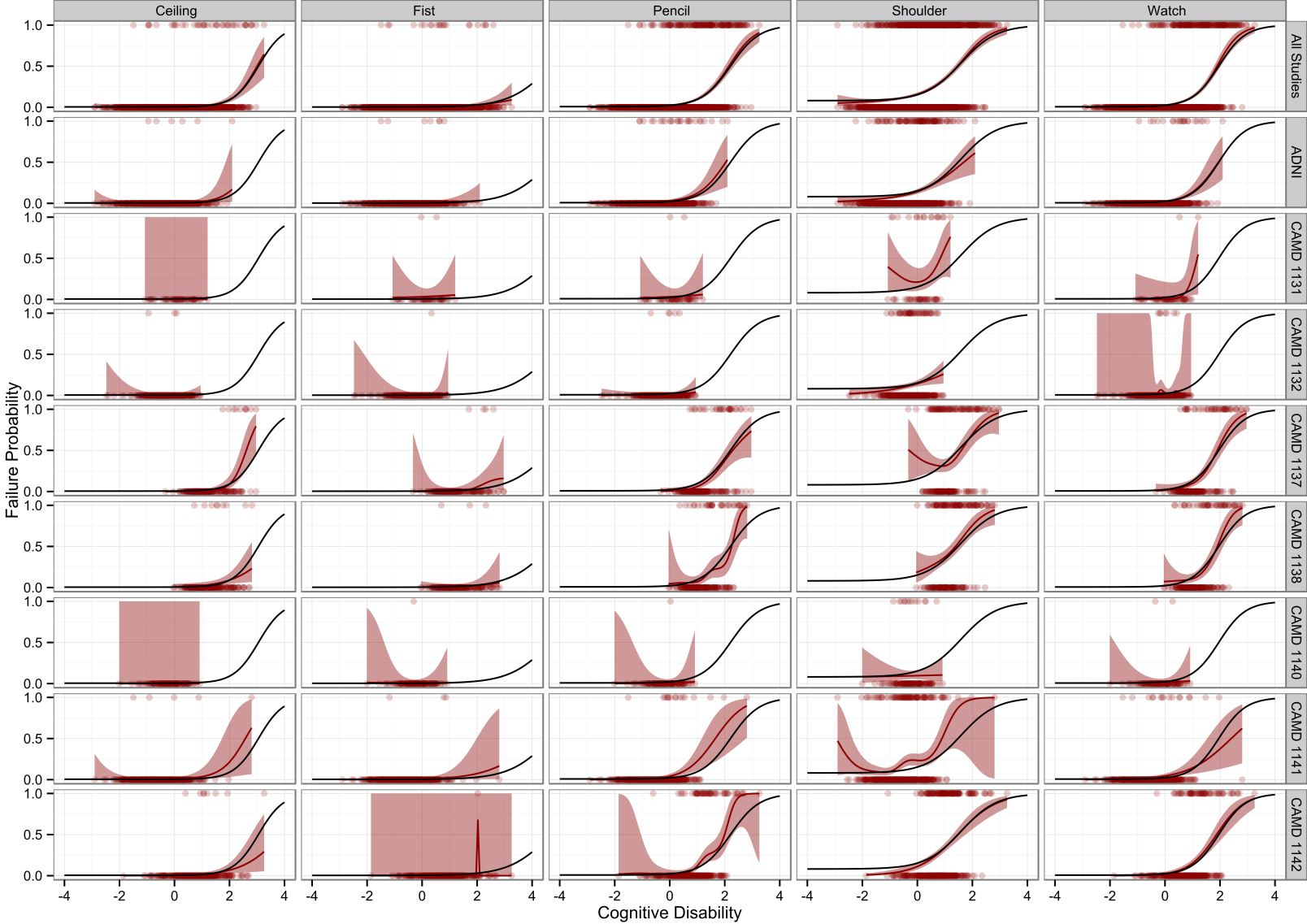


Figure B.1: ICC curves from the IRT model fit (black line) compared to the fit of a generalized additive model (GAM ) with cross-validated cubic spline as a smoothing function (dark red line with 95% confidence interval in light red). Red dots are the observed scores.


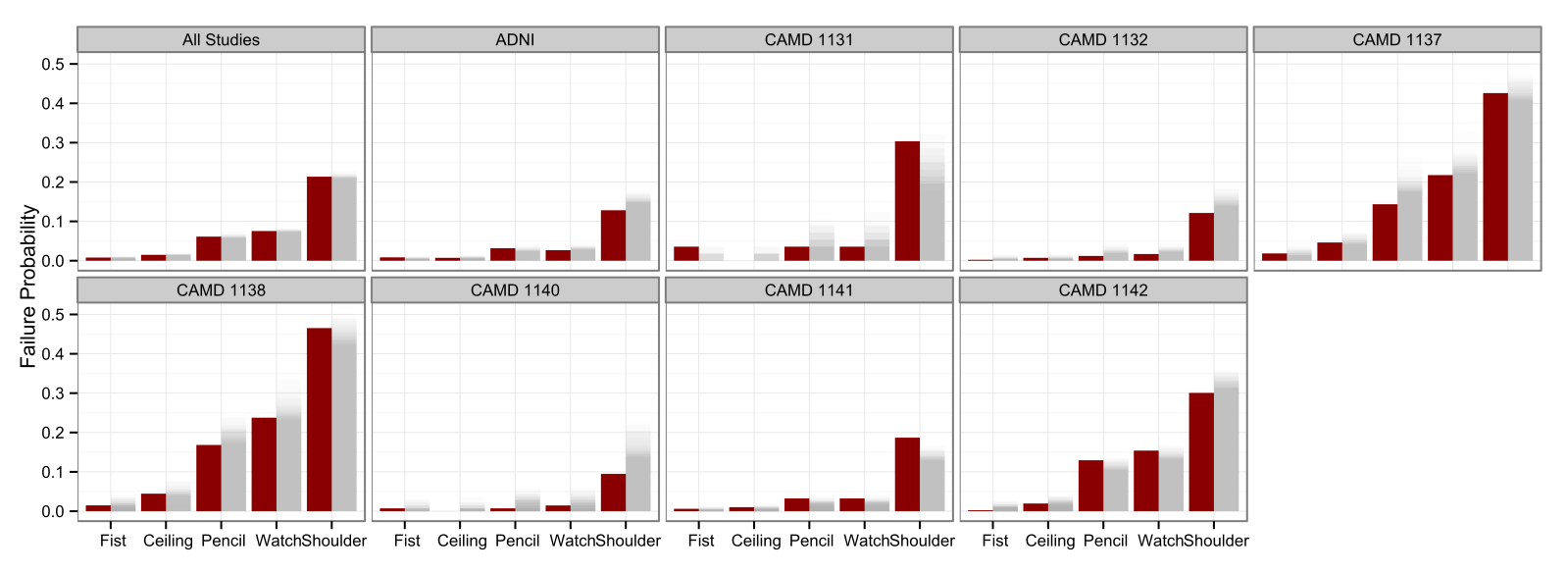
Figure B.2: Observed (dark red) and simulated (grey) fraction of subjects failing a certain tasks of the command component. The grey shading visualizes the variability from 100 repetitions of the simulations.

**Construction**


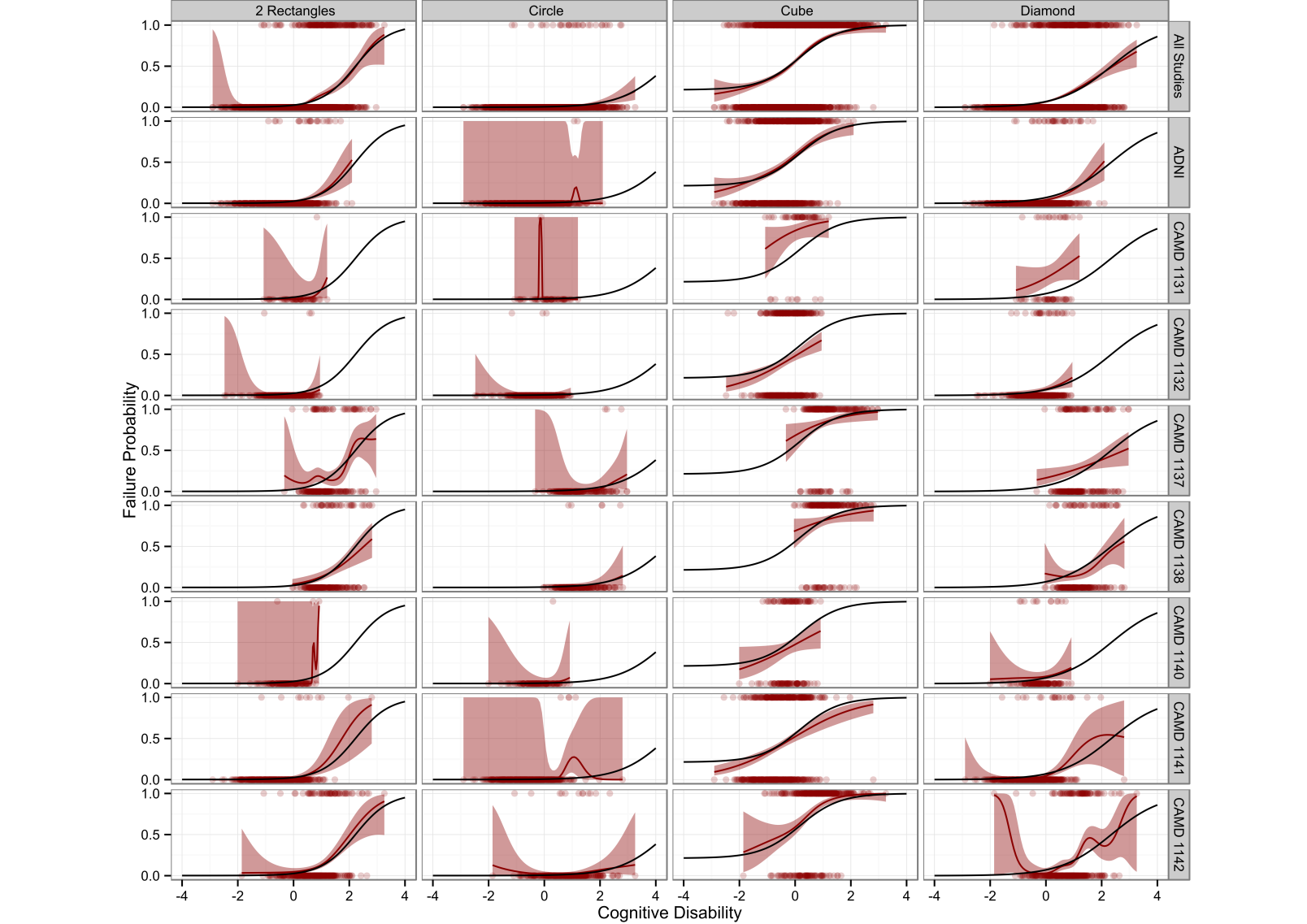


Figure B.3: ICC curves from the IRT model fit (black line) compared to the fit of a generalized additive model (GAM ) with cross-validated cubic spline as a smoothing function (dark red line with 95% confidence interval in light red). Red dots are the observed scores.
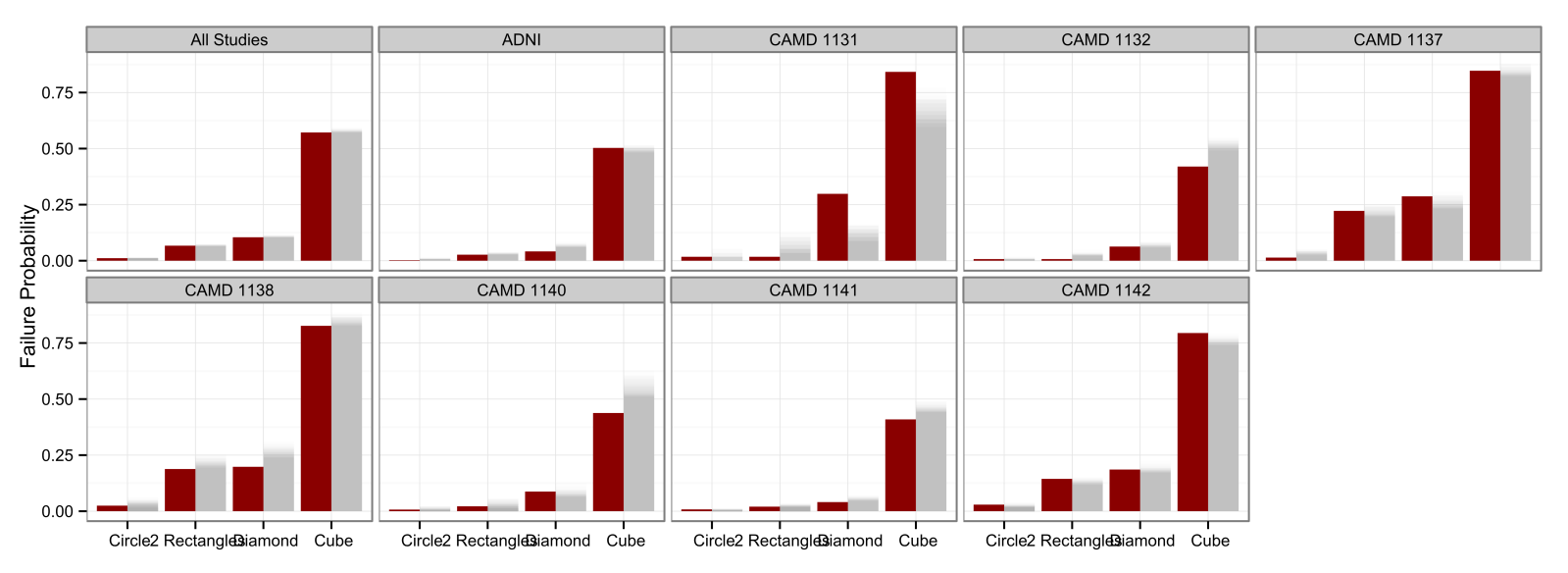
 Figure B.4: Observed (dark red) and simulated (grey) fraction of subjects failing a certain tasks of the construction component. The grey shading visualizes the variability from 100 repetitions of the simulations.

**Ideational Praxis**


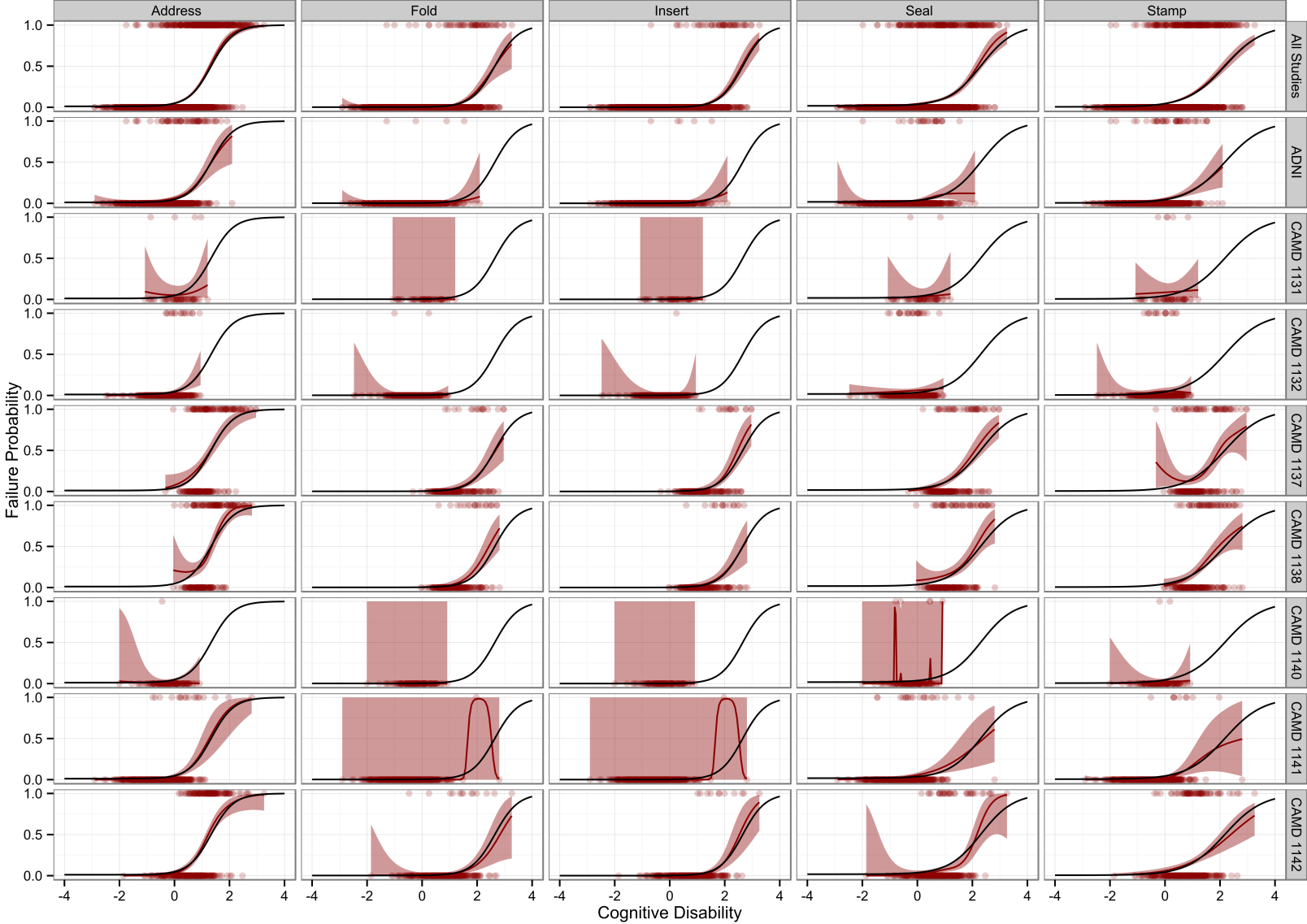


Figure B.5: ICC curves from the IRT model fit (black line) compared to the fit of a generalized additive model (GAM ) with cross-validated cubic spline as a smoothing function (dark red line with 95% confidence interval in light red). Red dots are the observed scores.


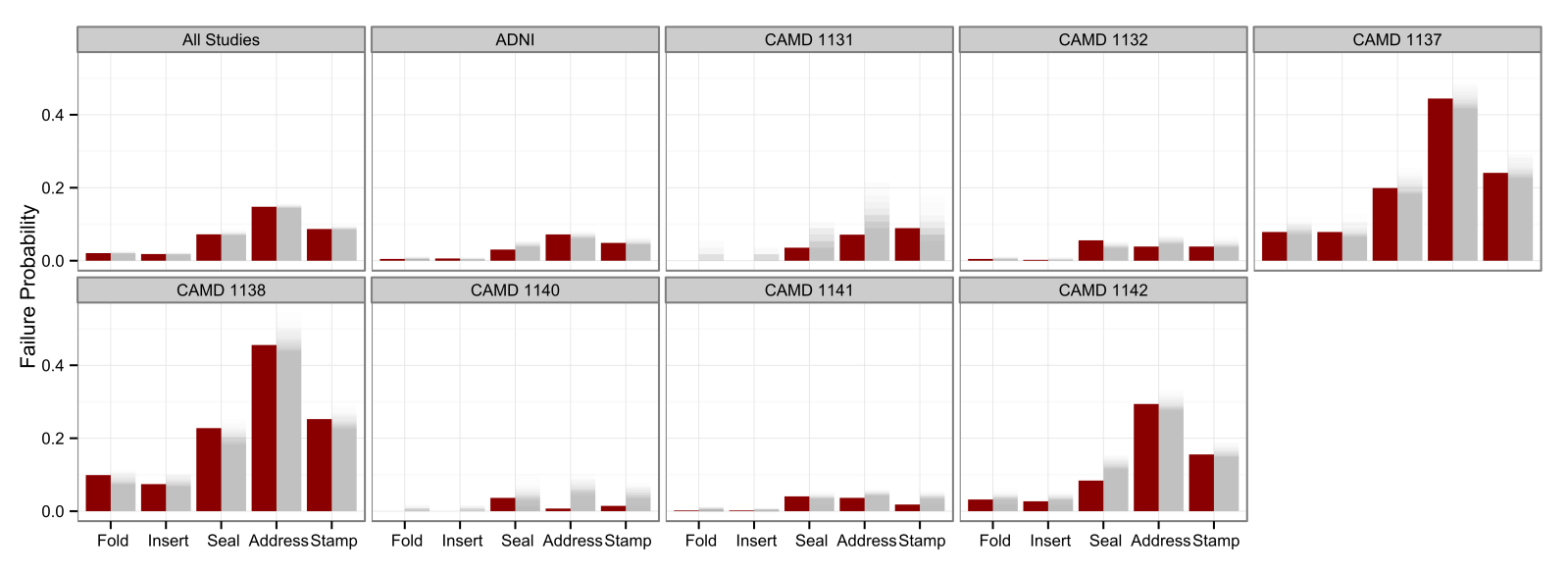


Figure B.6: Observed (dark red) and simulated (grey) fraction of subjects failing a certain tasks of the ideational praxis component. The grey shading visualizes the variability from 100 repetitions of the simulations.

**Naming Objects & Fingers**


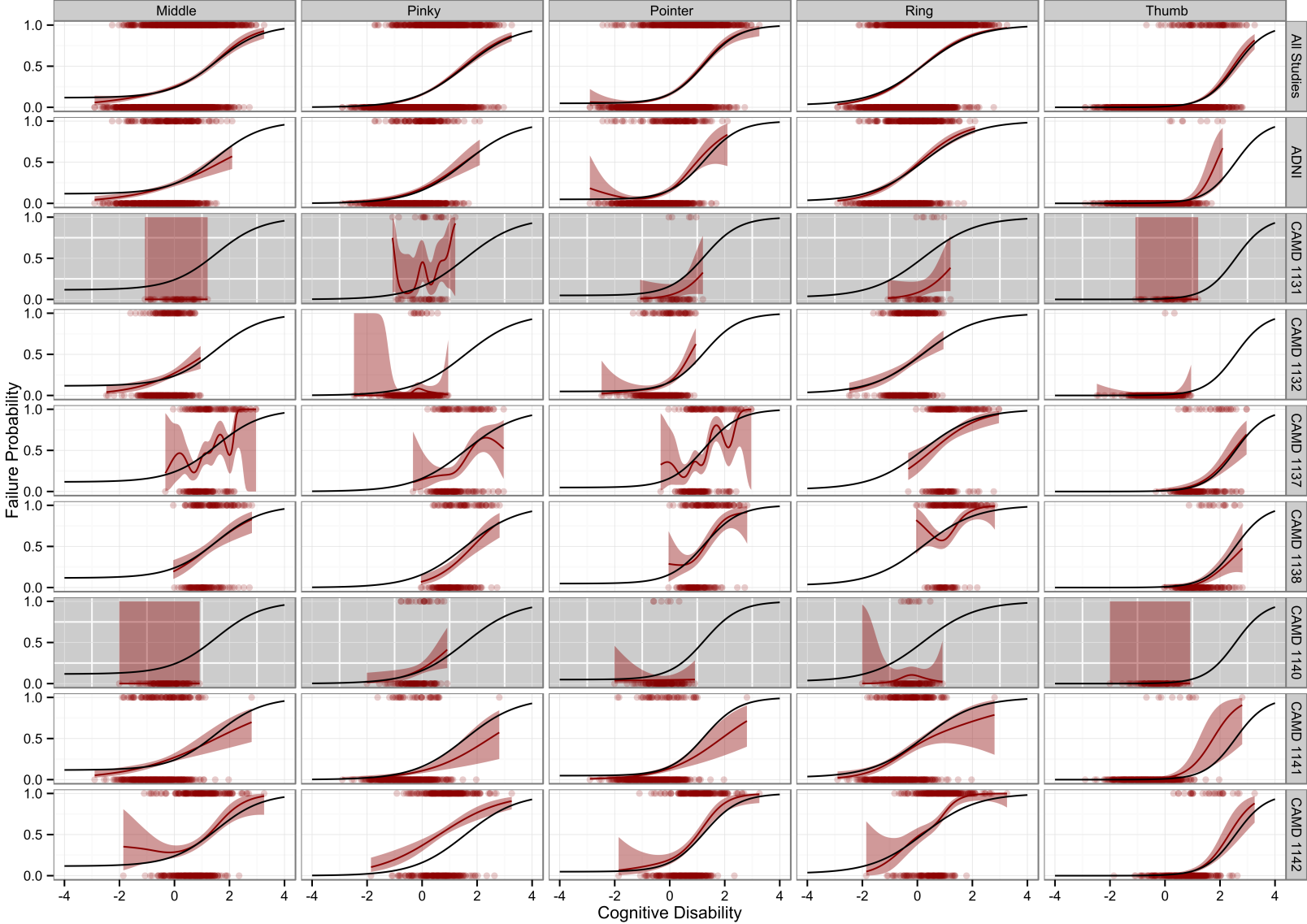


Figure B.7: ICC curves from the IRT model fit (black line) compared to the fit of a generalized additive model (GAM ) with cross-validated cubic spline as a smoothing function (dark red line with 95% confidence interval in light red). Red dots are the observed scores. Panels with grey background visualize data that was excluded from the analysis.


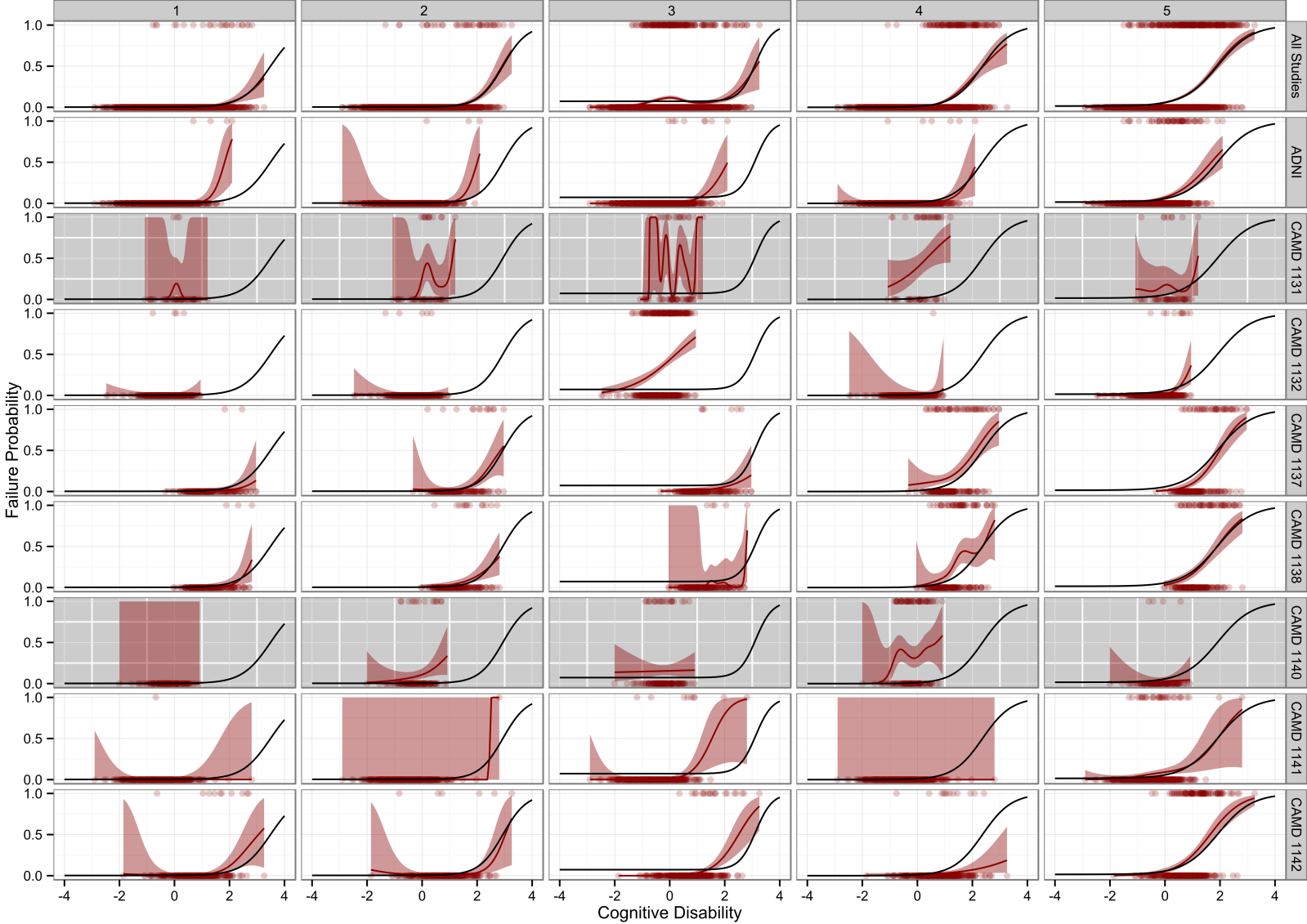


Figure B.8: ICC curves from the IRT model fit (black line) compared to the fit of a generalized additive model (GAM ) with cross-validated cubic spline as a smoothing function (dark red line with 95% confidence interval in light red). Red dots are the observed scores. Panels with grey background visualize data that was excluded from the analysis.


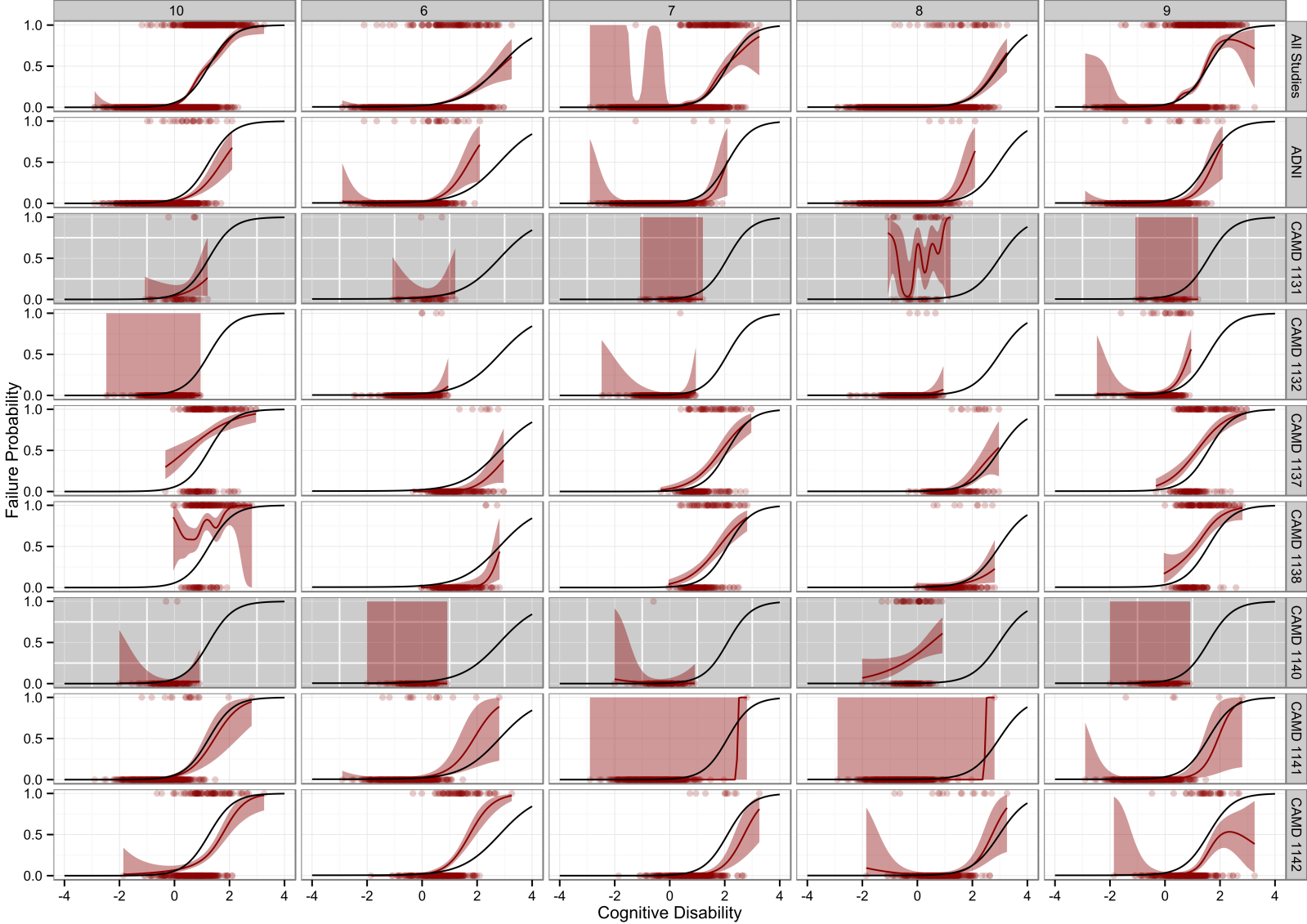


Figure B.9: ICC curves from the IRT model fit (black line) compared to the fit of a generalized additive model (GAM ) with cross-validated cubic spline as a smoothing function (dark red line with 95% confidence interval in light red). Red dots are the observed scores. Panels with grey background visualize data that was excluded from the analysis.


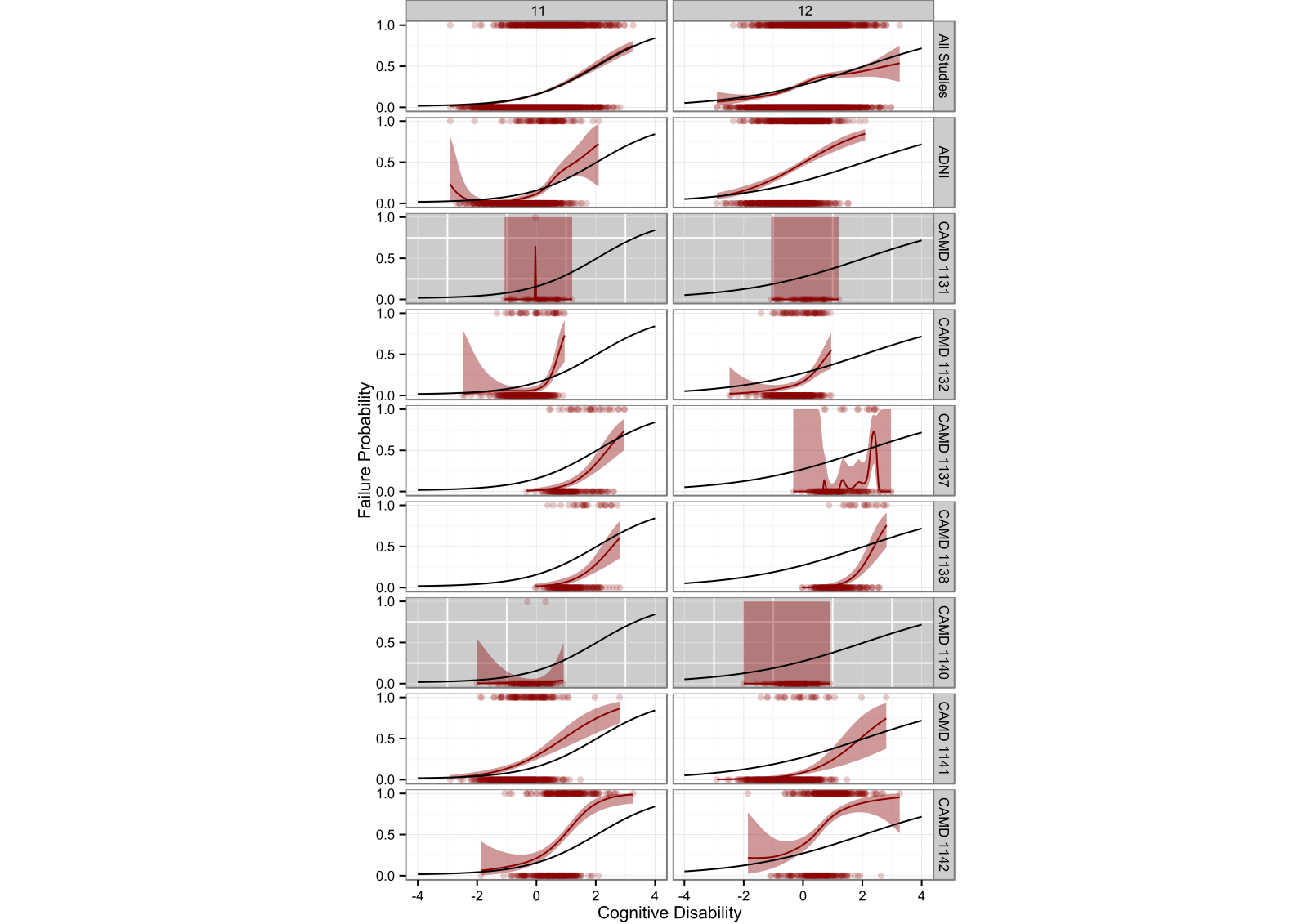


Figure B.10: ICC curves from the IRT model fit (black line) compared to the fit of a generalized additive model (GAM ) with cross-validated cubic spline as a smoothing function (dark red line with 95% confidence interval in light red). Red dots are the observed scores. Panels with grey background visualize data that was excluded from the analysis.


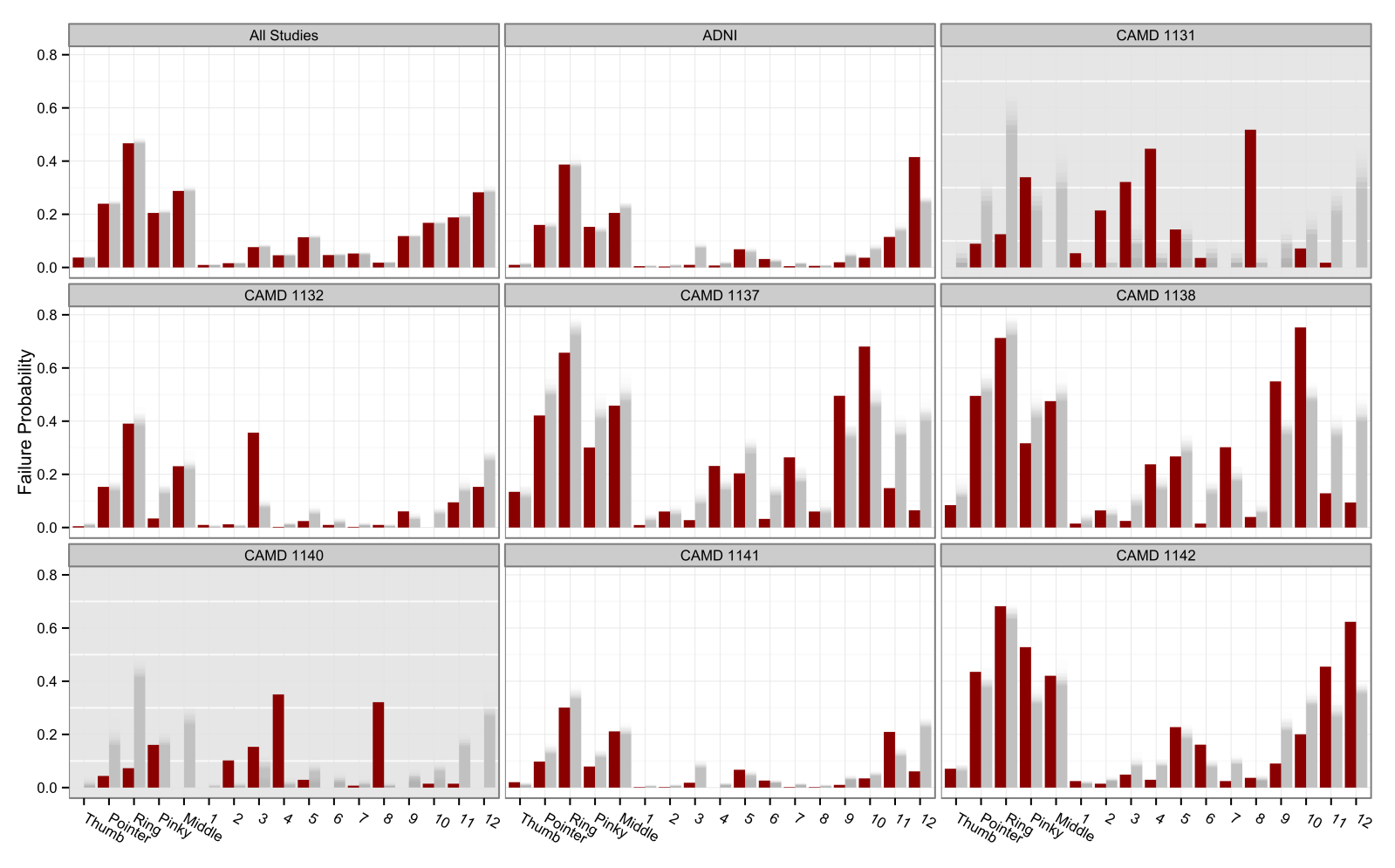


Figure B.11: Observed (dark red) and simulated (grey) fraction of subjects failing a certain tasks of the naming objects & fingers component. The grey shading visualizes the variability from 100 repetitions of the simulations. Panels with grey background visualize data that was excluded from the analysis.

**Orientation**


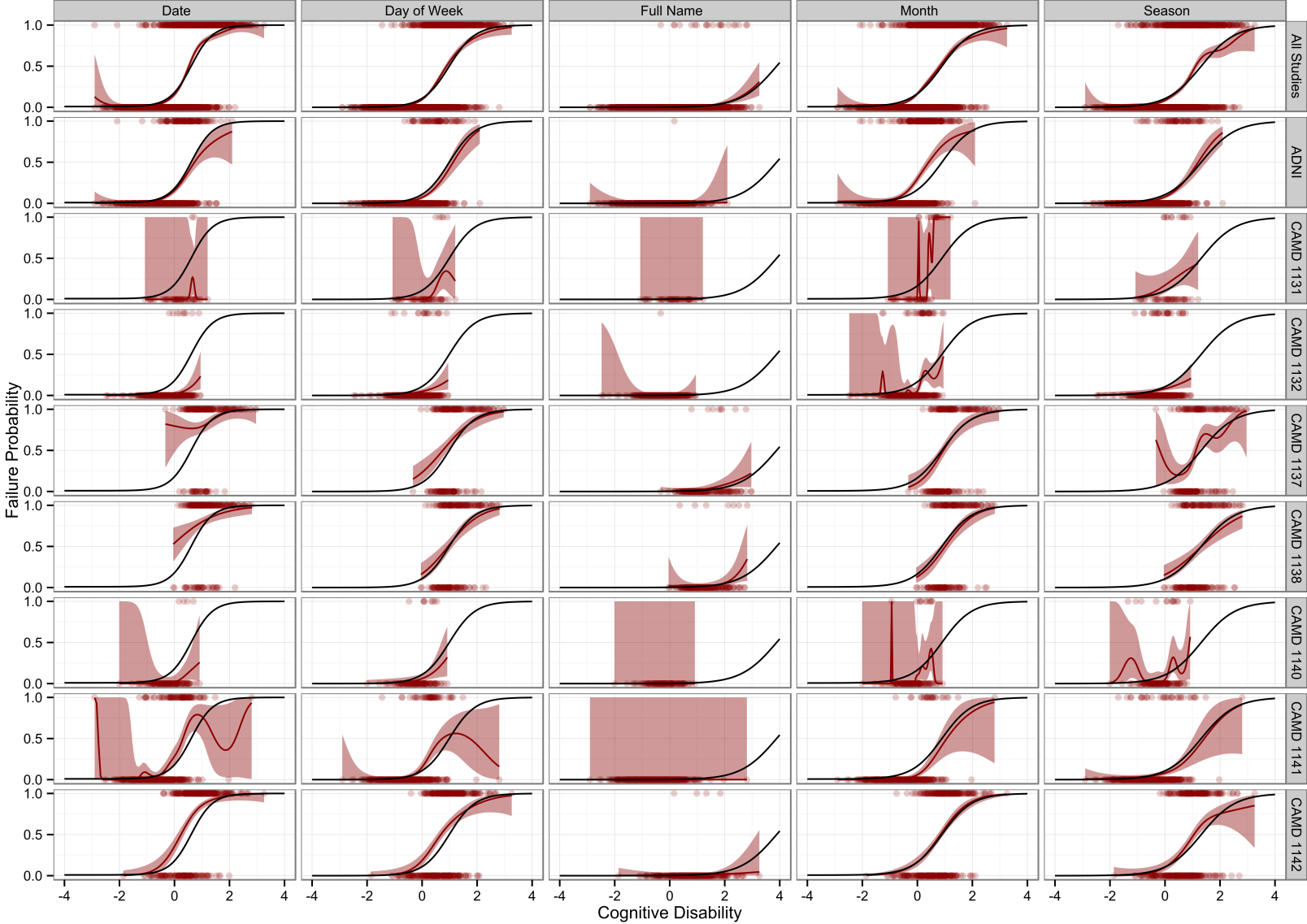


Figure B.12: ICC curves from the IRT model fit (black line) compared to the fit of a generalized additive model (GAM ) with cross-validated cubic spline as a smoothing function (dark red line with 95% confidence interval in light red). Red dots are the observed scores.


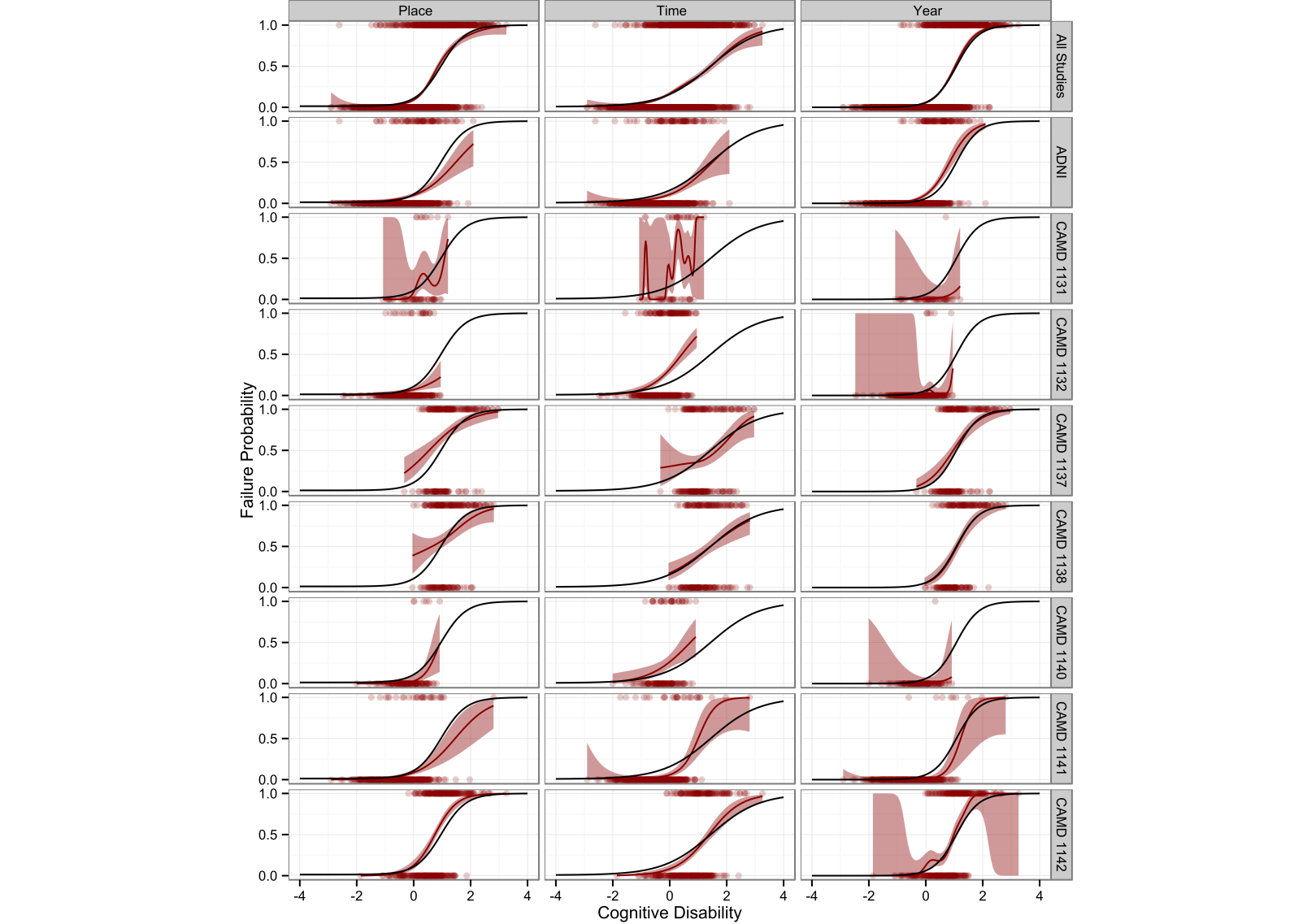


Figure B.13: ICC curves from the IRT model fit (black line) compared to the fit of a generalized additive model (GAM ) with cross-validated cubic spline as a smoothing function (dark red line with 95% confidence interval in light red). Red dots are the observed scores.


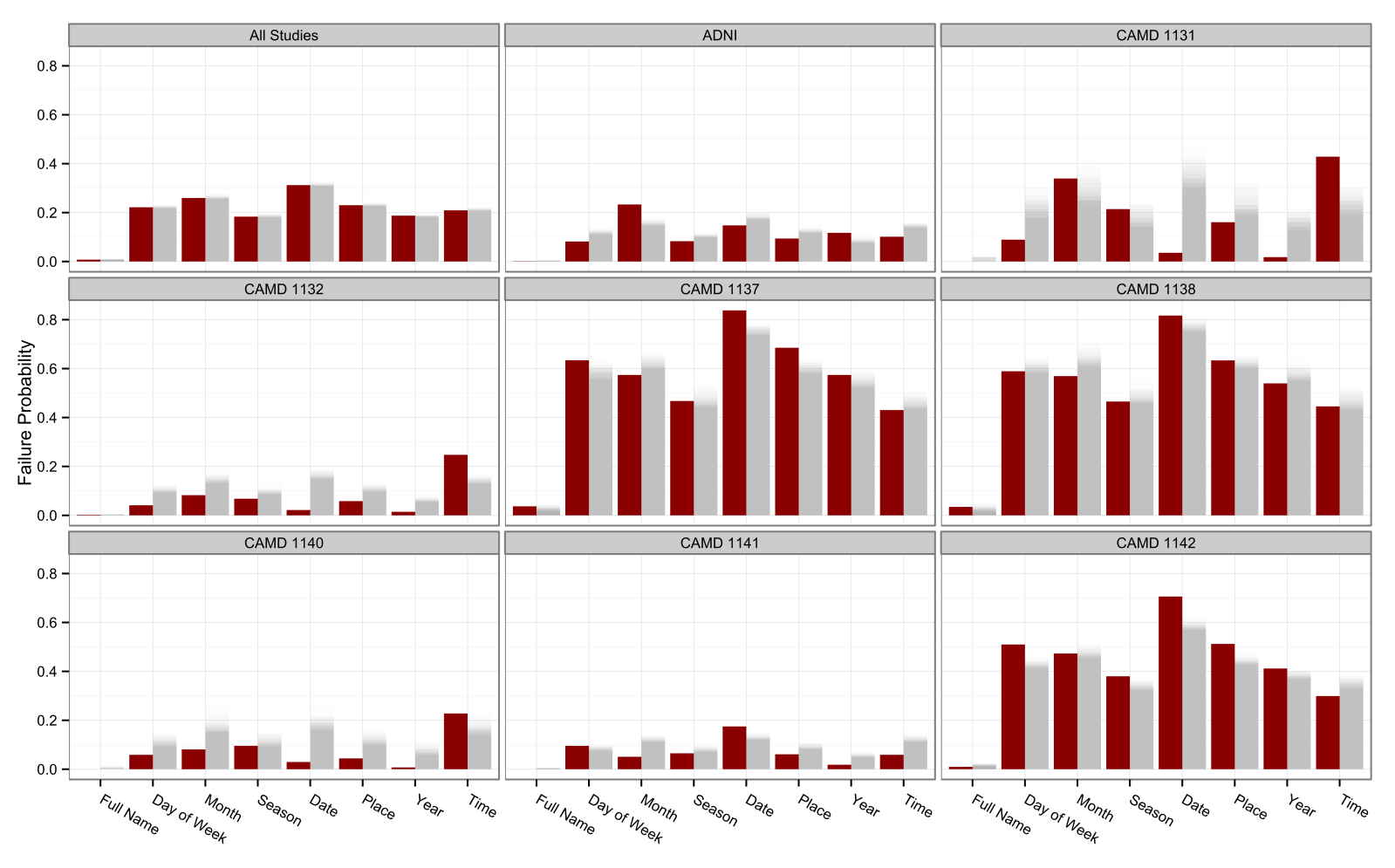


Figure B.14: Observed (dark red) and simulated (grey) fraction of subjects failing a certain tasks of the orientation component. The grey shading visualizes the variability from 100 repetitions of the simulations.

**Word Recall**


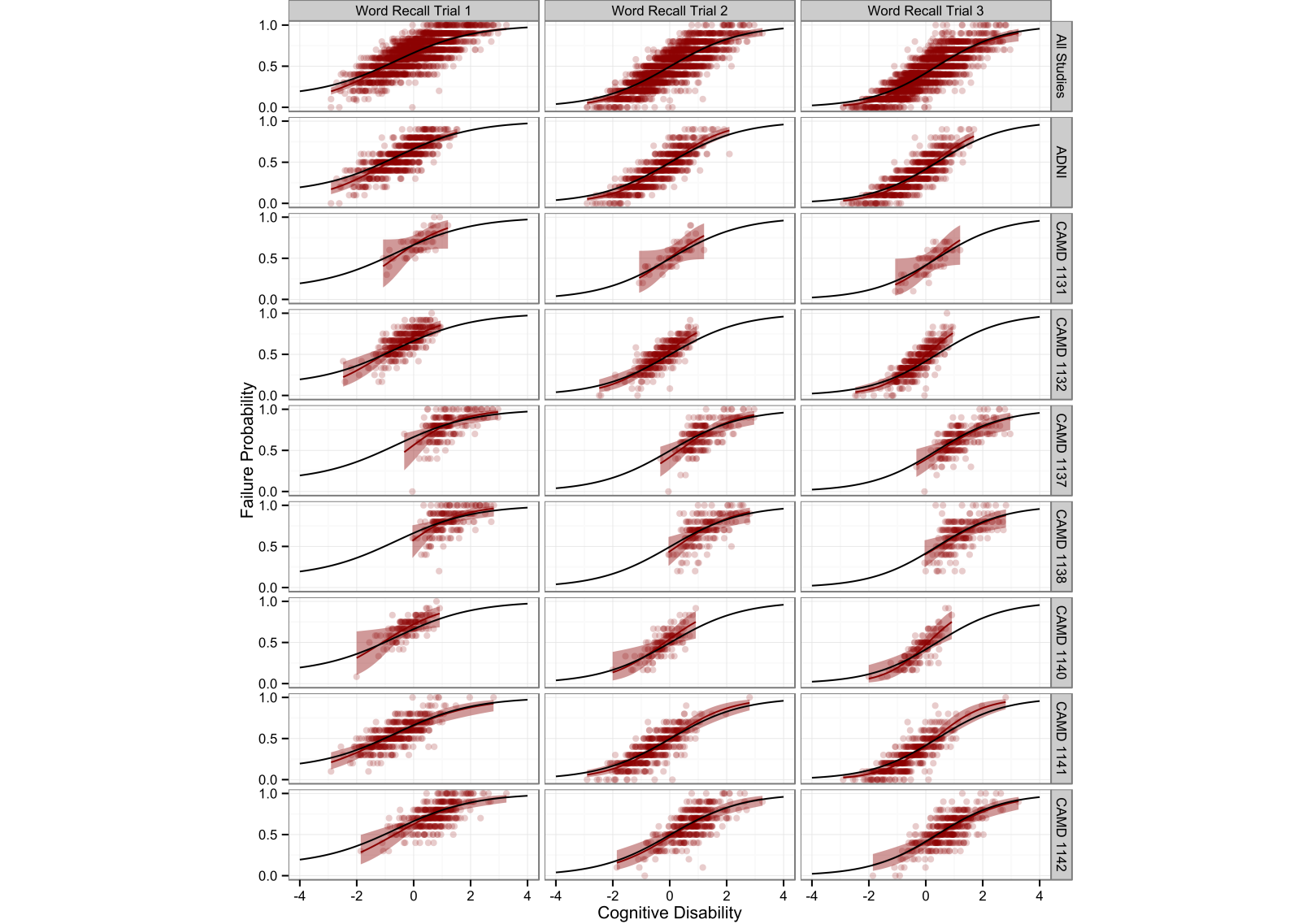


Figure B.15: ICC curves from the IRT model fit (black line) compared to the fit of a generalized additive model (GAM ) with cross-validated cubic spline as a smoothing function (dark red line with 95% confidence interval in light red). Red dots are the observed scores.

**Repetition 1**


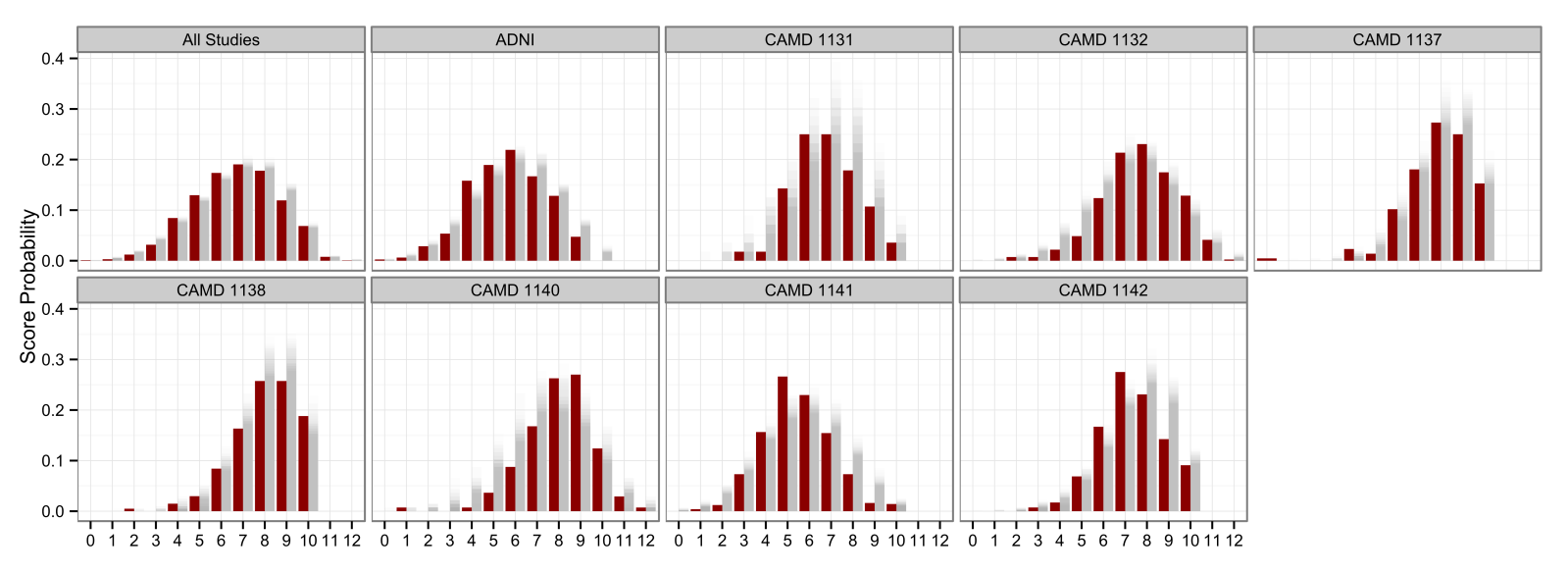
Figure B.16: Observed (dark red) and simulated (grey) distribution of scores for 1^st^ repetition of the word recall component. The grey shading visualizes the variability from 100 repetitions of the simulations.

**Repetition 2**


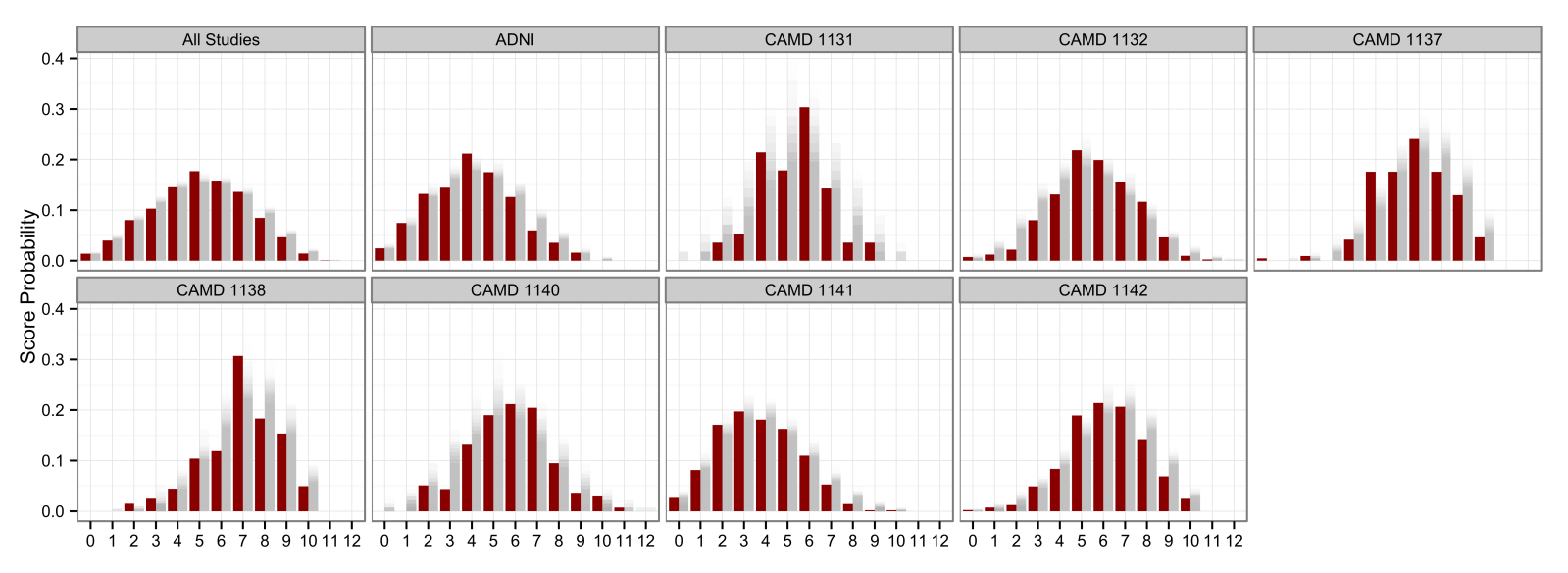
 Figure B.17: Observed (dark red) and simulated (grey) distribution of scores for 2^nd^ repetition of the word recall component. The grey shading visualizes the variability from 100 repetitions of the simulations.

**Repetition 3**


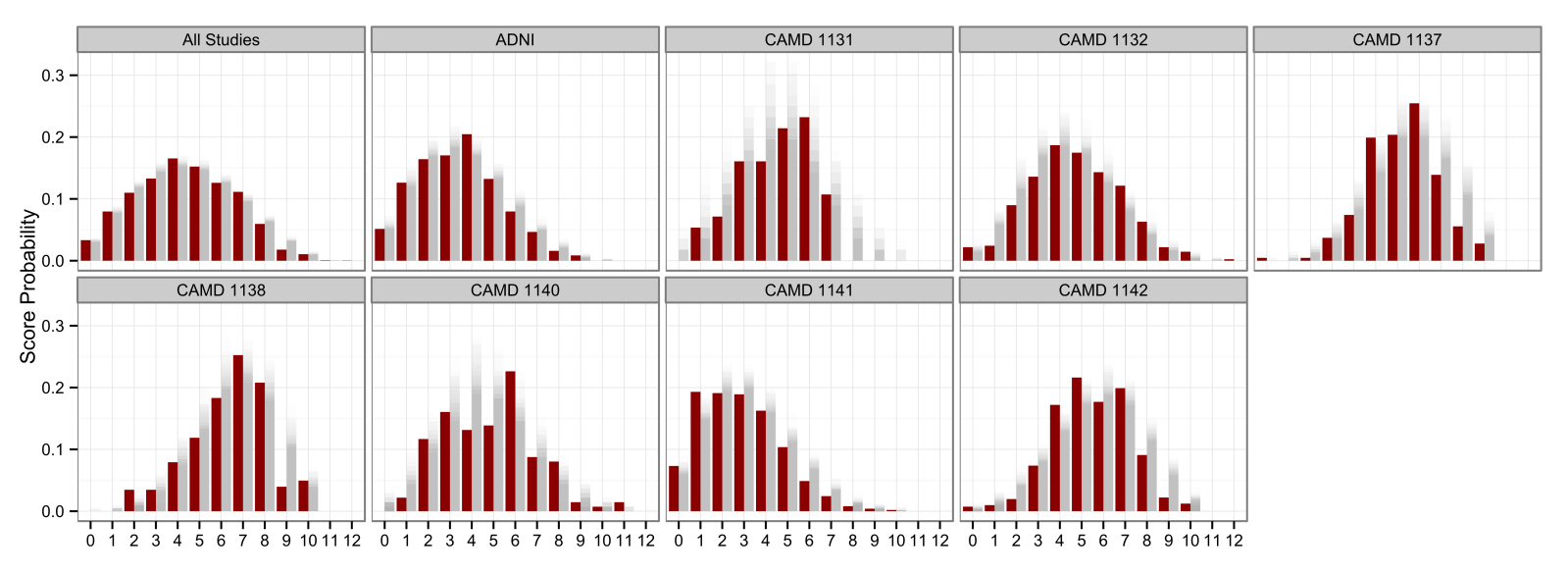
 Figure B.18: Observed (dark red) and simulated (grey) distribution of scores for 3^rd^ repetition of the word recall component. The grey shading visualizes the variability from 100 repetitions of the simulations.

**Delayed Word Recall**


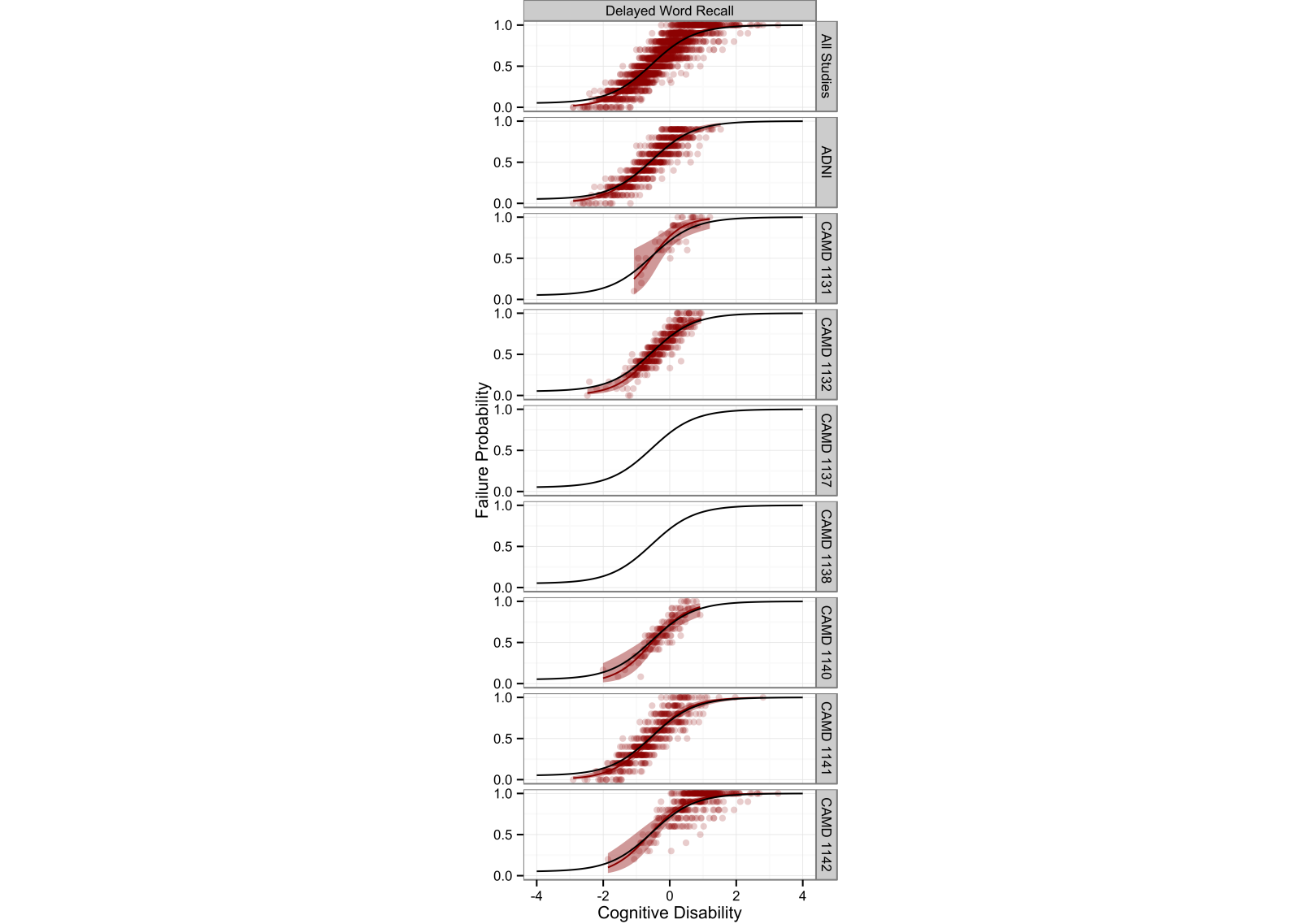


Figure B.19: ICC curves from the IRT model fit (black line) compared to the fit of a generalized additive model (GAM ) with cross-validated cubic spline as a smoothing function (dark red line with 95% confidence interval in light red). Red dots are the observed scores.


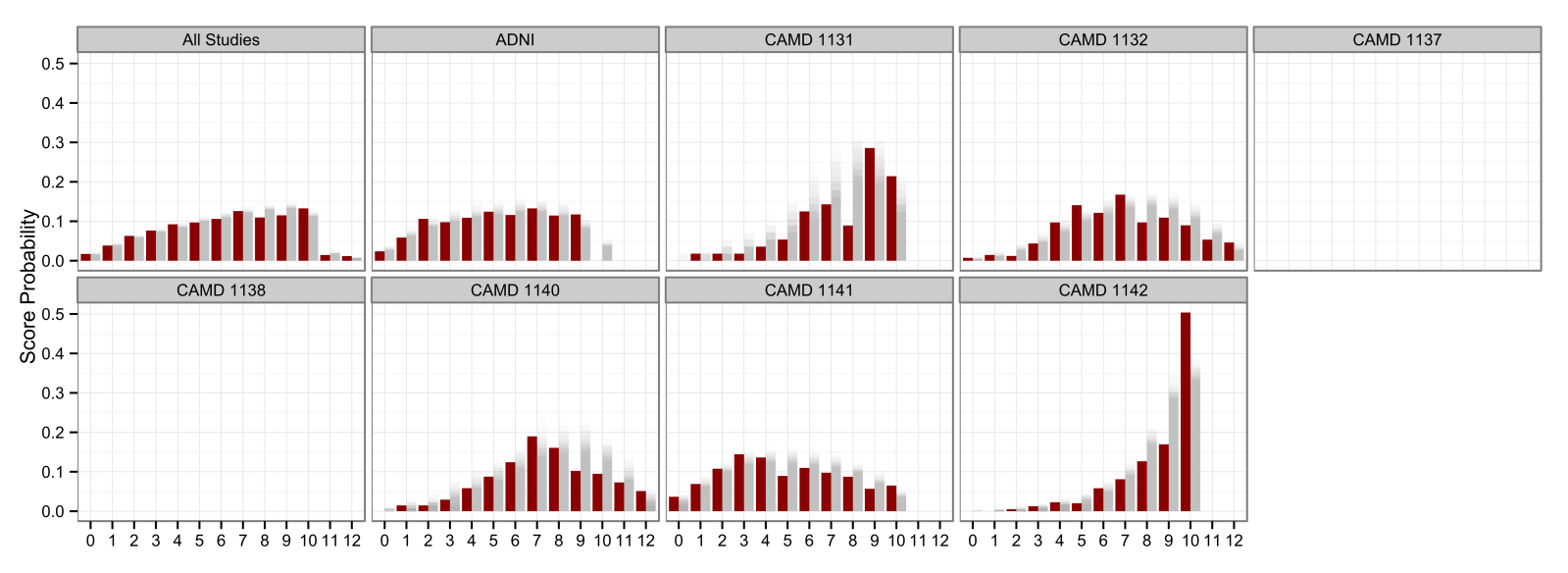
 Figure B.20: Observed (dark red) and simulated (grey) distribution of scores for the delayed word recall component. The grey shading visualizes the variability from 100 repetitions of the simulations.

**Word Recognition**

**Studies without test repetition**


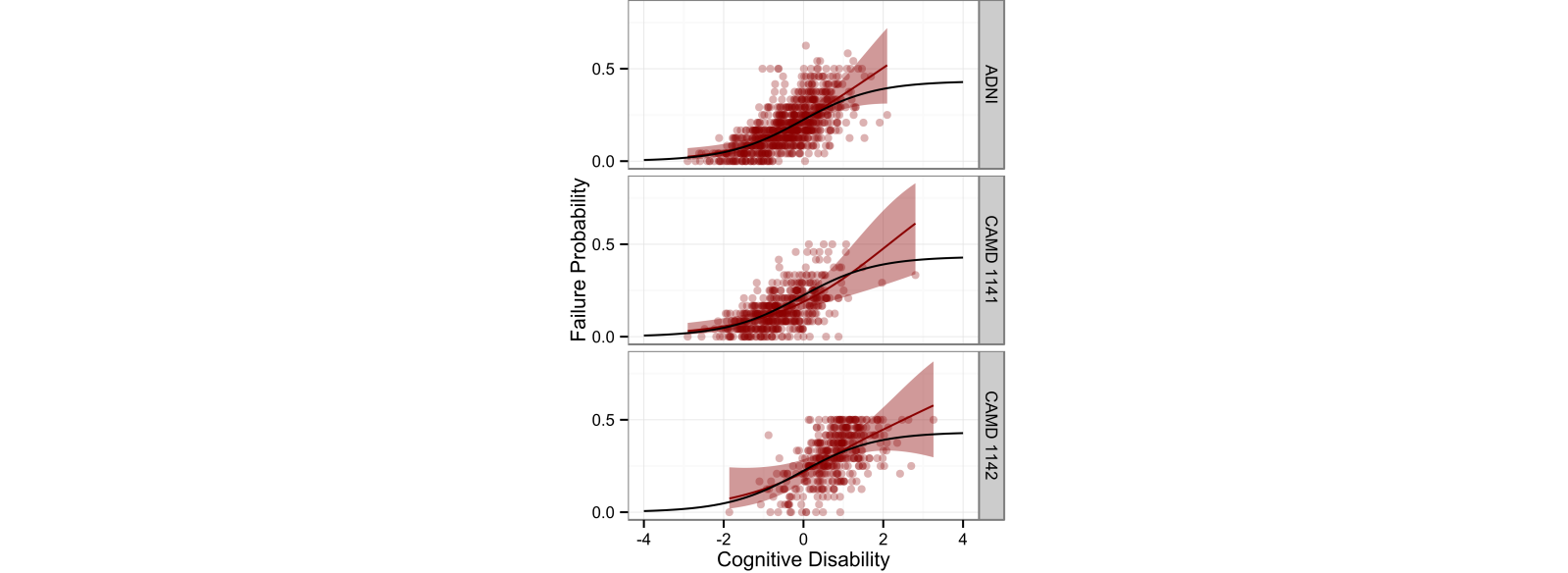
Figure B.21: ICC curves from the IRT model fit (black line) compared to the fit of a generalized additive model (GAM ) with cross-validated cubic spline as a smoothing function (dark red line with 95% confidence interval in light red). Red dots are the observed scores.


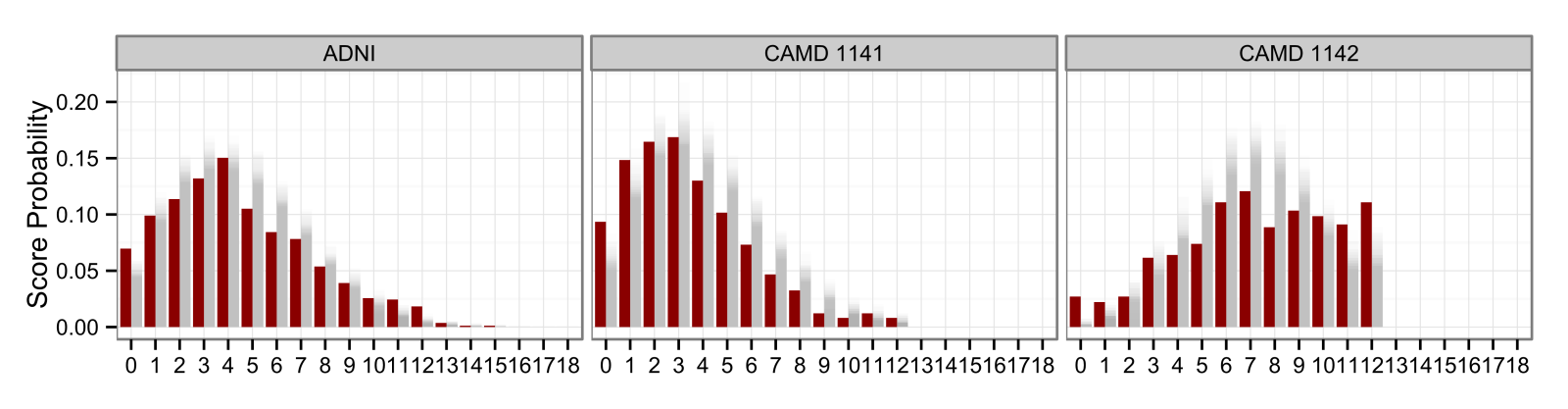


Figure B.22: Observed (dark red) and simulated (grey) distribution of scores for the word recognition component (studies without repetition). The grey shading visualizes the variability from 100 repetitions of the simulations.

**Studies with test repetition**


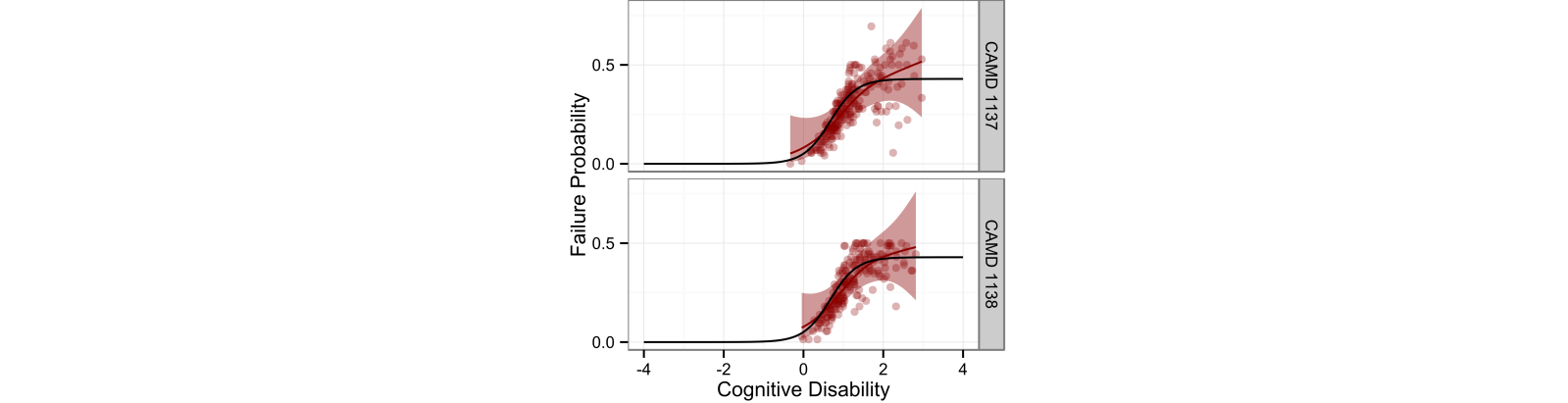


Figure B.23: ICC curves from the IRT model fit (black line) compared to the fit of a generalized additive model (GAM ) with cross-validated cubic spline as a smoothing function (dark red line with 95% confidence interval in light red). Red dots are the observed scores.


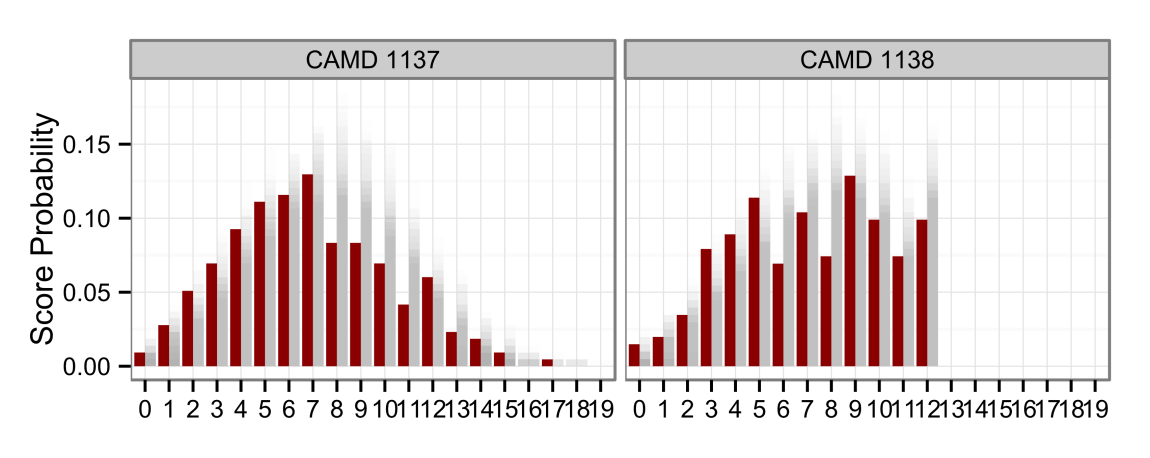


Figure B.24: Observed (dark red) and simulated (grey) distribution of scores for the word recognition component (studies with repetition). The grey shading visualizes the variability from 100 repetitions of the simulations.

**Number Cancellation**


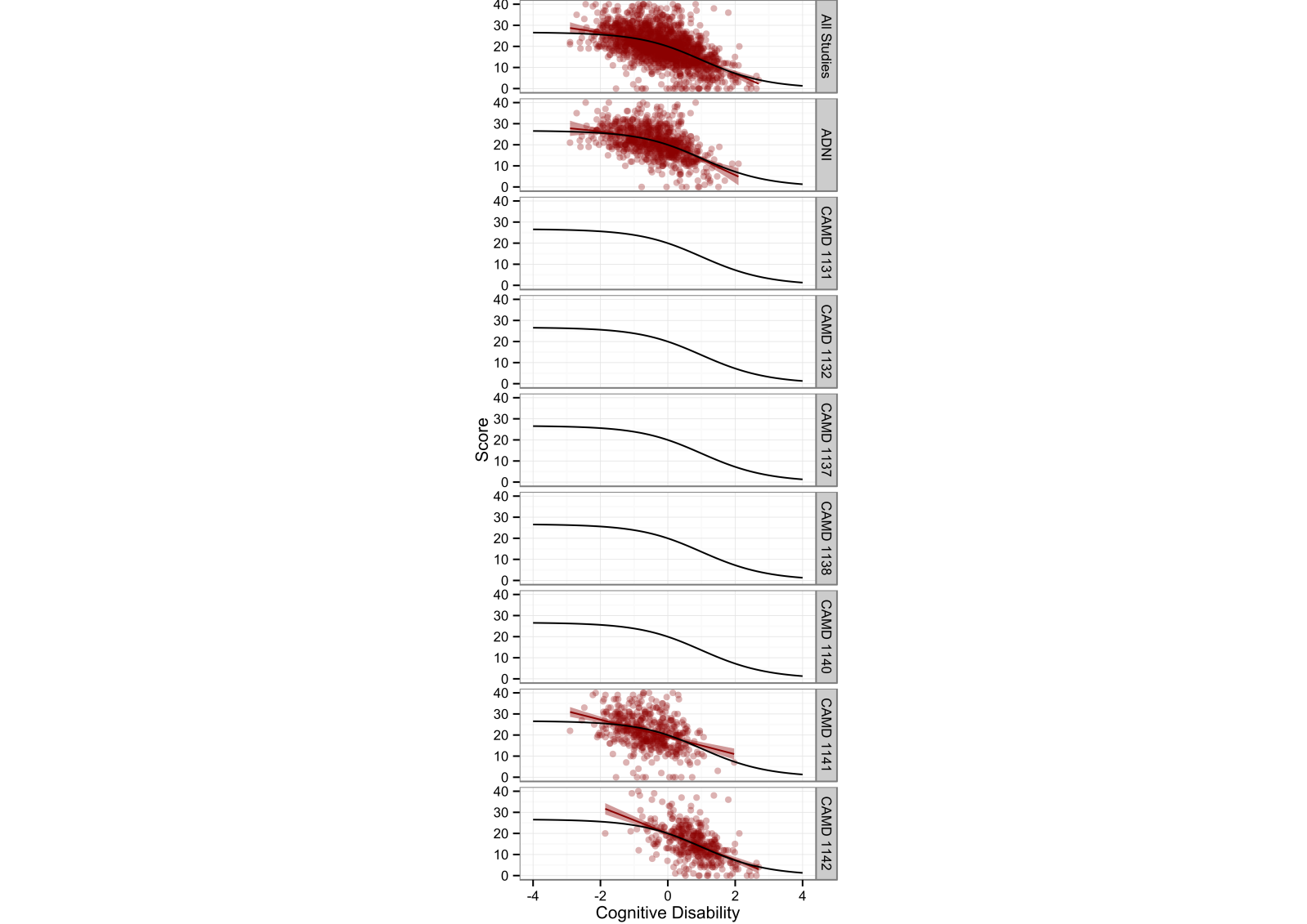


Figure B.25: ICC curves from the IRT model fit (black line) compared to the fit of a generalized additive model (GAM ) with cross-validated cubic spline as a smoothing function (dark red line with 95% confidence interval in light red). Red dots are the observed scores.


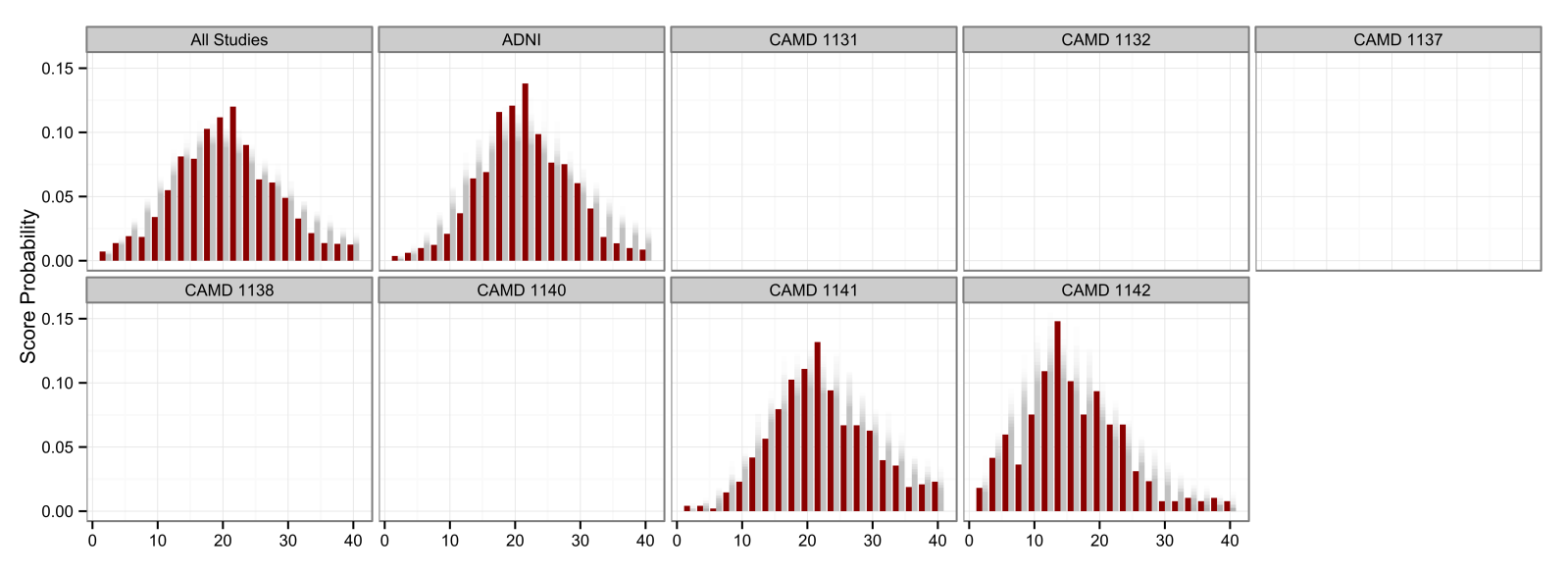


Figure B.26: Observed (dark red) and simulated (grey) distribution of scores for the number cancellation component. The grey shading visualizes the variability from 100 repetitions of the simulations.

**Comprehension**


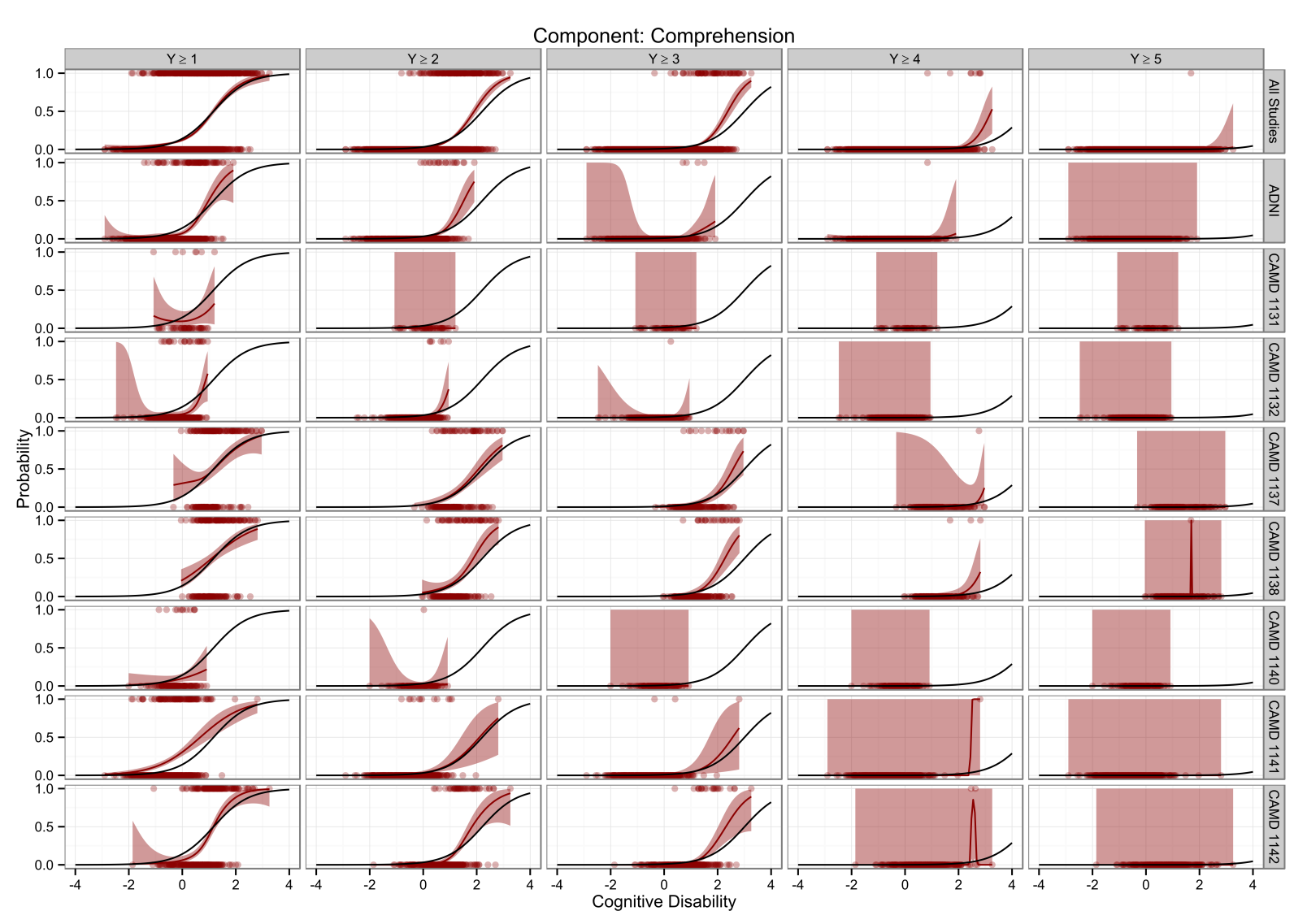


Figure B.27: ICC curves from the IRT model fit (black line) compared to the fit of a generalized additive model (GAM ) with cross-validated cubic spline as a smoothing function (dark red line with 95% confidence interval in light red). Red dots are the observed scores.


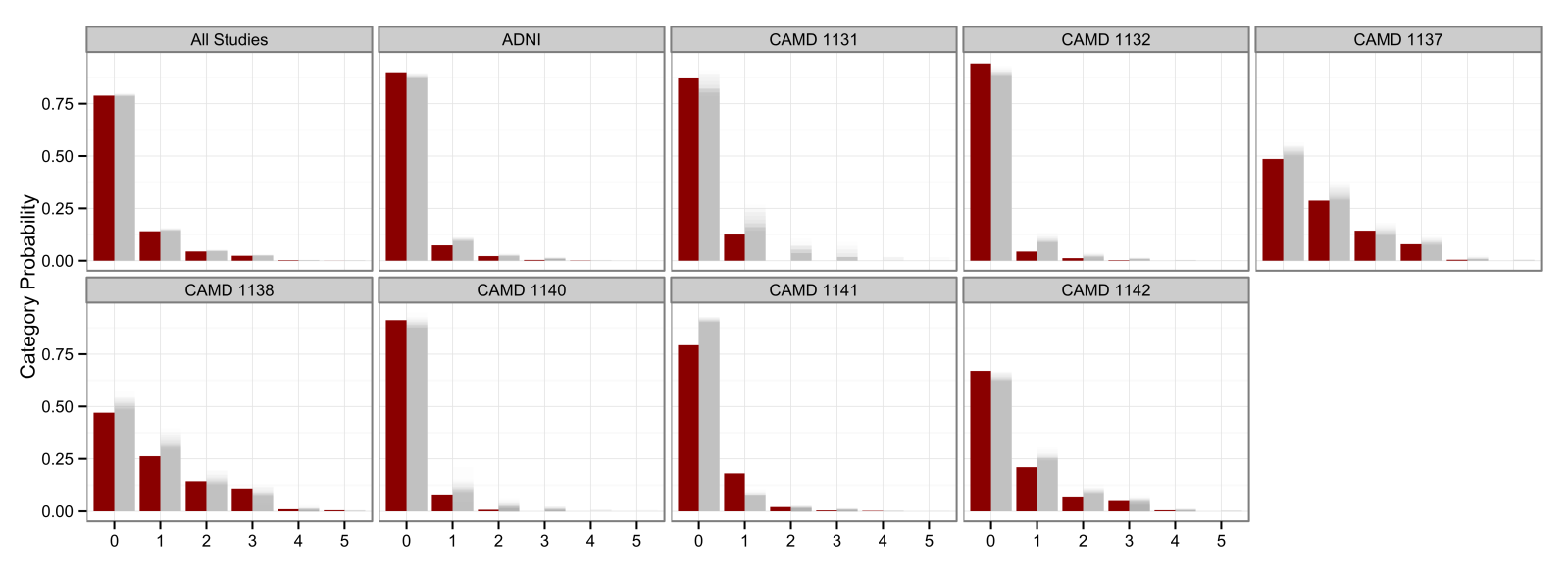


Figure B.28: Observed (dark red) and simulated (grey) fraction of subjects with a certain categorization for the comprehension component. The grey shading visualizes the variability from 100 repetitions of the simulations.

**Remembering**


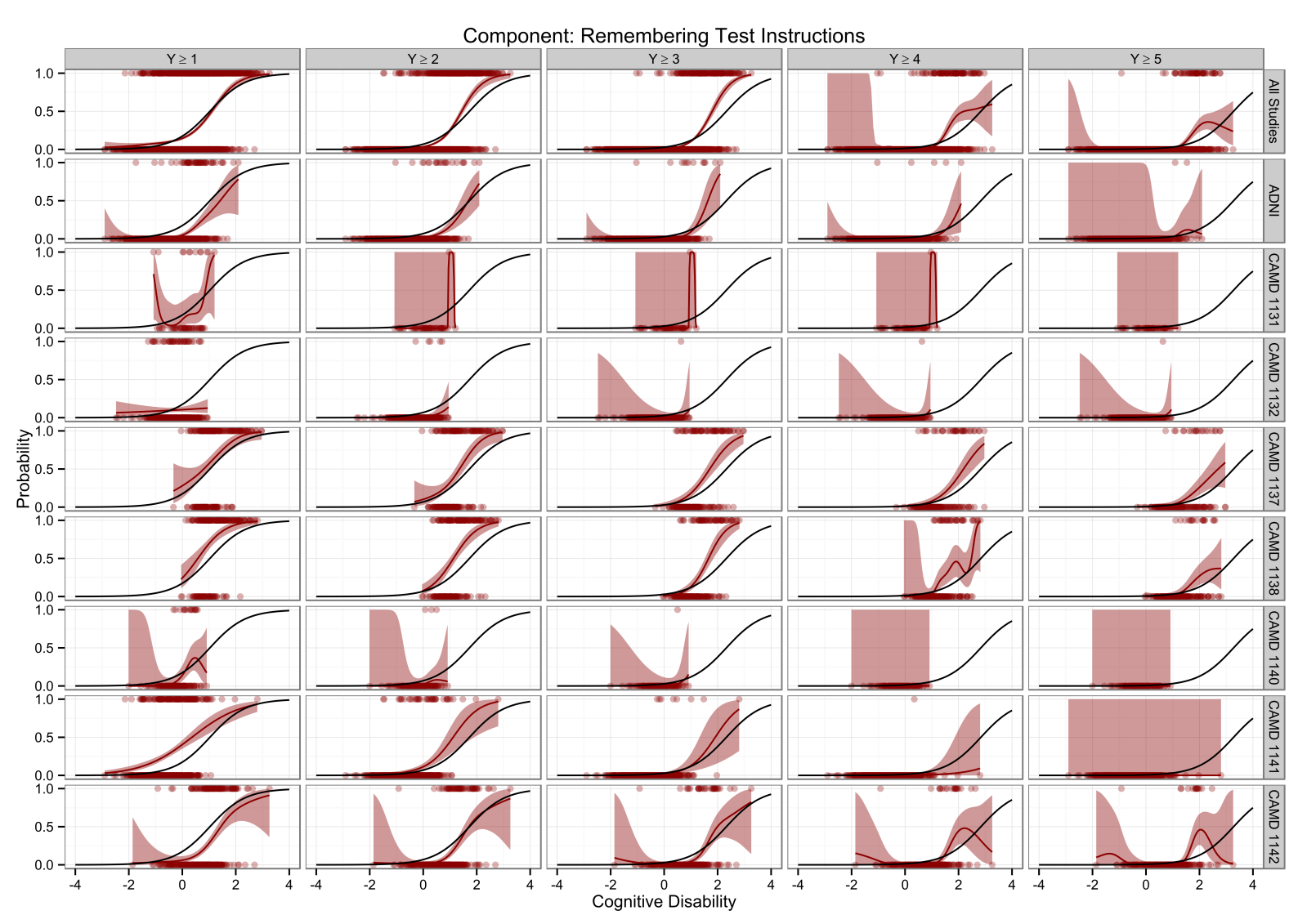


Figure B.29: ICC curves from the IRT model fit (black line) compared to the fit of a generalized additive model (GAM ) with cross-validated cubic spline as a smoothing function (dark red line with 95% confidence interval in light red). Red dots are the observed scores.


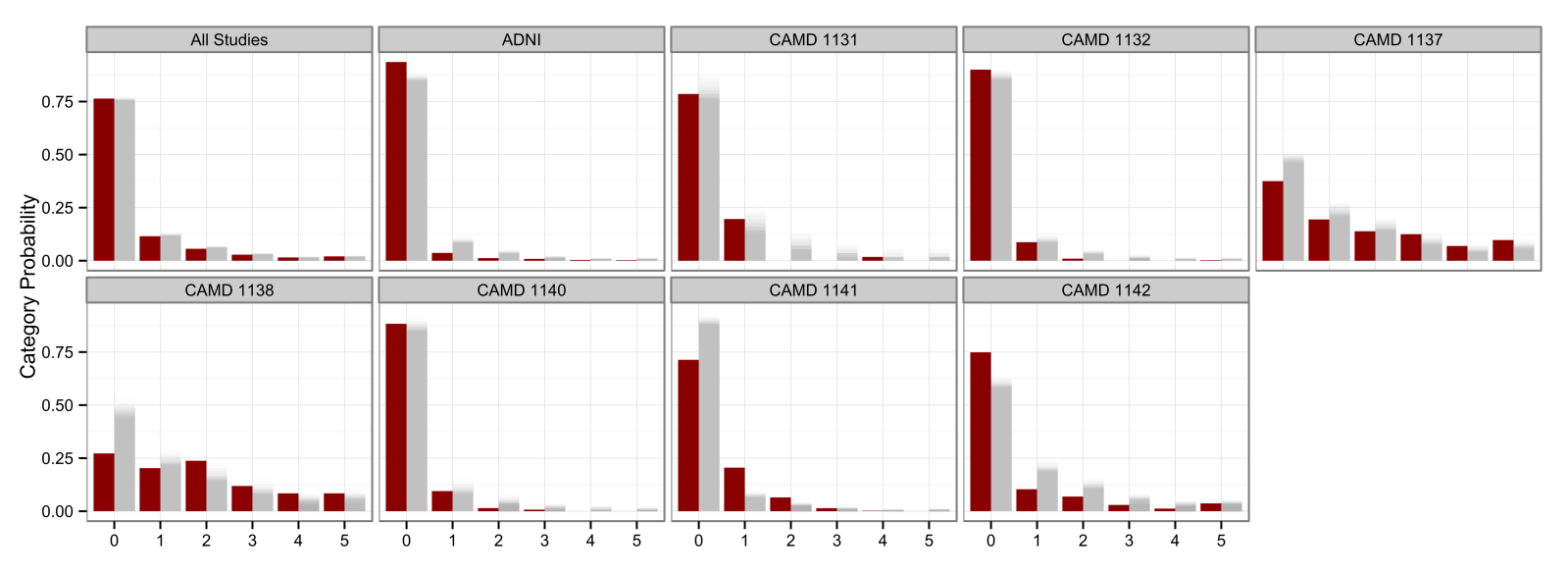


Figure B.30: Observed (dark red) and simulated (grey) fraction of subjects with a certain categorization for the remembering component. The grey shading visualizes the variability from 100 repetitions of the simulations.

**Spoken Language**


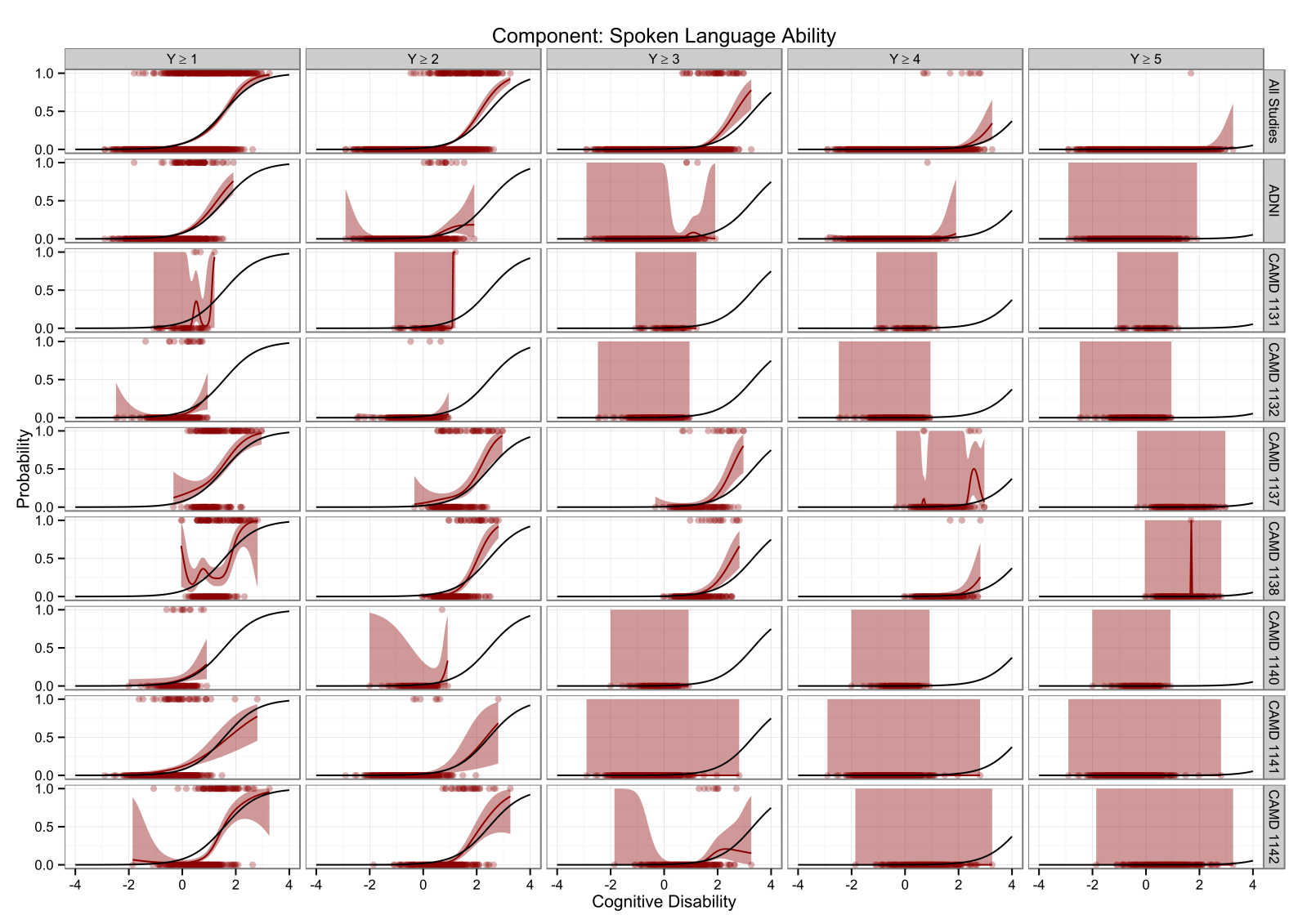


Figure B.31: ICC curves from the IRT model fit (black line) compared to the fit of a generalized additive model (GAM ) with cross-validated cubic spline as a smoothing function (dark red line with 95% confidence interval in light red). Red dots are the observed scores.
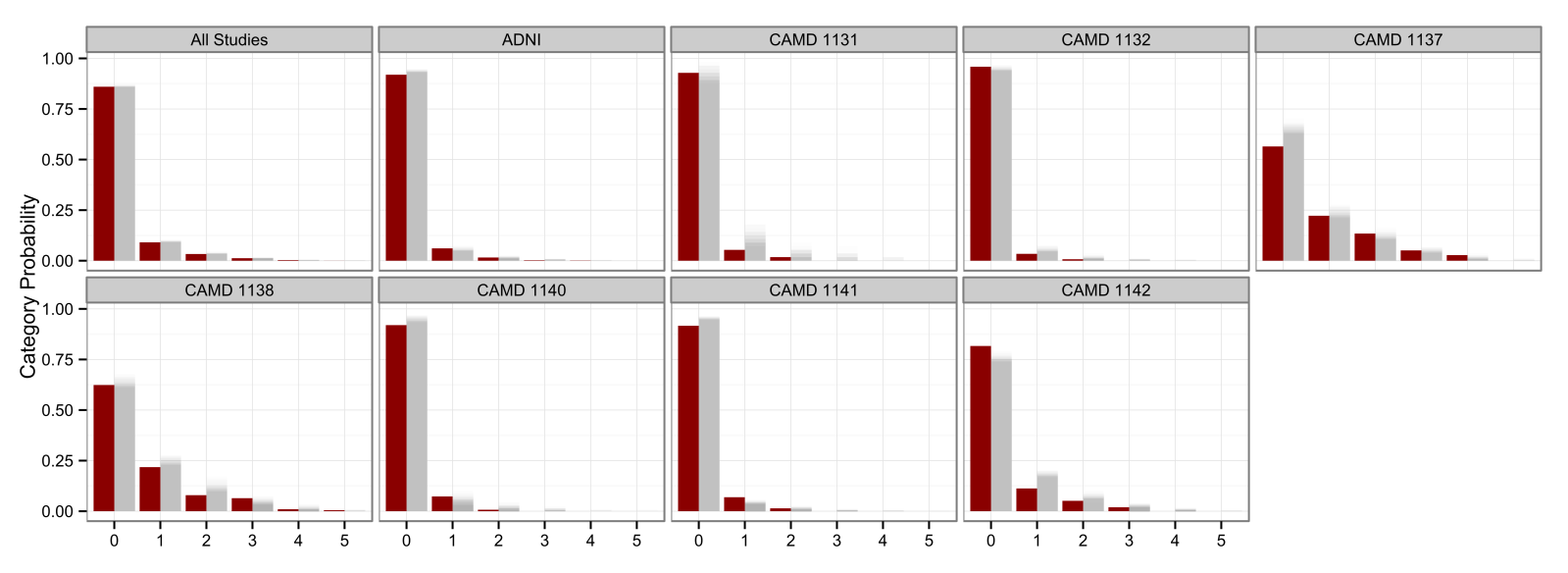


Figure B.32: Observed (dark red) and simulated (grey) fraction of subjects with a certain categorization for the spoken language component. The grey shading visualizes the variability from 100 repetitions of the simulations.

**Word Finding**


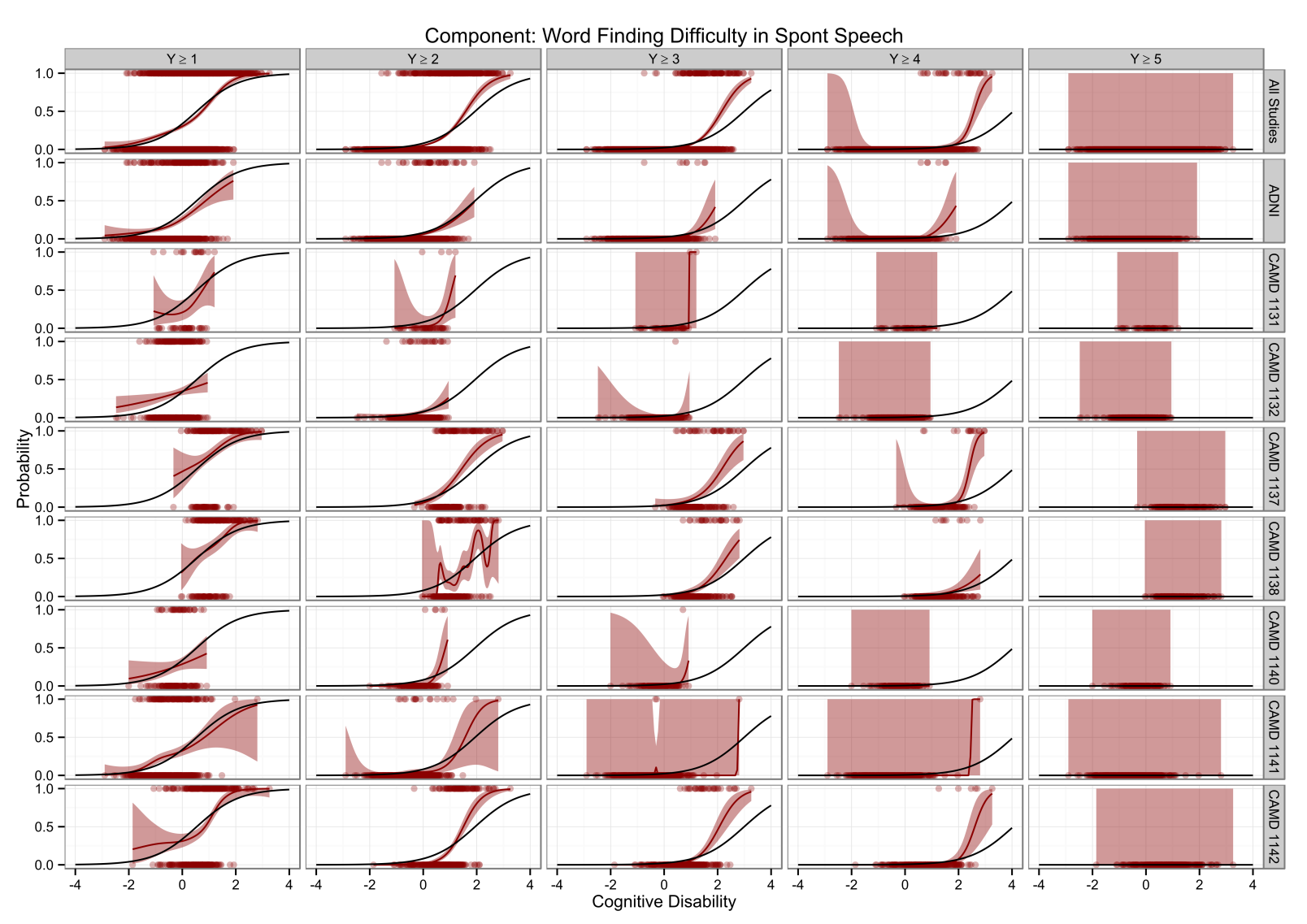


Figure B.33: ICC curves from the IRT model fit (black line) compared to the fit of a generalized additive model (GAM ) with cross-validated cubic spline as a smoothing function (dark red line with 95% confidence interval in light red). Red dots are the observed scores.
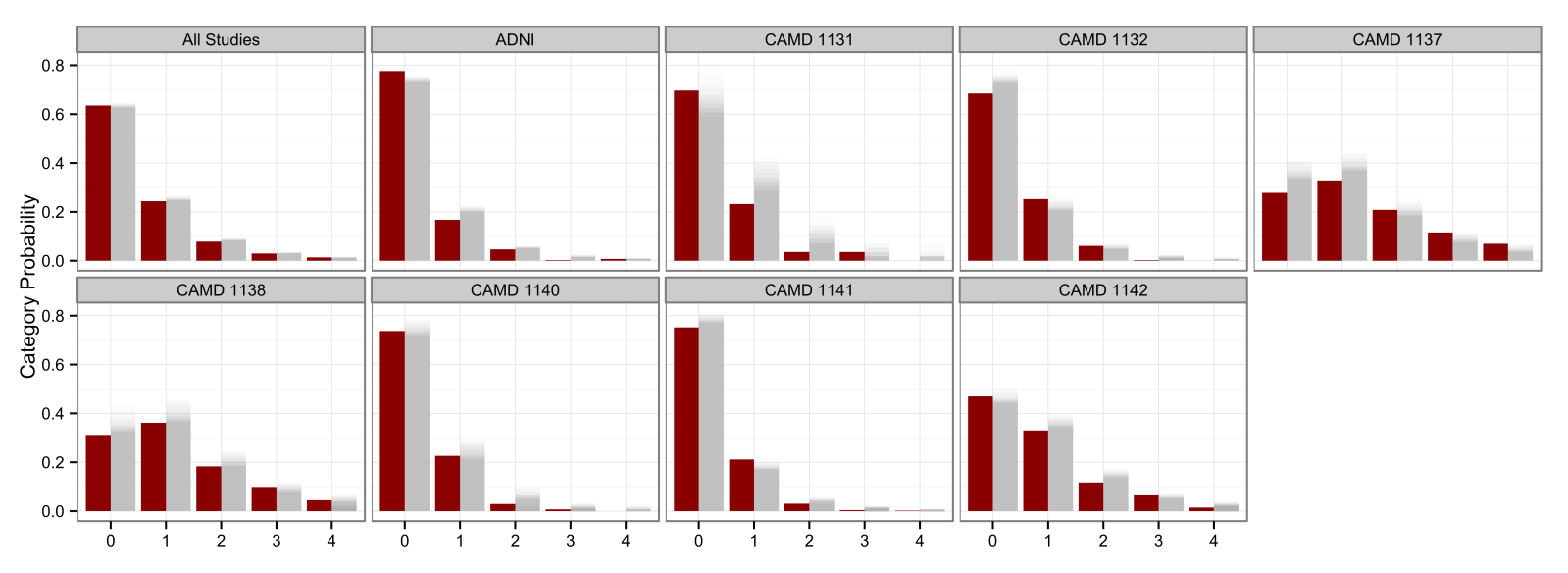


Figure B.34: Observed (dark red) and simulated (grey) fraction of subjects with a certain categorization for the word finding component. The grey shading visualizes the variability from 100 repetitions of the simulations.
