# Supplementary material for: Improved Utilization of ADAS-Cog Assessment Data Through Item Response Theory Based Pharmacometric Modeling
Source: Pharm Res. 2014 Mar 5;31(8):2152–65. doi: 10.1007/s11095-014-1315-5 (PMC4153970; doi:10.1007/s11095-014-1315-5)
Supplement: Supplementary file 3 — (DOCX 1237 kb) [file 11095_2014_1315_MOESM3_ESM.docx]

Supplement C: Item Level VPCs

The supplement presents visual predictive checks (VPCs) for the longitudinal IRT model and all items in the ADAS-cog 11 assessment. The figures are ordered by response type, starting with the binary components, then the count components and finally the ordered categorical components.

The following 5 figures show VPC for the binary response components. With the exception of the “Naming Objects & Fingers” component, predictions and observations are in a very good agreement. The “Naming Objects & Fingers” is the most complex component of the assessment and possibly the hardest to standardize. Factors such as the language of the patient or whether she/he is naïve to the ADAS-cog assessment might influence the result and are worth exploring in future work. However, the remaining differences between observed and predicted item level responses were judged acceptable as their contribution to the overall score is minor. For example a hypothetical difference of 100% between the observed and predicted fraction of subjects translates to a difference of less than 0.02 points of the total ADAS-cog.

| **Commands**  (1: “Make a fist”, 2: “Point to ceiling”, 3: “Put pencil”, 4: “Put watch”, 5: “Tap shoulder”)**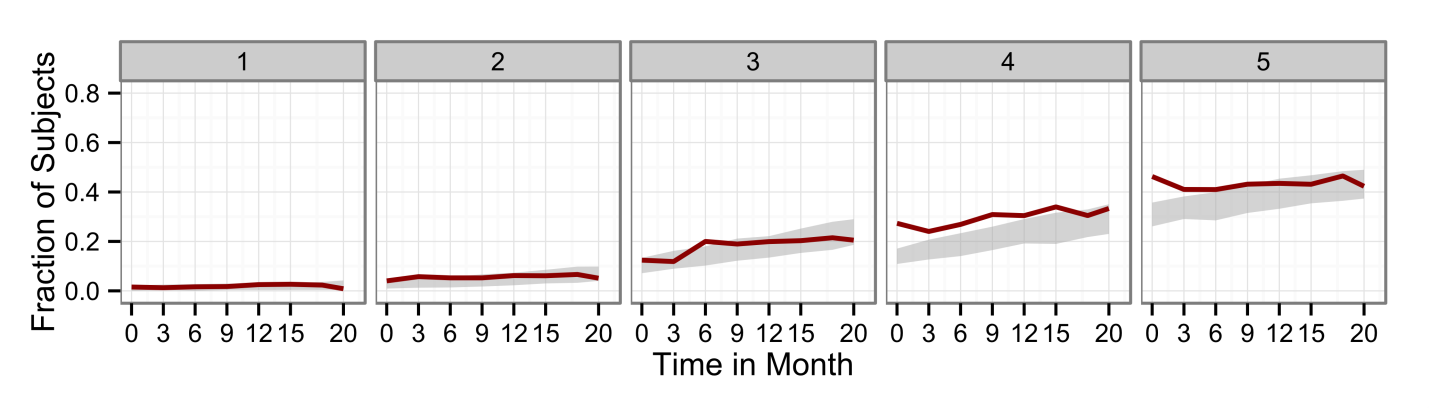**  Figure C.1: Fraction of subjects in the study failing to perform the specified task as observed in the data (red lines) and as predicted by the model (95% confidence interval in gray). |
| --- |
| **Construction**  (1: “Circle”, 2: “Rectangles”, 3: “Rhombus”, 4: “Cube”)  **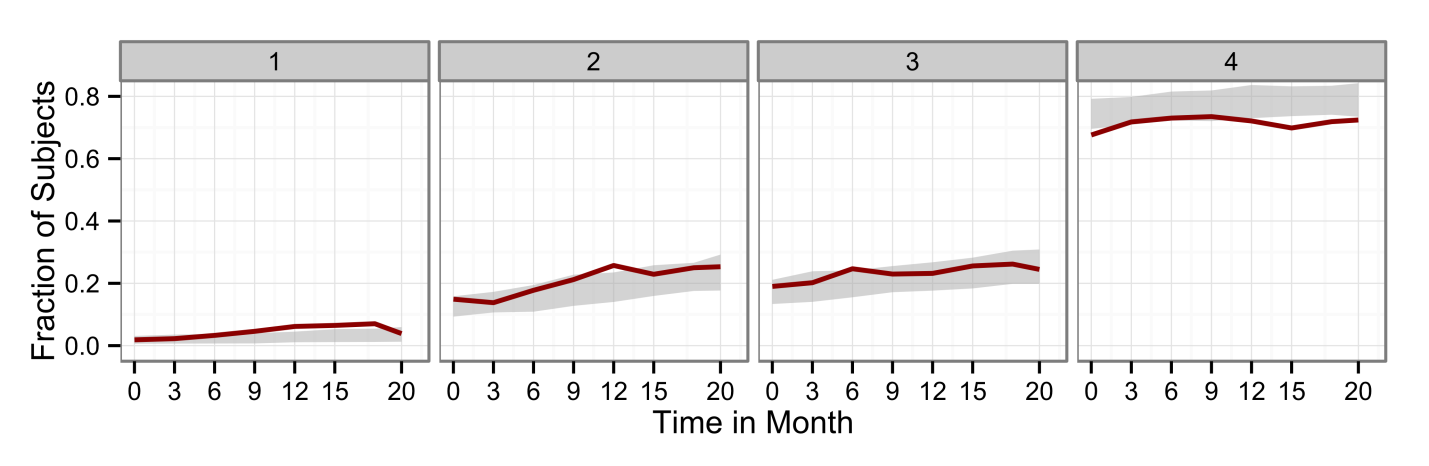**  Figure C.2: Fraction of subjects in the study failing to perform the specified task as observed in the data (red lines) and as predicted by the model (95% confidence interval in gray). |
| **Ideational Praxis**  (1: “Fold letter”, 2: “Put letter”, 3: “Seal envelope”, 4: “Address envelope”, 5: “Indicate stamp”) **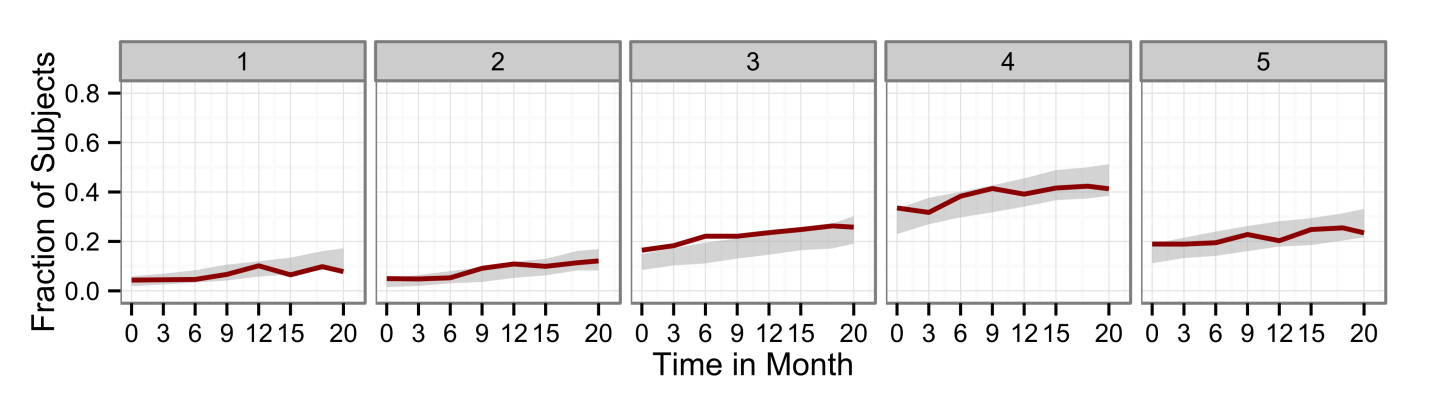**  Figure C.3: Fraction of subjects in the study failing to perform the specified task as observed in the data (red lines) and as predicted by the model (95% confidence interval in gray). |
| **Naming Objects & Fingers**  (1-5: “Fingers”, 6-17: “Objects”)  **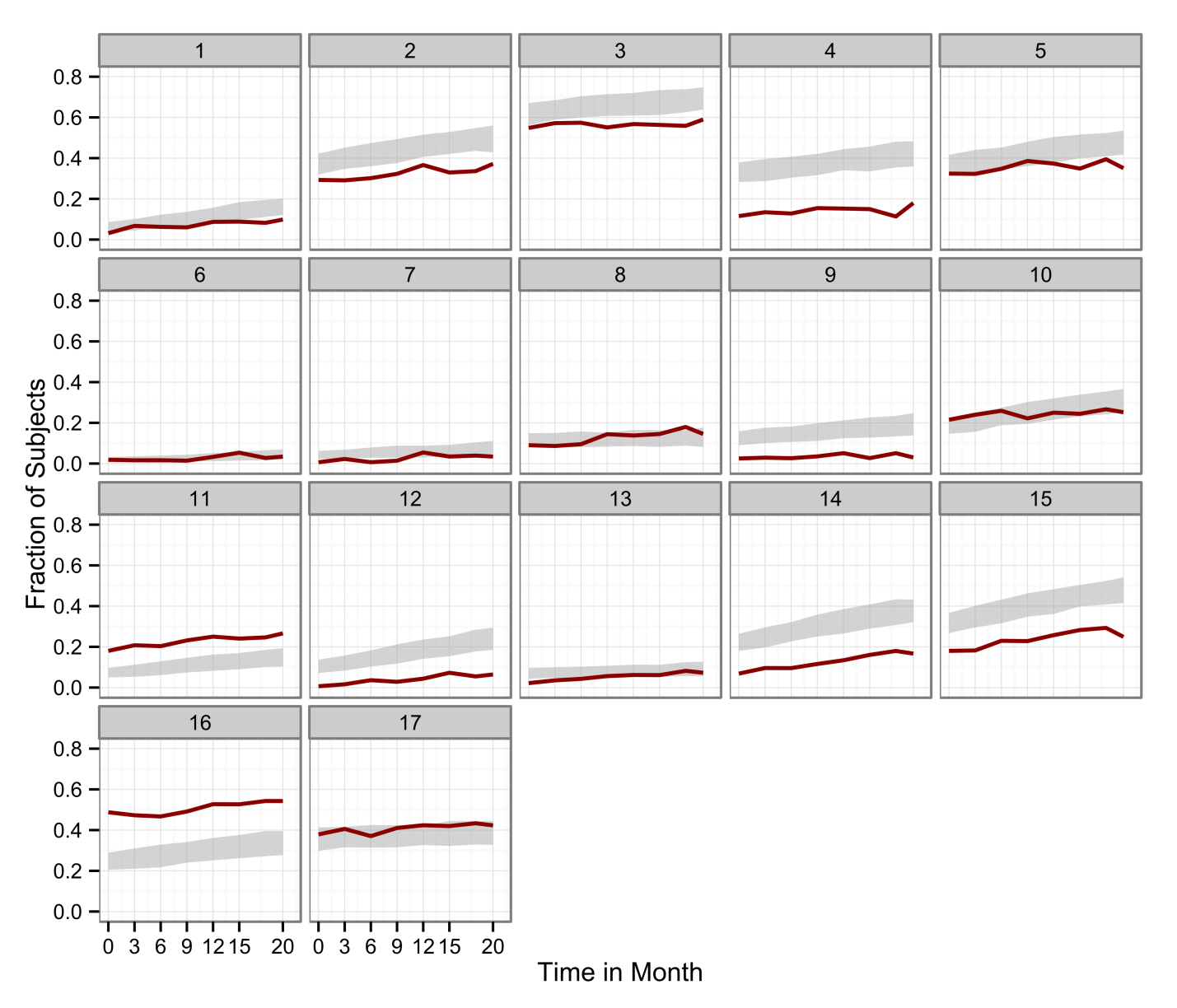**  Figure C.4: Fraction of subjects in the study failing to perform the specified task as observed in the data (red lines) and as predicted by the model (95% confidence interval in gray). |
| **Orientation**  (1: “Name”, 2: “Month”, 3: “Date”, 4: “Year”, 5: “Day”, 6: “Season”, 7: “Place”, 8: “Time”)  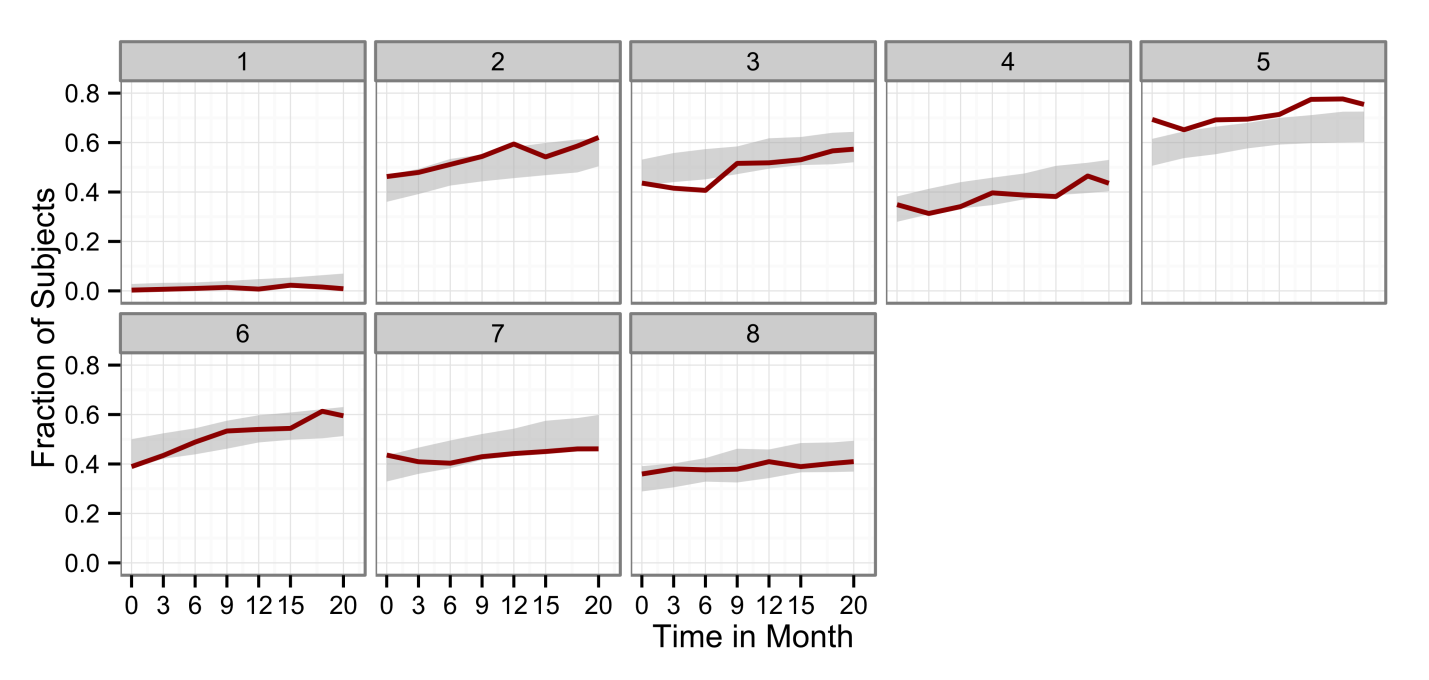  Figure C.5: Fraction of subjects in the study failing to perform the specified task as observed in the data (red lines) and as predicted by the model (95% confidence interval in gray). |

The following 2 figures show VPCs for the “Word Recall” and “Word Recognition” components. The observed fraction is shown as red line while the 95% confidence interval from the model simulations is displayed as a gray ribbon. For both components predictions and observations are in a good agreement.

| **Word Recall**  **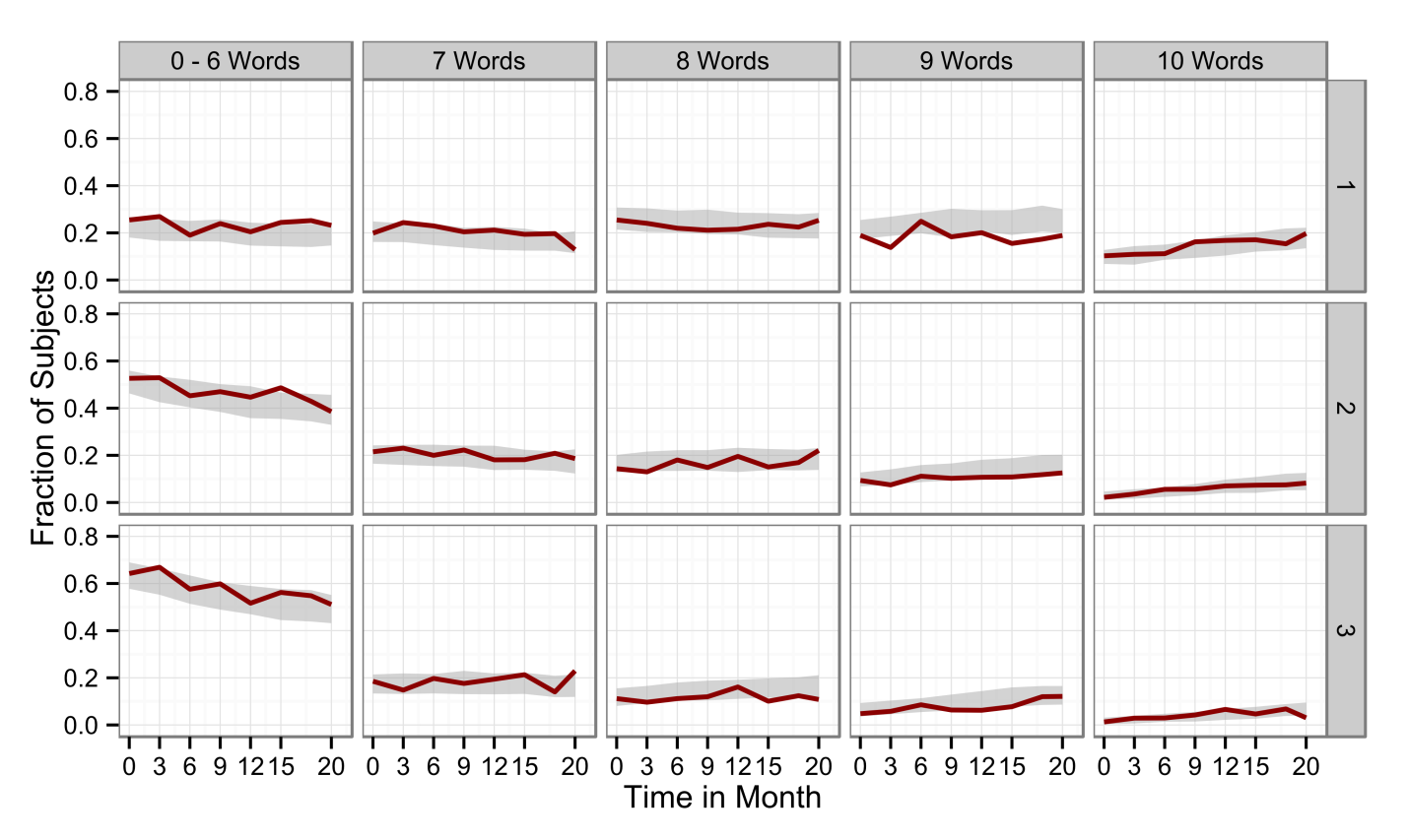**  Figure C.6: Fraction of subjects in the study unable to recall a certain number word as observed in the data (red lines) and as predicted by the model (95% confidence interval in gray). |
| --- |
| **Word Recognition**  **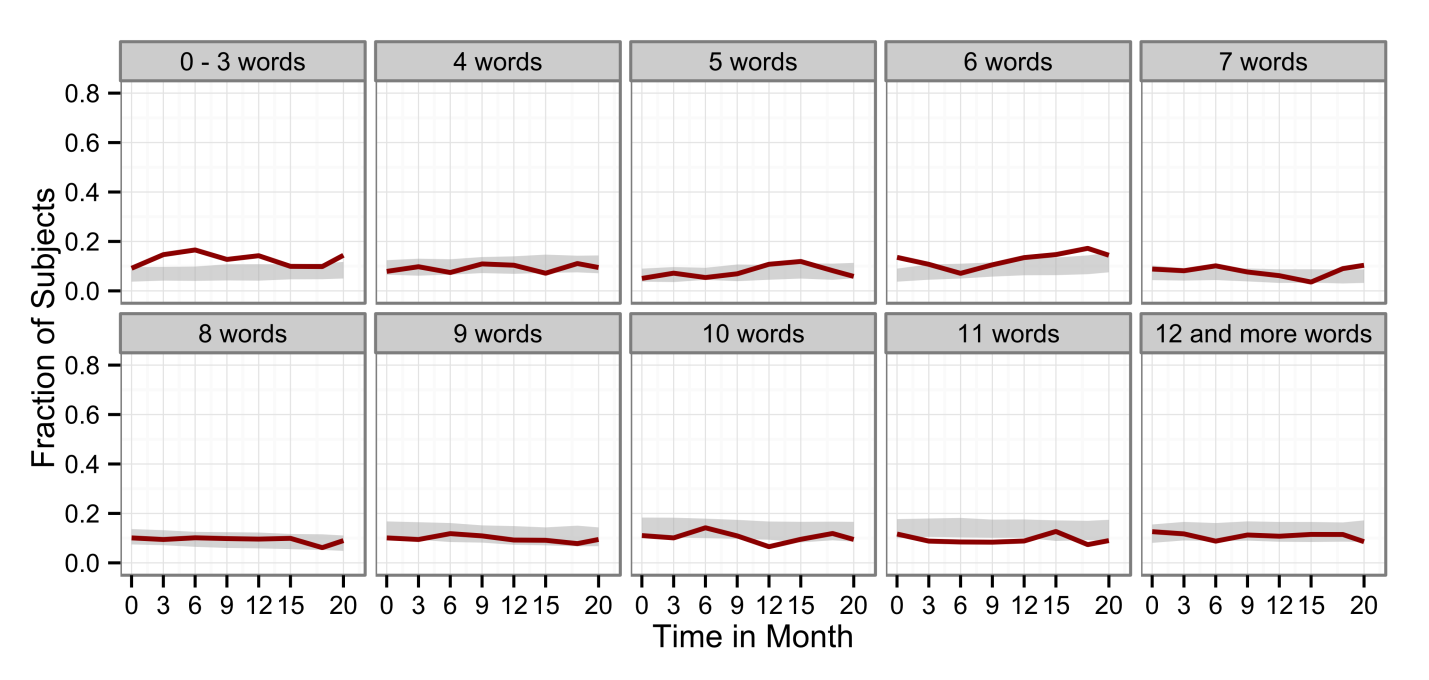**  Figure C.7: Fraction of subjects in the study unable to recognize a certain number word as observed in the data (red lines) and as predicted by the model (95% confidence interval in gray). |

The following 4 figures show VPCs for rater assessed subcomponents of the ADAS-cog assessment. Except for the spoken language component, predictions and observations are in good agreement. A possible explanation for the mismatch in “Spoken Language” is the effect of between rater variability that was not investigated in this work. Inter-study variability is another factor, which could explain the differences. However, due to the small influence of the difference on the overall ADAS-cog prediction, the discrepancy was judged acceptable.

| **Comprehension**  **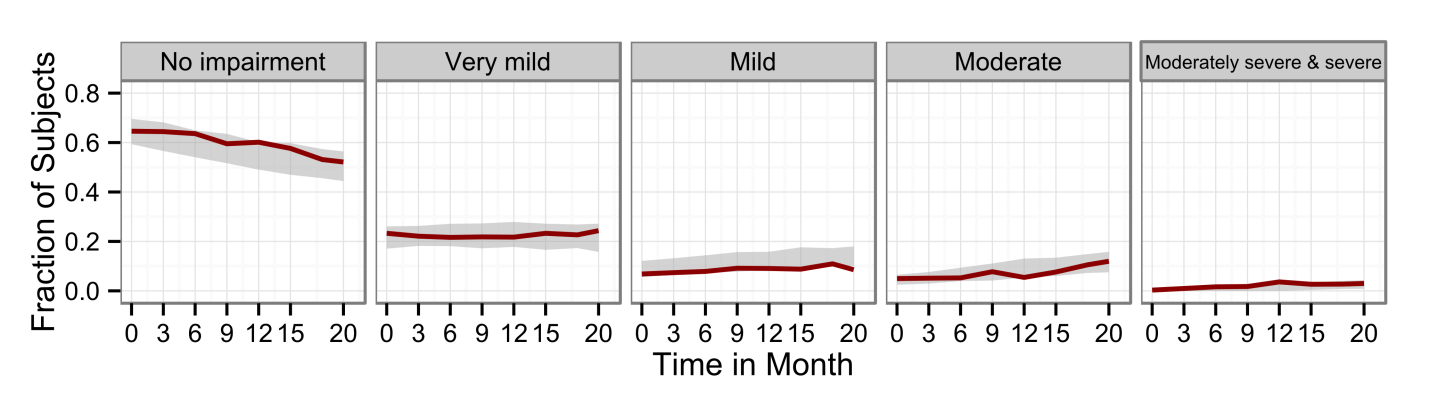**  Figure C.8: Fraction of subjects in the study assigned to a certain category as observed in the data (red lines) and as predicted by the model (95% confidence interval in gray). |
| --- |
| **Spoken Language**  **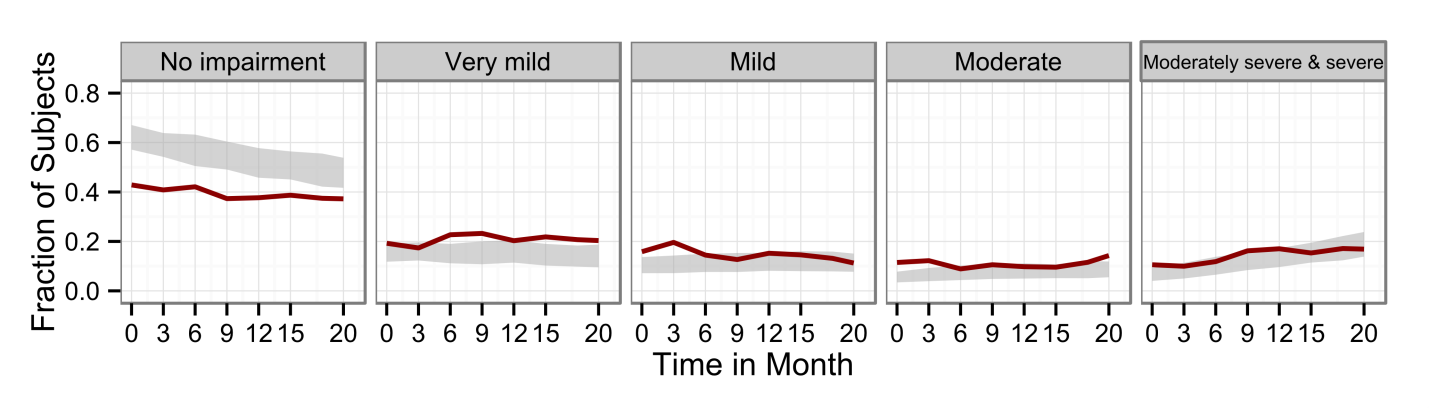**  Figure C.9: Fraction of subjects in the study assigned to a certain category as observed in the data (red lines) and as predicted by the model (95% confidence interval in gray). |
| **Remembering**  **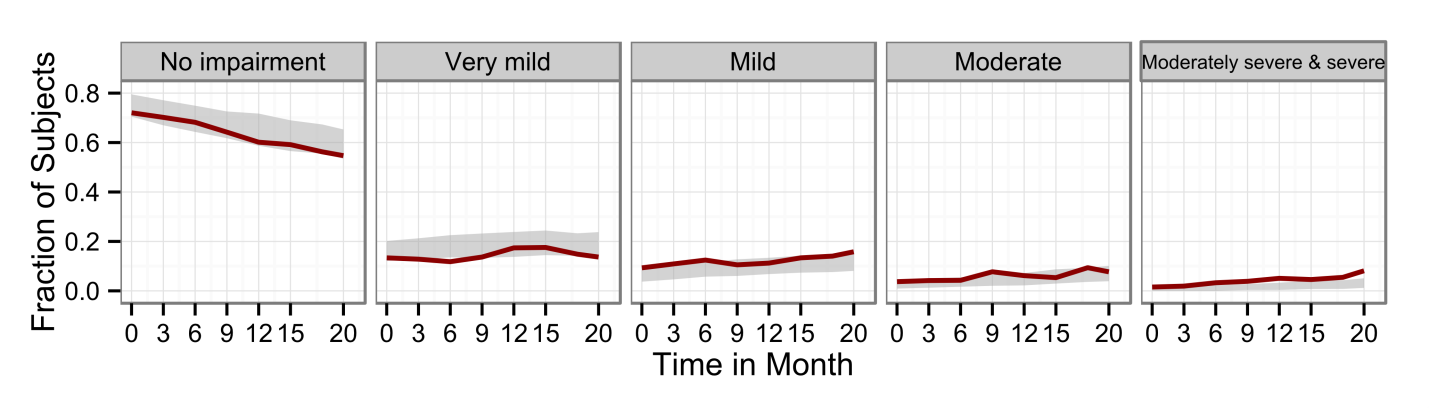**  Figure C.10: Fraction of subjects in the study assigned to a certain category as observed in the data (red lines) and as predicted by the model (95% confidence interval in gray). |
| **Word Finding**  **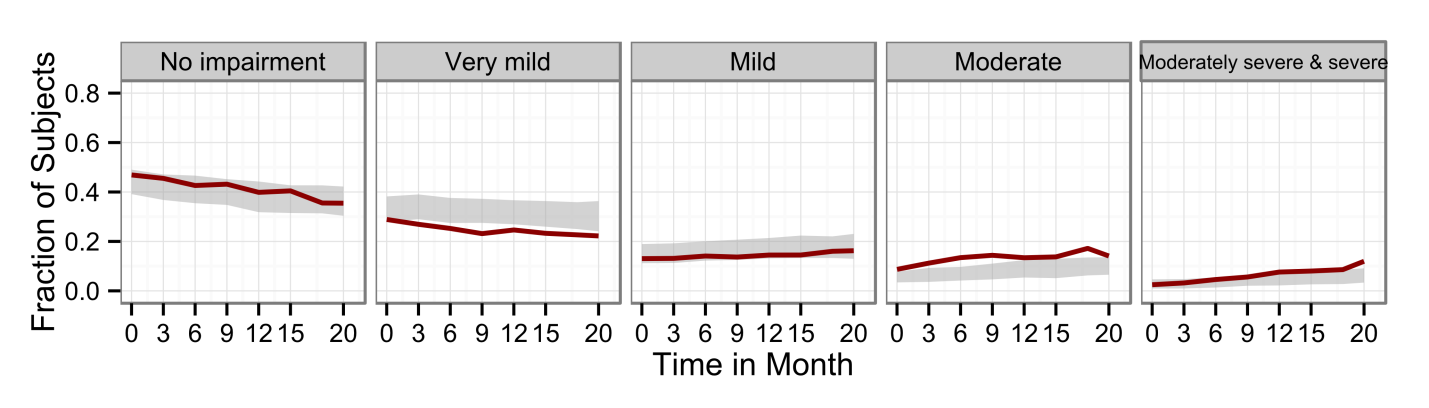**  Figure C.11: Fraction of subjects in the study assigned to a certain category as observed in the data (red lines) and as predicted by the model (95% confidence interval in gray). |
