# Supplementary material for: Improved Utilization of ADAS-Cog Assessment Data Through Item Response Theory Based Pharmacometric Modeling
Source: Pharm Res. 2014 Mar 5;31(8):2152–65. doi: 10.1007/s11095-014-1315-5 (PMC4153970; doi:10.1007/s11095-014-1315-5)
Supplement: Supplementary file 4 — (DOCX 1073 kb) [file 11095_2014_1315_MOESM4_ESM.docx]

Supplement D: Total ADAS-cog Score Model & Diagnostics

Based on a simulated dataset from the longitudinal IRT model described in the manuscript, a pharmacometric total ADAS-cog score model was built. The best model described a linear increase of the total ADAS-cog score with time, and used a box-cox distribution to model the individual baseline scores and normally distributed individual slope parameters which were correlated with the baseline random effect.

The following figures represent visual predictive checks underlining the ability of the summary score model to describe data simulated from the IRT model.

Simulation dataset 1: 100 subjects in the study

| **Placebo Treatment**  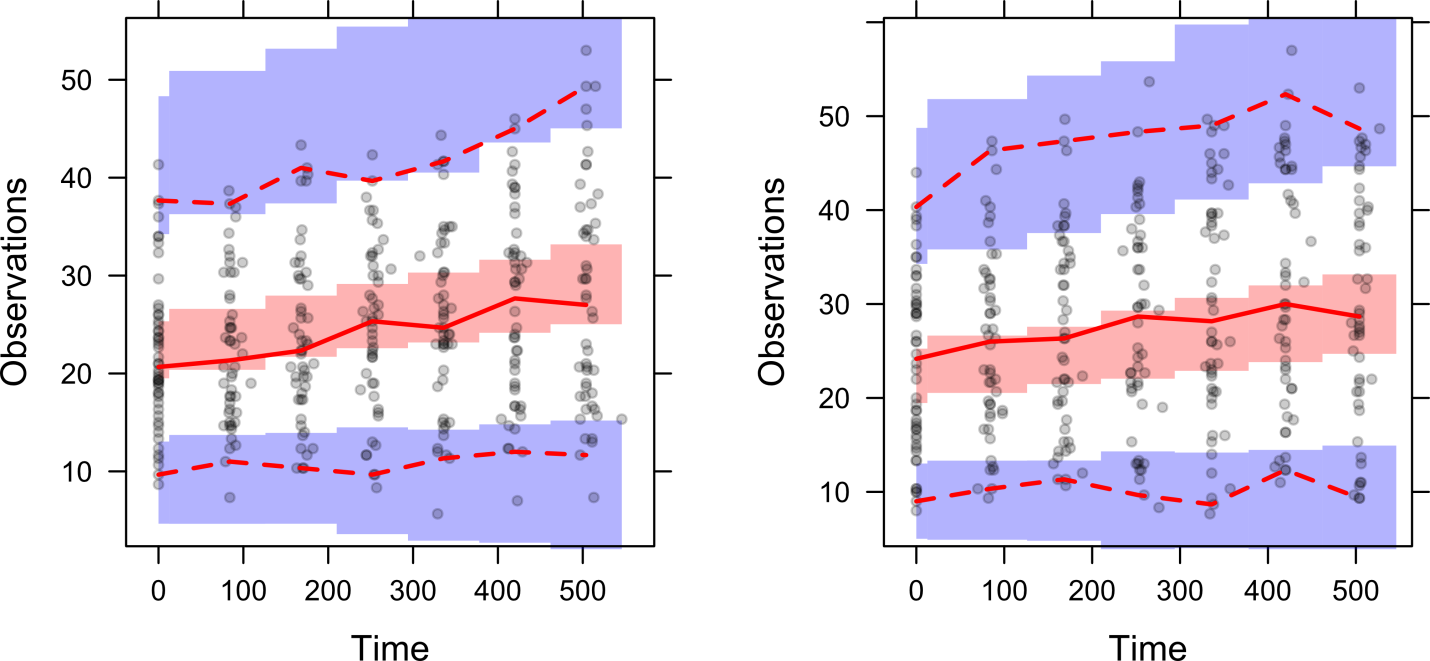  Figure D.1: Visual predictive check for ADAS-cog score (observations) comparing the observed 2.5^th^ and 97.5^th^ (dashed lines) as well as the median (solid line) with the model simulated 95% confidence interval |
| --- |

Simulation dataset 2: 200 subjects in the study

| **Placebo Treatment**  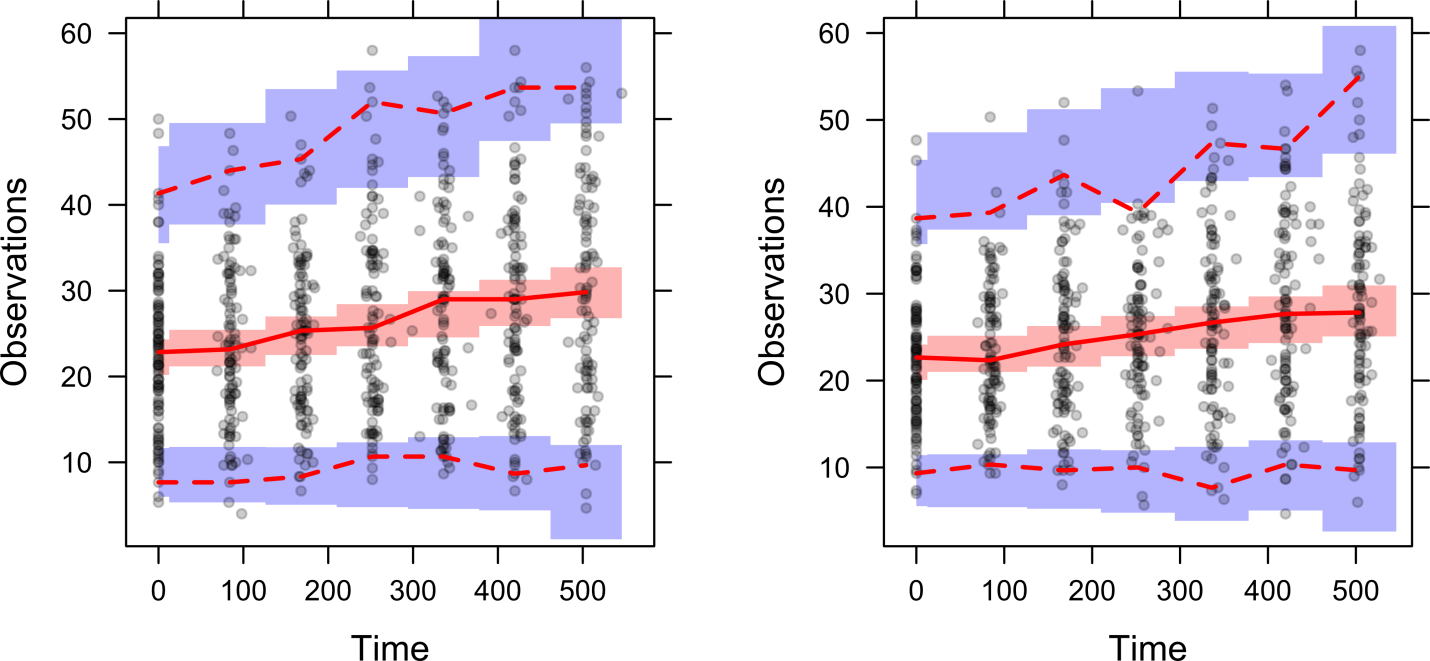  Figure D.2: Visual predictive check for ADAS-cog score (observations) comparing the observed 2.5^th^ and 97.5^th^ (dashed lines) as well as the median (solid line) with the model simulated 95% confidence interval |
| --- |

Simulation dataset 3: 400 subjects in the study

| **Placebo Treatment**  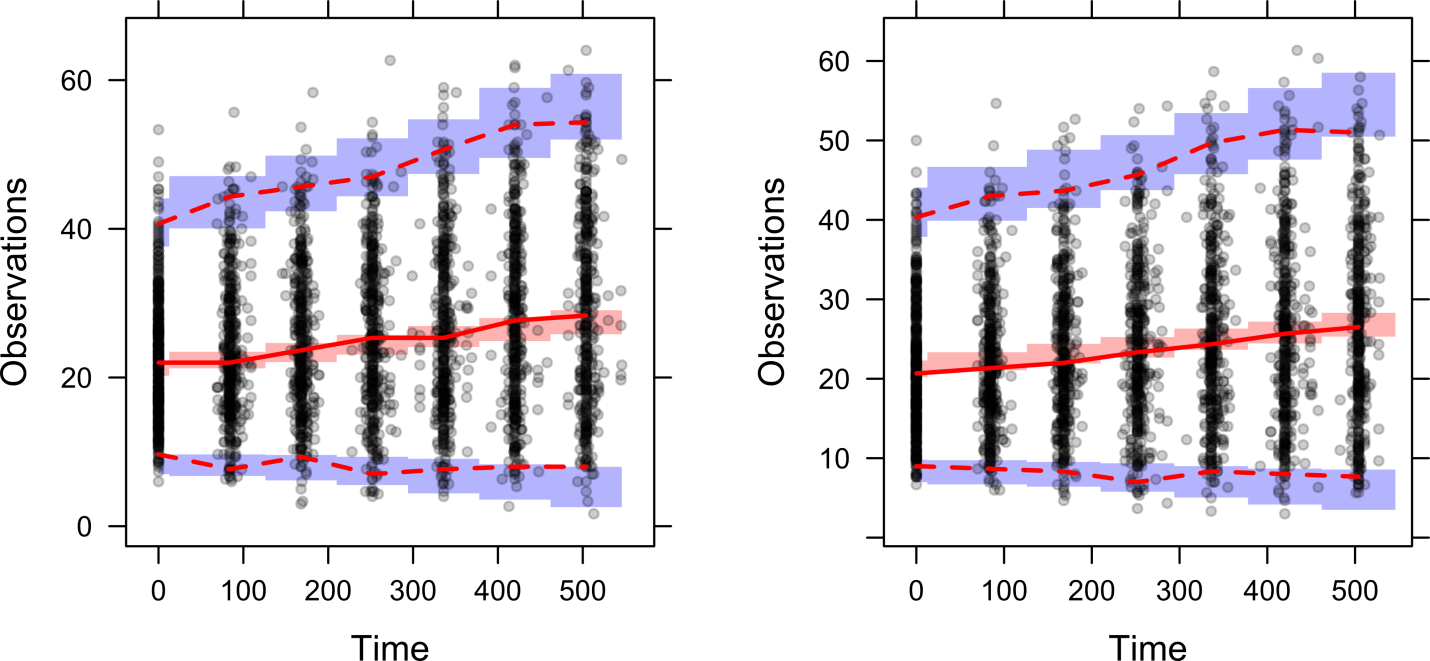  Figure D.3: Visual predictive check for ADAS-cog score (observations) comparing the observed 2.5^th^ and 97.5^th^ (dashed lines) as well as the median (solid line) with the model simulated 95% confidence interval |
| --- |

Simulation dataset 4: 800 subjects in the study

| **Placebo Treatment**  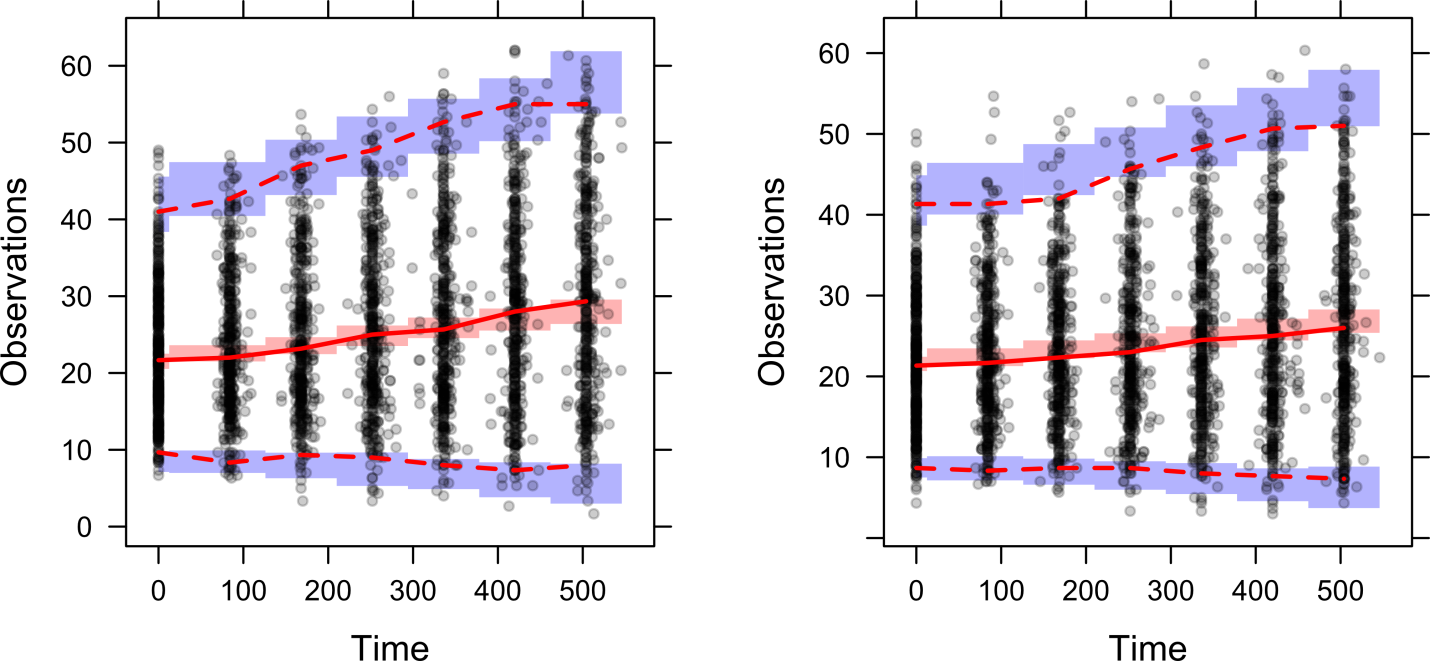  Figure D.4: Visual predictive check for ADAS-cog score (observations) comparing the observed 2.5^th^ and 97.5^th^ (dashed lines) as well as the median (solid line) with the model simulated 95% confidence interval |
| --- |
